# In utero and childhood exposure to tobacco smoke and multi-layer molecular signatures in children

**DOI:** 10.1186/s12916-020-01686-8

**Published:** 2020-08-19

**Authors:** Marta Vives-Usano, Carles Hernandez-Ferrer, Léa Maitre, Carlos Ruiz-Arenas, Sandra Andrusaityte, Eva Borràs, Ángel Carracedo, Maribel Casas, Leda Chatzi, Muireann Coen, Xavier Estivill, Juan R. González, Regina Grazuleviciene, Kristine B. Gutzkow, Hector C. Keun, Chung-Ho E. Lau, Solène Cadiou, Johanna Lepeule, Dan Mason, Inés Quintela, Oliver Robinson, Eduard Sabidó, Gillian Santorelli, Per E. Schwarze, Alexandros P. Siskos, Rémy Slama, Marina Vafeiadi, Eulàlia Martí, Martine Vrijheid, Mariona Bustamante

**Affiliations:** 1 Centre for Genomic Regulation (CRG), The Barcelona Institute of Science and Technology, Barcelona, Spain; 2 ISGlobal, Barcelona, Spain; 3 Universitat Pompeu Fabra (UPF), Barcelona, Spain; 4 CIBER Epidemiología y Salud Pública (CIBERESP), Madrid, Spain; 5 Department of Environmental Sciences, Vytautas Magnus University, K. Donelaicio Street 58, 44248 Kaunas, Lithuania; 6 Grupo de Medicina Xenómica, Fundación Pública Galega de Medicina Xenómica, Instituto de Investigación Sanitaria de Santiago de Compostela (IDIS), SERGAS, Rúa Choupana s/n, 15706 Santiago de Compostela, Spain; 7 Centro de Investigación en Red de Enfermedades Raras (CIBERER) y Centro Nacional de Genotipado (CEGEN-PRB3-ISCIII), Universidade de Santiago de Compostela, Praza do Obradoiro s/n, 15782 Santiago de Compostela, Spain; 8 Department of Preventive Medicine, Keck School of Medicine, University of Southern California, 1540 Alcazar Street, Los Angeles, 90033 USA; 9 Oncology Safety, Clinical Pharmacology and Safety Sciences, R&D Biopharmaceuticals, AstraZeneca, 1 Francis Crick Avenue, Cambridge, CB2 0RE UK; 10 Division of Systems Medicine, Department of Metabolism, Digestion and Reproduction, Imperial College London, South Kensington Campus, London, SW7 2AZ UK; 11 Cancer Metabolism and Systems Toxicology Group, Division of Cancer, Department of Surgery and Cancer, Imperial College London, Hammersmith Hospital Campus, London, W12 0NN UK; 12 Quantitative Genomics Medicine Laboratories (qGenomics), Esplugues del Llobregat, Barcelona, Catalonia Spain; 13 Department af Environmental Health, Norwegian Institute of Public Health, Lovisenberggt 6, 0456 Oslo, Norway; 14 University Grenoble Alpes, Inserm, CNRS, Team of Environmental Epidemiology Applied to Reproduction and Respiratory Health, IAB, 38000 Grenoble, France; 15 Bradford Institute for Health Research, Bradford Royal Infirmary, Bradford, BD9 6RJ UK; 16 Grupo de Medicina Xenómica, Centro Nacional de Genotipado (CEGEN-PRB3-ISCIII), Universidade de Santiago de Compostela, Praza do Obradoiro s/n, 15782 Santiago de Compostela, Spain; 17 MRC Centre for Environment and Health, School of Public Health, Imperial College London, St. Mary’s Hospital Campus, London, W21PG UK; 18 Department of Social Medicine, School of Medicine, University of Crete, Heraklion, Crete, Greece; 19 Departament de Biomedicina, Institut de Neurociències, Universitat de Barcelona, Barcelona, Spain

**Keywords:** Tobacco smoking, Secondhand smoke, Children, Pregnancy, Omics, Molecular phenotypes, DNA methylation, Transcription, miRNA, Metabolomics

## Abstract

**Background:**

The adverse health effects of early life exposure to tobacco smoking have been widely reported. In spite of this, the underlying molecular mechanisms of in utero and postnatal exposure to tobacco smoke are only partially understood. Here, we aimed to identify multi-layer molecular signatures associated with exposure to tobacco smoke in these two exposure windows.

**Methods:**

We investigated the associations of maternal smoking during pregnancy and childhood secondhand smoke (SHS) exposure with molecular features measured in 1203 European children (mean age 8.1 years) from the Human Early Life Exposome (HELIX) project. Molecular features, covering 4 layers, included blood DNA methylation and gene and miRNA transcription, plasma proteins, and sera and urinary metabolites.

**Results:**

Maternal smoking during pregnancy was associated with DNA methylation changes at 18 loci in child blood. DNA methylation at 5 of these loci was related to expression of the nearby genes. However, the expression of these genes themselves was only weakly associated with maternal smoking. Conversely, childhood SHS was not associated with blood DNA methylation or transcription patterns, but with reduced levels of several serum metabolites and with increased plasma PAI1 (plasminogen activator inhibitor-1), a protein that inhibits fibrinolysis. Some of the in utero and childhood smoking-related molecular marks showed dose-response trends, with stronger effects with higher dose or longer duration of the exposure.

**Conclusion:**

In this first study covering multi-layer molecular features, pregnancy and childhood exposure to tobacco smoke were associated with distinct molecular phenotypes in children. The persistent and dose-dependent changes in the methylome make CpGs good candidates to develop biomarkers of past exposure. Moreover, compared to methylation, the weak association of maternal smoking in pregnancy with gene expression suggests different reversal rates and a methylation-based memory to past exposures. Finally, certain metabolites and protein markers evidenced potential early biological effects of postnatal SHS, such as fibrinolysis.

## Background

The in utero period and the first years of human life are crucial for the development and maturation of organs [1]. Insults during these periods may result in later adverse health consequences, which might persist during the whole lifespan. This is known as the Developmental Origins of Health and Disease (DOHaD) concept [2].

Maternal smoking during pregnancy represents one of the most important avoidable risk factors, and its short- and long-term adverse effects on offspring, including prematurity, lower birth weight, increased risk of asthma and obesity, and impaired neurodevelopment, have been widely reported [3, 4]. In 15 European countries, the prevalence of maternal smoking at any time during pregnancy ranged between 4.2 and 18.9% in 2011–2012 [5]. Secondhand smoke (SHS) is one of the main contributors to the indoor air pollution, with 40% of children exposed worldwide in 2004 [6]. In Europe between 1999 and 2008, among never-smoking adolescents, around 50%, 70%, and 45% were exposed to SHS inside home, outside home, and both, respectively [7]. SHS has been related to increased risk of asthma, lower respiratory infections, and sudden infant death syndrome [4, 6].

The molecular alterations resulting from tobacco smoke exposure are only partially understood. Their study can facilitate the development of biomarkers of exposure that surpass the limitations of existing ones, such as questionnaires and urinary cotinine, which only informs about recent exposure [8, 9]. For instance, the first epigenetic biomarker of maternal smoking allowed the discrimination between exposed and unexposed children with an accuracy > 90% [10]. They may also provide knowledge on the molecular mechanisms that could mediate the effects of tobacco smoking on health. For instance, it has been described that epigenetic deregulation of *JNK2* gene by maternal smoking during pregnancy is associated with impaired lung function in early childhood [11]. Also, methylation levels of maternal smoking-related CpGs in adolescents/adults have been causally linked through Mendelian randomization to inflammatory bowel disease and schizophrenia [12]. Moreover, molecular responses might be more sensitive and earlier markers of a biological effect than clinical outcomes.

In this line, several studies have shown that maternal smoking in pregnancy is associated with altered patterns of DNA methylation at birth, in placenta [13] and in cord blood [11, 14–16]. Interestingly, some of the maternal smoking-related blood loci show persistent dysregulation until childhood [15, 16], adolescence [16, 17], or even adulthood [18]. However, not much is known about the transcriptional consequences of these persistent DNA methylation changes [15]. Furthermore, while the alterations at multiple molecular layers, from epigenetics to metabolomics, have been investigated in relation to current smoking in adults [19–22], there is lack of information about the multi-layer molecular changes associated with in utero exposure or with the exposure to SHS in children. Regarding adult SHS exposure, only DNA methylation candidate studies are available [23].

Here, we aimed to identify multi-layer molecular signatures associated with exposure to tobacco smoke in two early life susceptibility windows, in utero due to maternal smoking and in childhood through exposure to SHS. For this, we used molecular data from 1203 children of the Human Early Life Exposome (HELIX) study, including child blood DNA methylation and transcription, plasma proteins, and sera and urinary metabolites.

## Methods

### Study population

The Human Early Life Exposome (HELIX) study is a collaborative project across 6 established and ongoing longitudinal population-based birth cohort studies in Europe [24]: the Born in Bradford (BiB) study in the UK [25]; the Étude des DÉterminants prÉ et postnatals du dÉveloppement et de la santÉ de l’Enfant (EDEN) study in France [26]; the INfancia y Medio Ambiente (INMA) cohort in Spain [27]; the Kaunas cohort (KANC) in Lithuania [28]; the Norwegian Mother, Father and Child Cohort Study (MoBa) [29]; and the RHEA Mother Child Cohort study in Crete, Greece [30]. Around age 8 years, the HELIX follow-up visit of the offspring took place using harmonized questionnaires and sampling protocols (*n* = 1301). HELIX children with complete data on prenatal and postnatal variables of exposure to tobacco smoking and with data on at least one omics platform were selected for the present study (*n* = 1203). Years of enrollment, years of HELIX visit, and years of smoking prohibition in each cohort are shown in Additional file 1: Table S1.

### Biological samples

At the HELIX follow-up visit, child peripheral blood was collected and processed into a variety of sample matrices: buffy coat, serum, plasma, and whole blood. Median fasting time was 3.5 h (SD = 1.1 h). Two spot urine samples (one before bedtime and one first morning void) were collected at the participants’ home and transported at 4 °C to the cohort center, where they were combined at equal volumes to create a daily urine pool. In the HELIX follow-up, the daily urine pool was available for 92.90% of the children, while the first morning and bedtime urines were available for 3.14% and 3.96% of the children, respectively. All sample types were stored at − 80 °C until processed.

### Exposure to tobacco smoking

#### Definitions of exposure to tobacco smoking in pregnancy

Maternal smoking habits during pregnancy were obtained from existing data collected in each cohort using non-harmonized questionnaires administered to the mothers in at least the first and third trimester of pregnancy. Two variables for active maternal smoking during pregnancy were generated: (i) any maternal smoking during pregnancy if the mother had smoked at any time during pregnancy (“yes/no”), and (ii) sustained maternal smoking, if the mother had smoked, at least, in the 1st and in the 3rd trimester (“yes/no”). The mean number of cigarettes smoked per day during pregnancy was estimated averaging the mean number of cigarettes per day in the first and third trimesters.

Maternal exposure to SHS in pregnancy (mother-SHS: “yes/no”) was assessed through questionnaire and was slightly different between cohorts: (i) exposure at home, work, or leisure places (INMA); (ii) exposure at home or work (MoBa, RHEA, BiB); (iii) exposure without specifying location (EDEN); and (iv) partner smoking (KANC).

We combined all previous definitions to create a variable that captured both dose and duration of exposure to smoking during pregnancy with the following categories: “unexposed,” “SHS,” “non-sustained smoker,” “sustained smoker at low dose (≤ 9 cigarettes per day),” and “sustained smoker at high dose (> 9 cigarettes per day).”

#### Definitions of childhood exposure to secondhand smoke

Childhood exposure to secondhand smoke (SHS) was assessed through a harmonized questionnaire administered to the parents as part of the HELIX project. The questionnaire included questions about (i) smoking at home by the mother, mother’s partner, or other people, and (ii) attendance to indoor places where people smoke. From this information, we created a variable of SHS with four levels: “unexposed,” “exposed to SHS only outside home,” “exposed to SHS only inside home,” and “exposed to SHS inside and outside home.” This variable was reclassified in larger groups to assess global exposure to SHS (global-SHS: “yes/no”; home-SHS: “yes/no”).

Urinary cotinine levels were measured in the children using the Immulite2000 Nicotine Metabolite (Cotinine) 600 Test on an Immulite 2000 XPi from Siemens Healthineers at Fürst Medisinsk Laboratorium in Norway for which the LOD was 3.03 μg/L. Due to low concentrations in children, a categorical variable was created (cotinine: “detected/undetected”).

#### Correlation between exposure variables

The correlation among the proportion of exposed children using different smoking definitions and different windows of exposure was calculated using the tetrachoric correlation test, with the psych R package [31]. The tetrachoric correlation estimates what the correlation would be if measured on a continuous scale.

### Molecular phenotypes

Detailed information on molecular characterization of the 4 molecular layers (6 omics datasets) can be found in Additional file 2: Additional Methods [32–49].

#### Blood DNA methylation

Briefly, methylation was measured in DNA extracted from buffy coat (EDTA tube) using the Infinium HumanMethylation450 beadchip (Illumina, USA) at the Spanish National Genotyping Center (CeGen, Spain). Samples were randomized and balanced by sex and cohort within each batch. Samples with low call rate (< 98%) were excluded. Also, probes with low call rate (< 95%) [32], probes in sexual chromosomes, cross-hybridizing probes, and probes containing single nucleotide polymorphisms (SNPs) were filtered out [34]. Methylation levels were normalized using the functional normalization method with prior background correction with Noob [33], and slide batch effect was controlled with the ComBat method [36]. Beta values, going from 0 (un-methylated) to 1 (fully methylated), were used in the analyses. CpGs were annotated with the IlluminaHumanMethylation450kanno.ilmn12.hg19 R package [35]. Blood methylation quantitative trait loci (mQTL) were retrieved from mQTLdb (http://www.mqtldb.org/) (database: MatrixEQTL; timepoint: childhood; distance: 1 Mb).

#### Blood gene expression

RNA was extracted from whole blood collected in Tempus tubes. Gene expression was assessed using the GeneChip® Human Transcriptome Array 2.0 (HTA 2.0) (Affymetrix, USA) at the University of Santiago de Compostela (USC, Spain). Samples were randomized and balanced by sex and cohort within each batch. Data was normalized at the gene level with the GCCN (SST-RMA) algorithm, and batch effects and blood cell type composition were controlled with two surrogate variable analysis (SVA) methods, isva [38] and SmartSVA [39], during the differential expression analyses. Gene expression values were log2 transformed, and annotation of transcript clusters (TCs) to genes was done with the Affymetrix Expression Console software using the HTA-2_0 Transcript Cluster Annotations Release na36 (hg19).

#### Blood miRNA expression

miRNA expression was quantified using the SurePrint Human miRNA Microarray rel.21 (Agilent Technologies, USA) [42], at the Genomics Core Facility at the Centre for Genomic Regulation (CRG, Spain). Samples were randomized and balanced by sex and cohort within each batch. miRNA expression levels were normalized with the least variant set (LVS) method [43] with background correction with the Normexp method [44]. Normalized miRNA levels were log2 transformed and annotated using a combination of information from Agilent annotation (“Annotation_7056”) and miRbase v21 (GRCh38 and mapped back to hg19) released in January 2017. Additional control of batch effect and blood cell composition during the differential expression analyses was done with the SVA standard method [45].

#### Plasma proteins

Plasma protein levels were assessed using the antibody-based multiplexed platform from Luminex at the Proteomics Unit (Centre for Genomic Regulation (CRG)/University Pompeu Fabra (UPF), Spain), using 3 commercial kits (Thermo Fisher Scientific, USA): Cytokines 30-plex (catalog number (CN): LHC6003M), Apoliprotein 5-plex (CN: LHP0001M), and Adipokine 15-plex (CN: LHC0017M) (Additional file 1: Table S2). All samples were randomized and blocked by cohort. Raw intensities were converted to nanograms per milliliter (5-plex kit) and to picograms per milliliter (15- and 30-plex kits) using 8-point calibration curves added in each plate. Only 36 proteins out of 43 with > 30% of measurements in the linear range of quantification were kept for the analysis. Protein levels were log2 transformed, and plate batch effect was corrected. Values below the lower limit of quantification (LOQ1) and above the upper limit of quantification (LOQ2) were imputed using the truncdist R package [46].

#### Serum metabolites

Serum metabolites were quantified using the targeted metabolomics Absolute-*IDQ*^TM^ p180 Kit (Biocrates Life Sciences AG, USA). Serum metabolic profiles were acquired following the manufacturer’s protocol using LC-MS/MS on a Sciex QTrap 6500 equipped with an Agilent 1100 series HPLC (Agilent Technologies, USA), at Imperial College London (ICL, UK); a full description of the HELIX metabolomics methods and data can be found elsewhere [47]. Samples were fully randomized. Metabolites were quantified (mM) following the manufacturer’s protocol (appendix), and then log2 transformed. Metabolite exclusion was based on a metabolite variable meeting two conditions: (1) CV of over 30% and (2) over 30% of the data are below LOD. Eleven out of the 188 serum metabolites detected were excluded as a result, leaving 177 serum metabolites to be used for further statistical analysis. The mean coefficient of variation across the 177 LC-MS/MS detected serum metabolites was 16%. Analytical performance was in line with expectations from and inter-laboratory ring trial of this platform [48].

#### Urinary metabolites

Urinary metabolic profiles were analyzed on a 14.1-T (600 MHz ^1^H) NMR spectrometer (Bruker, USA) at Imperial College London (ICL, UK) [47]. Urine samples were fully randomized. In addition, 60 identical study quality control urine samples were included at regular intervals during the runs. While data acquisitions were untargeted, data processing workflow followed a targeted strategy to identify and quantify the 44 most abundant metabolites in urine [47, 49]. Sample concentration of a given metabolite was estimated from the signal of the internal standard trimethylsilylpropanoic (TSP). Data was normalized using median fold change normalization method which takes into account the distribution of relative levels from all 44 metabolites compared to the reference sample in determining the most probable dilution factor. Twenty-six metabolites were absolutely quantified, and 18 semi-quantified. Concentration levels were expressed as log2, and before that, we used an off-set of ½ the minimal value for each metabolite.

### Statistical analyses

#### Analysis steps

The steps to analyze the HELIX data were as follows: (1) We systematically analyzed the association between period-specific exposure to tobacco smoke and child molecular marks from the different omics layers. (2) For statistically significant molecular marks, we tested the effect of dose and duration of the exposure. (3) For CpGs only, we searched for cis expression quantitative trait methylation (eQTM). (4) We contrasted our findings with the previous literature, either through direct comparison or through enrichment analyses. (5) We conducted sensitivity analyses to test the robustness of the findings to exposure definitions, period-specific effects, and ancestry.

#### Association between exposure to smoking and molecular phenotypes

To test the relationship between exposure to tobacco smoke versus child molecular marks, we fitted linear regressions between each tobacco smoking variable (predictor) and each molecular mark (outcome) adjusting for covariates, using limma [44] within the implementation of omicRexposome R package [41]. Children unexposed to tobacco smoke, in each definition and period, were considered the reference group. Since we wanted to assess period-specific smoking effects, models were mutually adjusted: any and sustained maternal smoking in pregnancy were adjusted for childhood global-SHS, while childhood global-SHS and urinary cotinine detection were adjusted for sustained maternal smoking in pregnancy.

Other covariates were selected through directed acyclic graphs (DAGs) with the DAGitty tool [50] (Additional file 3: Fig. S1; and Additional file 3: Fig. S2). The following variables were selected for both periods: (i) cohort, (ii) self-reported ancestry (European ancestry, Pakistani or Asian, and others), (iii) maternal age, and (iv) self-reported maternal education (low (primary school), medium (secondary school), and high (university degree or higher)). In addition, pregnancy models were adjusted for maternal obesity status, and childhood models for child body mass index (zBMI). Maternal BMI was calculated from pre- or early-pregnancy weight and height and divided in four categories derived from World Health Organization (WHO) definitions. Child zBMI is a sex and age *z*-score calculated according to WHO reference curves [51, 52]. Single imputation of missing data for covariates was done using a chained equations method [53] with the mice R package [54]. The proportion of missing data in the selected covariates was minimal: 0.17% in maternal age, 0.75% in maternal BMI, and 2.16% in maternal education. Apart from covariates resulting from DAG diagrams, models were also adjusted for sex and child age, as well as for specific covariates in models of each of the omics. Methylation models were adjusted for blood cell type composition (CD4+ and CD8+ T cells, natural killer cells (NK), monocytes, granulocytes, and B cells), which was estimated from raw methylation data [55, 56]. Models of plasma proteins and serum metabolites were adjusted for time to last meal and hour of blood collection, and models for urine were adjusted for sample type (morning, night, or the pool of both). Serum and urinary metabolites were additionally corrected for technical batch. Technical batch effects of the other omics datasets were eliminated during the quality control process, as described in previous sections.

Multiple-testing correction was performed within each molecular layer following different approaches. For omics with more than 1000 features (methylation, gene expression, and miRNAs), we used false discovery rate (FDR)–Benjamini-Hochberg correction [57]. For the remaining omics, we divided the nominal *p* value by the effective number of tests [58] (proteins—18.4, *p* value = 2.72E−03; serum metabolites—60.3, *p* value = 8.29E−04; urinary metabolites—33.3, *p* value = 1.50E−03).

The effect of smoking on DNA methylation is reported as a difference in methylation levels between exposed and unexposed children, while for the other omics, the effect is reported as a log2 fold change (log2FC). To examine whether different definitions of exposure (i.e., any vs. sustained) or different adjustments yielded increased or decreased magnitudes of association, we calculated the percent change in the coefficients (*β*) between the two models (i.e., sign(max(*β*_Sust.;_*β*_Any_) × (*β*_Sust._ − *β*_Any_)/|*β*_Any_| × 100)).

#### Dose and duration of the effect

To study the effect of dose and duration during pregnancy, we fitted linear regressions between the levels of the molecular biomarker (outcome) and the exposure to tobacco smoking (predictor) defined in the following categories: “unexposed,” “SHS,” “non-sustained smoker,” “sustained smoker at low dose (≤ 9 cigarettes per day),” and “sustained smoker at high dose (> 9 cigarettes per day),” where “unexposed” was the reference category. Similarly, for the childhood exposure, we fitted linear regressions between the levels of the molecular biomarker (outcome) versus the exposure to tobacco smoking (predictor) defined in the following categories: “unexposed,” “exposed to SHS only outside home,” “exposed to SHS only inside home,” and “exposed to SHS inside and outside home,” where “unexposed” was considered the reference category.

#### Expression quantitative trait methylation

For CpGs significantly associated with exposure to tobacco smoke, we searched for cis eQTMs using methylation and gene expression data from 874 HELIX children. Cis effects were defined in a window of 1 Mb from the transcription start site (TSS) of each TC (each gene). The association between DNA methylation and gene expression levels was assessed via 1420 linear regressions. Models were adjusted for cohort, child’s age, sex, zBMI, ancestry, and blood cell type composition. To control for gene expression batch effect, we estimated surrogate variables (SVs) with the SVA R package [45], protecting covariates (cohort, child’s age, sex, zBMI, ancestry, and blood cell type composition), and the effect of these SVs was eliminated from the gene expression matrix by calculating the residuals.

#### Direct comparison with the literature and enrichment analyses

We compared findings in our study with previous literature on the molecular effects of exposure to tobacco smoke in pregnancy (DNA methylation [15]) and of own smoking in adults (DNA methylation [21], gene expression [20], miRNA expression [22], serum metabolites [19]). When possible, we did a detailed comparison with previous findings; for genome-wide omics without significant marks in our study, we conducted enrichment analyses. To explore enrichment of our results for molecular marks (CpGs/genes) identified previously for current smoking in adults, we tested whether the distribution of *p* values at these marks in our data deviates from a null distribution using one-sided Kolmogorov-Smirnov tests, and compared the direction of effect estimate to what was reported previously.

#### Sensitivity analyses

On the molecular marks that survived multiple-testing correction, we performed sensitivity analyses to test the robustness of results under the following scenarios: (i) for models of any and sustained maternal smoking in pregnancy, we tested the effect of adjusting for home-SHS, instead of global-SHS; (ii) we compared main models, which are mutually adjusted for the two exposure periods (i.e., maternal smoking in pregnancy adjusted for childhood SHS), versus unadjusted models (i.e., maternal smoking in pregnancy without adjustment for childhood SHS); and (iii) finally, we restricted the analysis to children of European ancestry (*N* = 1083).

All the analyses were done in R environment. The R packages MultiDataSet [59], rexposome, and omicRexposome [41] were used to manage and analyze the omics and exposure data. ggplot2 [60], qqman [61], calibrate [62], sjPlot [63], OmicCircos [64], and coMET [65] R packages were used to visualize the results.

## Results

### Study population

The study included 1203 children, aged 6 to 11 years, from the Human Early Life Exposome (HELIX) project that had complete information on pregnancy and childhood exposure to tobacco smoking and data on at least one omics platform [24]. These children were from longitudinal cohorts in 6 European countries, 90.1% were of European ancestry, 54.5% were males, and 51.5% were born from highly educated mothers (Table 1). Molecular features measured at an average age of 8.1 years included blood DNA methylation, blood gene and miRNA expression, plasma proteins, and sera and urinary metabolites (Table 2). For 93% of the children, omics data from at least 4 platforms was available (Table 1).

**Table 1:** Description of the HELIX study participants (*n* = 1203)

Variable	*n* (%) or mean (SD)
Cohort	
BiB (UK)	176 (14.6)
EDEN (France)	171 (14.2)
INMA (Spain)	215 (17.9)
KANC (Lithuania)	189 (15.7)
MoBa (Norway)	255 (21.2)
RHEA (Greece)	197 (16.4)
Child sex	
Female	547 (45.5)
Male	656 (54.5)
Child age (years)	8.1 (1.6)
Child zBMI*	0.4 (1.2)
Child ancestry	
European	1083 (90.1)
Pakistani or Asian	93 (7.7)
Others	27 (2.2)
Maternal age (years)	30.8 (4.8)
Maternal BMI categories	
< 18.5	46 (3.8)
18.5–24.9	691 (57.4)
25–29.9	295 (24.5)
≥ 30	171 (14.2)
Maternal education	
Low	162 (13.5)
Medium	421 (35.0)
High	620 (51.5)
Pregnancy: any maternal smoking in pregnancy	
No	1027 (85.4)
Yes	176 (14.6)
Pregnancy: sustained maternal smoking in pregnancy	
No	1027 (90.0)
Yes	114 (10.0)**
Childhood: global-SHS	
No	777 (64.6)
Yes	426 (35.4)
Childhood: urinary cotinine	
Not detected (no)	993 (82.5)
Detected (yes)	210 (17.5)
Number of omics datasets	
6	834 (69.3)
4 or 5	282 (23.4)
2 or 3	25 (2.2)
1	62 (5.10)

*Sex and age *z*-score calculated according to WHO reference curves

**9.5% out of the 1203 children

**Table 2:** Sample size and number of features included in each omics dataset

Omics dataset	Number of samples			Number of features	
	Initial	After QC	With data on smoking	Initial	After QC
Blood DNA methylation	1200	1192	1105	485,512	386,518
Blood gene expression	1176	1158	958	64,568	58,254
Blood miRNA gene expression	961	955	895	2549	1117
Plasma proteins	1212	1188	1103	43	36
Serum metabolites	1209	1208	1128	188	177
Urine metabolites	1212	1211	1131	64,000	44

*QC* quality control

### Exposure to tobacco smoking

**Fig. 1:**
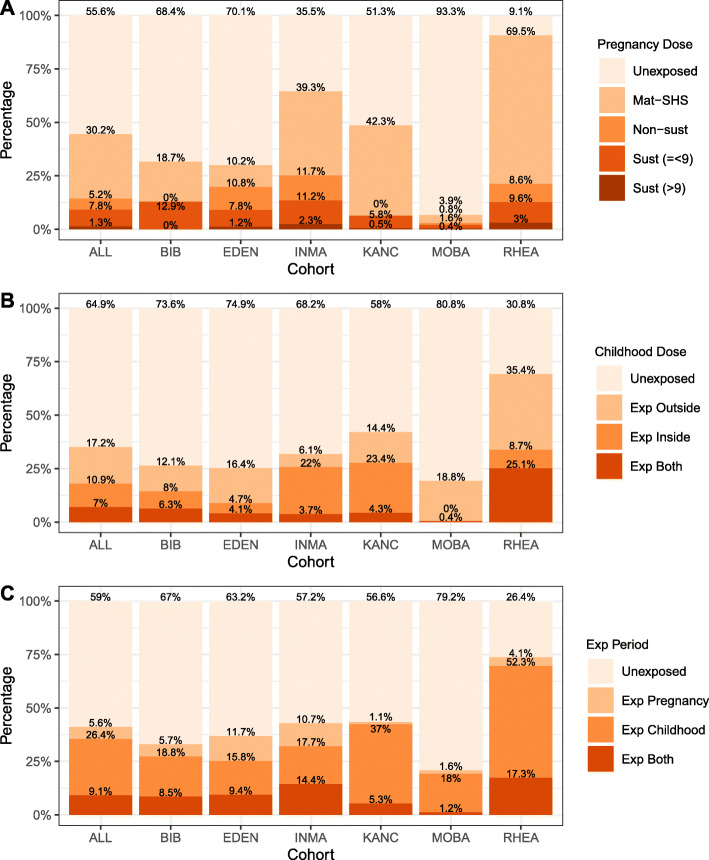
Percentage of children exposed to different dose and/or duration of tobacco smoking in all children and by cohort: in pregnancy (**a**), in childhood (**b**), and in pregnancy and childhood combined (**c**). Mat-SHS, mothers exposed to SHS; Non-sust, non-sustained smoker mothers; Sust (=<9), sustained smoker mothers at low dose—less than or equal to 9 cigarettes per day; Sust (>9), sustained smoker mothers at high dose—more than 9 cigarettes per day. Other categories are self-explanatory

Out of the 1203 mothers, 14.6% reported having smoked at some point during pregnancy (any smoking), and 9.5% reported having smoked throughout whole pregnancy (sustained smokers) (Table 1). When considering information on dose and duration during pregnancy (*n* = 1193), 55.6% of the mothers were unexposed, 30.2% were exposed to SHS, 5.2% were non-sustained smokers, 7.8% were sustained smokers at low dose (≤ 9 cigarettes per day), and 1.3% were sustained heavy smokers (Fig. 1a).

The frequency of children exposed to global-SHS, meaning exposure at home or in other places, was 35.4% (Table 1): 17.2% were exposed only outside home, 10.9% only at home, and 7% in both places (0.4% did not had enough information to be classified in any of these categories) (Fig. 1b). Moreover, 17.5% of the children had urinary cotinine levels over the limit of detection (LOD) (Table 1). The correlations of cotinine measures with global-SHS and home-SHS were 0.7 and 0.8, respectively (Table 3).

**Table 3:** Correlation among variables of pregnancy and childhood exposure to tobacco smoking

		Pregnancy exposure		Childhood exposure		
		Any maternal smoking in pregnancy	Sustained maternal smoking in pregnancy	Global-SHS	Home-SHS	Urinary cotinine
Pregnancy exposure	Any maternal smoking in pregnancy	1				
	Sustained maternal smoking in pregnancy	1*	1			
Childhood exposure	Global-SHS	0.4	0.4	1		
	Home-SHS	0.6	0.6	1**	1	
	Urinary cotinine	0.7	0.7	0.7	0.8	1

*Complete cases were used to test the correlation between any and sustained maternal smoking in pregnancy, and since non-sustained smokers are excluded from sustained maternal smoking in pregnancy, both variables are virtually the same in this comparison

**By definition, all children exposed at home to SHS are also exposed in global-SHS (*n* = 214); however, unexposed children according to global-SHS are both exposed (*n* = 214) and unexposed (*n* = 777) at home

The correlations between in utero and childhood exposures are shown in Table 3. The highest correlation of maternal smoking in pregnancy was with child cotinine levels (0.7), then with home-SHS (0.6), and finally with global-SHS (0.4). Considering a combination of any maternal smoking in pregnancy and global-SHS exposure during childhood, 59% of the children were not exposed to any smoking, 5.6% were exposed only in pregnancy, 26.4% only after birth, and 9.1% in both periods (Fig. 1c).

The proportion of exposed children was highly dependent on the cohort (Fig. 1, Additional file 3: Fig. S3). Southern European cohorts (RHEA-Greece, INMA-Spain, and EDEN-France) had the highest percentage of smoking mothers, and maternal or child exposure to SHS was highest in RHEA, in INMA, and also in KAUNAS-Lithuania. In MoBa-Norway, only 20.8% of the children were exposed to prenatal or childhood tobacco smoke, while in RHEA, this percentage rose to 73.6%.

### Association between maternal smoking in pregnancy and child DNA methylation

#### Screening and comparison with the literature

**Fig. 2:**
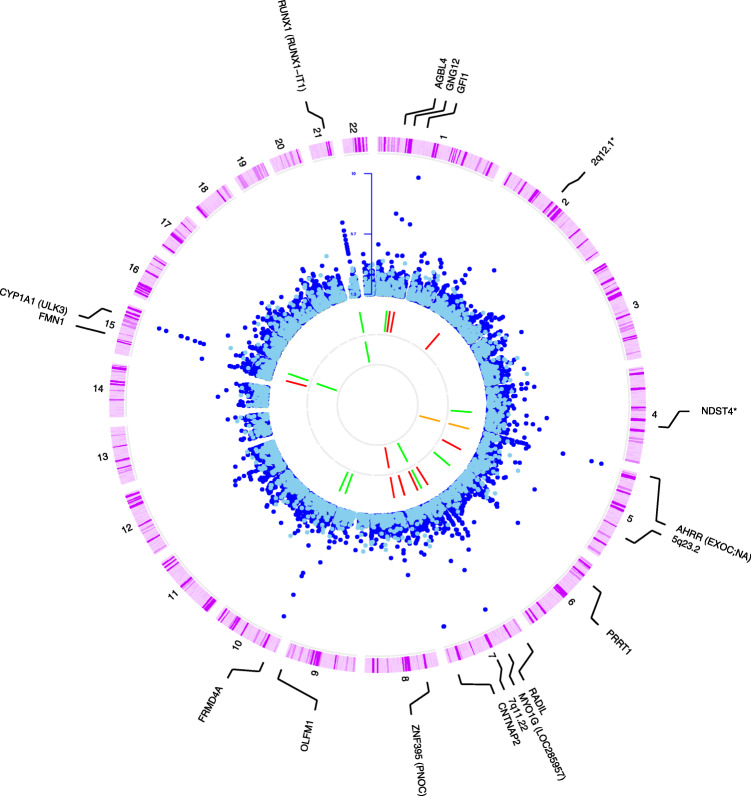
Circus plot showing the association between sustained maternal smoking in pregnancy and child blood DNA methylation and transcription along the chromosomes (outer circle). Second circus shows the statistical significance (−log10(*p* value)) for DNA methylation (dark blue) and transcription (light blue). Only the 18 loci significant at 5% FDR in the methylation analysis are annotated. Next circus shows the direction of the association of the CpGs in these 18 loci with maternal smoking in pregnancy (green, positive; red, inverse; and orange, loci with CpGs associated in both directions). The inner circus shows the 5 loci for which cis eQTMs at 5% FDR were identified (green, positive, meaning higher DNA methylation–higher gene expression; red, inverse; and orange, both). Genes annotated in parenthesis are significant eQTM genes, and none of them corresponds to the closest gene to the CpG site. Loci annotated with an asterisk are those surviving multiple-testing correction only in the any maternal smoking in pregnancy models. To gain graphical resolution, only associations with *p* value < 0.05 are shown, and *p* values < 1E−10 are truncated to 1E−10

After controlling for childhood global-SHS, any and sustained maternal smoking in pregnancy were associated with altered child blood DNA methylation. Lambda inflation factors ranged from 0.951 to 1.003 (Additional file 3: Fig. S4). At 5% false discovery rate (FDR), a total of 41 unique CpGs were differently methylated, when comparing any or sustained maternal smoking in pregnancy to non-smoking: 24 were associated with both smoking definitions, 3 with any, and 14 with sustained, although all of them were at least nominally significant in both models (Additional file 1: Table S3). These 41 CpGs were located in 18 loci, defined as regions of < 2 Mb, and were distributed along the genome (Fig. 2). Around 30% of the CpG sites were hypo-methylated (lower methylation in exposed children) (Additional file 3: Fig. S4), and CpGs located in the same locus were affected in the same direction, except for the *AHRR* locus (Fig. 2). Differential DNA methylation at 17 out of the 18 loci had previously been reported in relation to sustained maternal smoking in pregnancy in cord blood [15] (Additional file 1: Table S3). Moreover, persistent effects until childhood were described for 16 of them [15]. The unique locus not previously related to maternal smoking in pregnancy was *FMN1* (*Formin 1*), but other CpGs in that locus were found differently methylated in current smokers in the opposite direction [21]. Thirteen out of the 18 loci (27 out of the 41 CpGs) had at least one genetic variant (mQTL), in cis and/or trans, associated with methylation levels (Additional file 1: Table S3).

#### Effects of dose and duration

**Fig. 3:**
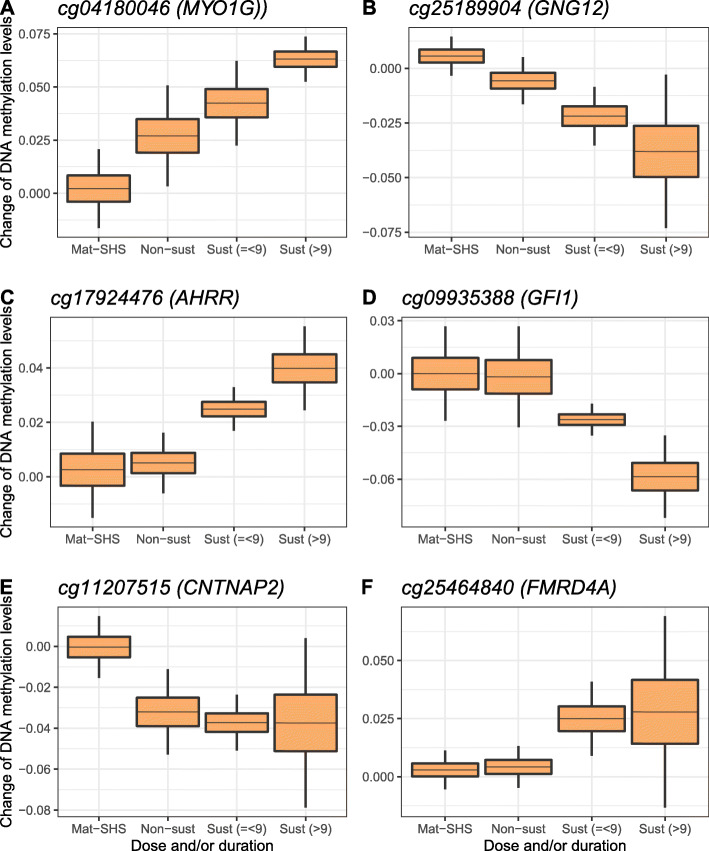
Box plots showing the change of child blood DNA methylation compared to unexposed mothers (*y*-axis) by categories of dose and/or duration of exposure to tobacco smoking in pregnancy (*x*-axis), adjusted for global-SHS. Horizontal line in the middle of the boxes shows the mean difference in DNA methylation with respect to the reference category of unexposed mothers. Boxes represent the DNA methylation change ± standard error (SE), and vertical lines indicate extreme changes defined as ± 3 × SE. Each graph shows an illustrative pattern: **a** tendency of increased methylation with increased dose and/or duration, **b** tendency of decreased methylation, **c** tendency of increased methylation only in sustained maternal smoking in pregnancy, **d** tendency of decreased methylation only in sustained maternal smoking in pregnancy, **e** saturated pattern in non-sustained smokers, and **f** saturated pattern in sustained maternal smoking in pregnancy. The rest of the 41 CpGs can be found in Additional file 4 (Fig. S6). Mat-SHS, mothers exposed to SHS; Non-sust, non-sustained smoker mothers; Sust (=<9), sustained smoker mothers at low dose—less than or equal to 9 cigarettes per day; Sust (> 9), Sustained smoker mothers at high dose—more than 9 cigarettes per day. Other categories are self-explanatory

As expected, stronger effects were observed for sustained maternal smoking in pregnancy compared with any maternal smoking in pregnancy in 39 out of the 41 CpGs (Additional file 3: Fig. S5). The mean absolute percentage change of methylation from any to sustained maternal smoking in pregnancy models was 32.8% (Additional file 1: Table S3). The effect of dose and/or duration of maternal smoking in pregnancy on DNA methylation was investigated. At visual inspection, we identified 4 different illustrative patterns: (i) CpGs exhibiting an increased or decreased DNA methylation tendency with increasing maternal smoking in pregnancy dose and/or duration (Fig. 3a, b); (ii) CpGs with an increased or decreased DNA methylation tendency only with increasing dose of sustained maternal smoking in pregnancy, but without response to any maternal smoking in pregnancy (Fig. 3c, d); (iii) CpGs with a saturated pattern at any maternal smoking in pregnancy (Fig. 3e); and (iv) CpGs with a saturated pattern at sustained maternal smoking in pregnancy (Fig. 3f). The plots for all 41 CpGs are shown in Additional file 4: Fig. S6 (10 showing a linear trend, 4 with a dose response in sustained smokers, 3 saturated with any smoking, and 8 saturated with sustained smoking). Some CpGs did not show a clear pattern, and others located at the same locus showed different patterns. Maternal exposure to SHS was only nominally associated (*p* value < 0.05) with 2 of these 41 CpGs (cg11902777 and cg17454592).

#### Search for gene expression quantitative trait methylation

We, then, examined whether the 41 CpGs might be eQTMs. A total of 480 unique transcript clusters (TCs, equivalent to known or putative genes) with their transcription start site (TSS) within ± 500 kb of the 41 CpGs were identified. At 5% FDR, 15 methylation to expression relationships were found, which included 12 unique CpGs in 5 loci and 7 unique TCs (Fig. 2, Additional file 1: Table S4). All eQTMs, except cg21161138 (*AHRR*)-TC05002792.hg.1, occurred between CpGs and genes located at < 160 kb (Additional file 3: Fig. S7). None of the eQTMs genes corresponded to the most proximal gene to the CpG site. Two out of the 15 eQTM relationships were inverse, meaning higher methylation-lower gene expression, while the others were positive. The inverse associations were between *PNOC* and a CpG (cg17199018) located downstream the gene, and between *EXOC3* and a CpG (cg11902777) located upstream.

**Fig. 4:**
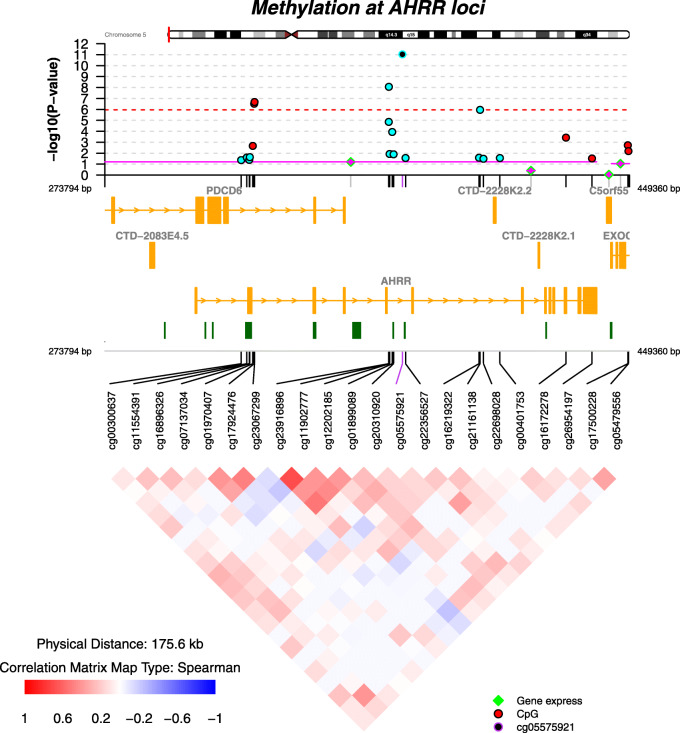
Regional plot of the *AHRR* locus (50 kb upstream and downstream of the 5 CpG sites associated with any maternal smoking in pregnancy). The *y*-axis of the top panel shows the −log (10) *p* value of the associations between any maternal smoking in pregnancy and methylation levels at CpG sites (circles) and gene expression levels (lines). Only CpGs nominally associated (*p* value < 0.05) are shown, hyper-methylated in red and hypo-methylated in blue. The top CpG, cg05575921, is shown in purple. Five of the CpGs survived multiple-testing correction (dashed red line): 2 hyper-methylated in intron 1 (cg17924476 and cg23067299), and 3 hypo-methylated at other introns (cg11902777, cg05575921, and cg21161138). The correlation of methylation levels among CpGs is shown at the bottom panel. The middle panel shows the annotation of genes (yellow) and CpG islands (green). The expression of none of the genes in the locus for which there were probes in the gene expression array was associated with any maternal smoking in pregnancy (*p* value < 0.05). All of them showed negative coefficients of the association (indicated as pink lines). List of TCs and gene annotation: TC05001094.hg.1 annotated to *EXOC3-AS1*, TC05000006.hg.1 annotated to *EXOC3*, TC05000005.hg.1 annotated to both *AHRR* and *PDCD6*, and TC05002795.hg.1 not annotated

With a nominal *p* value < 0.05, 65 other eQTMs were detected, in total, involving 33 unique CpGs in 14 out of the 18 loci (Additional file 1: Table S4). Only the methylation levels of CpGs at the gene body of *GFI1* and *AHRR* were associated with the expression of the same genes. *AHRR*, associated with maternal smoking in pregnancy [15] and current smoking in adults [21], is an interesting example. Five CpGs in the *AHRR* locus were associated with maternal smoking in pregnancy: 2 hyper-methylated located at intron 1 (cg17924476, cg23067299), and 3 hypo-methylated at other introns (cg11902777, cg05575921, cg21161138) (Fig. 4). Hyper-methylated CpGs in relation to maternal smoking in pregnancy were positive eQTMs for *AHRR*, *PDCD6*, and *EXOC3* genes, while hypo-methylated CpGs were inverse eQTMs for the same genes (in both cases implying higher expression of the genes).

### Association between maternal smoking in pregnancy and other child molecular phenotypes

Besides child DNA methylation, we also screened the association between maternal smoking in pregnancy and the other molecular layers: gene and miRNA transcription, plasma proteins, and serum and urinary metabolites.

After multiple-testing correction, only 2 associations between maternal smoking in pregnancy and urinary metabolites were detected. Urinary alanine and lactate were increased in children of mothers classified as sustained smokers during pregnancy compared to children of non-smoking mothers (effect = 0.189 and 0.174, *p* value = 3.93E−04 and 5.19E−04, respectively) (Additional file 1: Table S5).

No associations were found between maternal smoking in pregnancy and child serum metabolites or blood gene/miRNA expression. However, given the effect of maternal smoking on child methylation and the eQTM analyses, we took a closer look at gene expression (Fig. 2). Top 10 associations can be seen in Additional file 1: Table S6, and among the 15 eQTM genes, only *EXOC3* was nominally downregulated in children of smoker mothers (*p* value < 0.01) (Additional file 1: Table S7).

### Association between childhood exposure to SHS and child molecular phenotypes

In postnatal life, childhood SHS exposure was related to child plasma protein and serum metabolite levels, but not to any other of the molecular layers (blood DNA methylation, gene/miRNA transcription, or urinary metabolites).

**Fig. 5:**
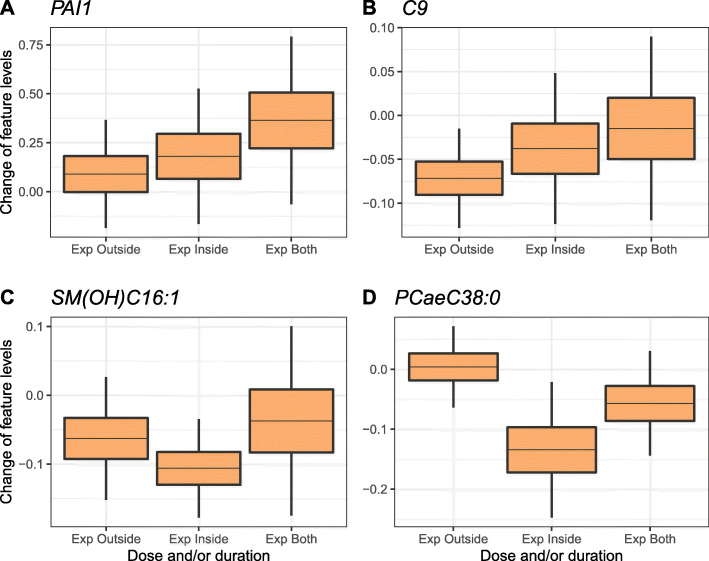
Box plots showing the change of child protein/metabolite levels compared to unexposed mothers (*y*-axis) by categories of dose and/or duration of exposure to SHS (*x*-axis), adjusted for sustained maternal smoking in pregnancy. Horizontal line in the middle of the boxes shows the mean difference in protein/metabolite levels with respect to the reference category of unexposed mothers. Boxes represent the protein/metabolite change ± standard error (SE), and vertical lines indicate extreme changes defined as ± 3 × SE. **b** Plasma PAI1 levels, **b** serum C9, **c** serum SM (OH) C16:1, and **d** serum PC ae C38:0

In particular, higher levels of PAI1 (plasminogen activator inhibitor-1) protein were found in children with urinary cotinine levels above the LOD compared to those below the LOD (effect = 0.379, *p* value = 1.66E−04) (Additional file 1: Table S8). PAI1 levels increased with increased frequency of exposure, with children exposed both inside and outside home having the highest PAI1 levels (Fig. 5). This association was attenuated when testing global-SHS instead of urinary cotinine levels.

On the other hand, children classified as exposed to SHS under different definitions had lower levels of several serum metabolites compared to those classified as not exposed [global-SHS: sphingomyelin (OH) C16:1 (effect = − 0.073, *p* value = 7.97E−05), carnitine C9 (effect = − 0.052, *p* value = 8.03E−04), and cotinine: PC ae C38.0 (effect = − 0.110, *p* value = 7.88E−04)] (Additional file 1: Table S8). We only detected a clear pattern of a dose-effect response for carnitine C9 (Fig. 5).

### Comparison and enrichment for signals identified in current smokers

We, then, compared our results of exposure to tobacco smoke in children, both in pregnancy and in postnatal life, with the molecular marks identified for current smoking in adults. For serum metabolites [19] and miRNAs [22], molecular layers with a limited number of marks assessed in the omics platforms, we did a direct comparison of the findings. For genome-wide omics, we analyzed whether our findings showed any enrichment for the signals identified in studies of current smoking and DNA methylation (*N* = 16,223 CpGs at 5% FDR) [21], and gene expression (*N* = 1270 genes at 10% FDR) [20].

As in current smokers [19], children exposed to postnatal SHS had lower levels (*p* value < 0.05) of diacyl (aa)-phosphatidylcholines (PC aa C36:0, PC aa C38:0), acyl-alkyl (ae)-phosphatidylcholines (PC ae C38:0, PC ae C38:6, PC ae C40:6), and sphingomyelin (OH) C22:2 (Additional file 1: Table S9) compared to non-exposed children. None of the amino acids reported to be increased in current smokers were affected in SHS-exposed children. In contrast to this, maternal smoking in pregnancy had no effect on serum metabolites related to current smoking (Additional file 1: Table S10).

None of the miRNAs for current smoking were nominally significant in our study, either with exposure pregnancy or in childhood (Additional file 1: Table S11 and Table S12). Similarly, no enrichment for current smoking sensitive genes was observed among our results of exposure during pregnancy (enrichment *p* values for any and sustained maternal smoking, 0.903 and 0.842, Additional file 3: Fig. S8) or our results of exposure to childhood SHS (enrichment *p* values for global-SHS and child cotinine, 0.579 and 0.746, Additional file 3: Fig. S9).

Regarding DNA methylation, we also found that our results for childhood SHS were not enriched for CpGs associated with current smoking (enrichment *p* values for global-SHS and child cotinine, 0.034 and 0.998) (Additional file 3: Fig. S10). In contrast, we observed enrichment among our list of CpGs associated with maternal smoking in pregnancy (enrichment *p* values for any and sustained maternal smoking in pregnancy, 2.834E−07 and < 2.2E−16, respectively) (Additional file 3: Fig. S11). In particular, the DNA methylation at 1279 of the 16,223 current smoking sensitive CpGs [21] was nominally significant in children of sustained smoker mothers, 73.3% of them with consistent direction of the effect, and with a lambda inflation factor of 1.16.

### Sensitivity analyses for pregnancy and childhood exposure to smoking

We conducted further sensitivity analyses for the molecular marks that survived multiple-testing correction. First, we ran additional pregnancy models adjusting for home-SHS instead of global-SHS. In general, the strength of the association was reduced, but for DNA methylation, the change in effect size between models was low (mean absolute percentage change < 5%) (Additional file 3: Fig. S12, Additional file 1: Table S13-S14).

Second, we compared adjusted and unadjusted models for exposure to smoking in the reciprocal period. For maternal smoking in pregnancy, mainly associated with DNA methylation levels, results were practically the same between models, again, with mean absolute percentage change < 5% (Additional file 3: Fig. S13, Additional file 1: Table S15-S16). For postnatal SHS, adjustment for sustained maternal smoking in pregnancy, in general, attenuated the effect sizes to a maximum of around 20% change (Additional file 1: Table S17).

Third, we repeated the analysis restricting the sample to children of European ancestry (*N* = 1083) (Additional file 1: Table S18-S20). In general, *p* values were attenuated, likely due to a smaller samples size, but effect sizes remained of similar magnitude (mean absolute percentage change < 5%) (Additional file 3: Fig. S14, Additional file 1: Table S18). The other biomarkers showed more heterogeneous patterns when evaluated in children of European ancestry only, with some of them showing an increase of the effect (Additional file 1: Table S19-S20).

## Discussion

Despite the efforts of public health campaigns, maternal smoking in pregnancy and childhood SHS are still main adverse avoidable risk factors for child health. This study is the first to examine the association of exposure to tobacco smoking at different windows of exposure, in utero and in childhood, with multi-layer molecular phenotypes.

Exposure to maternal smoking in pregnancy was associated with DNA methylation of 41 CpG sites located in 18 different loci. All loci had previously been related to maternal smoking during pregnancy [14–16] or current smoking [21]. However, none of the previous studies had incorporated substantial transcriptomics data from the same subjects to interpret the functional consequences of these epigenetic changes. Furthermore, few studies investigated duration and intensity of maternal smoking in pregnancy, which might be relevant for public health advice [14, 16].

As in cord blood [14, 16], sustained maternal smoking in pregnancy produced larger effects on childhood blood DNA methylation than any maternal smoking, with the latter group including sustained smoker mothers as well as mothers that usually only smoked during the 1st trimester. Moreover, when considering duration and intensity of maternal smoking in pregnancy, some CpGs showed a dose-response trend, whereas others got saturated with any maternal smoking in pregnancy or did not have any meaningful response. These heterogeneous patterns, even in the same locus, can be explained by CpG-specific responses, but also by less accuracy in the measurement of some CpGs (low biological response to high technical noise) [66]. In any case, the persistence and the linear trend response of CpGs in *MYO1G*, *GNG12*, *AHRR*, *FRMD4A*, *RADIL*, and 7q11.22 make them interesting candidates for the development of an epigenetic biomarker for in utero exposure to smoking [10]. In general, maternal SHS during pregnancy had mostly negligible effects on offspring blood DNA methylation at the 41 significant CpGs, or at least their effects were diluted over time and not detected in childhood with the actual sample size.

We also observed that DNA methylation in 5 of these 18 loci was related to gene expression of nearby genes, but usually not of the closest annotated gene. However, these effects were weak as we did not detect significant associations between maternal smoking in pregnancy and expression of these genes. In other words, DNA methylation response to maternal smoking in pregnancy was not mirrored at the transcriptional level of nearby genes. Similarly, previous studies in former smokers have shown that smoking has a longer-lasting influence on the methylome compared to the transcriptome [67]. The reversal rate of gene expression at 1 year after smoking cessation has been calculated in > 50% and reaches > 85% after 10 years, whereas for methylation, it ranges from 17 to 33% with some effects still visible 40 years after smoking cessation. The different reversal rates between methylation and transcription could be explained by the complex transcriptional regulation that involves mechanisms other than DNA methylation. Whether persistent epigenetic marks act as a memory of the cell to previous exposures, to trigger rapid or amplified transcriptional activation in certain contexts (i.e., after a second exposure event), is unknown. Also, the weak association between exposure to tobacco smoke and transcription, in comparison to methylation, might be explained by the highest instability of the RNA compared to DNA, which might have introduced noise into the transcriptional data.

*AHRR* (*Aryl-Hydrocarbon Receptor Repressor*), which is involved in xenobiotic detoxification, cell growth, and differentiation, has widely been reported in relation to smoking. In particular, cg05575921 has been found to be hypo-methylated in cord blood [15], adult blood [21], placenta [13], and adipose tissue [67]. *AHRR* is an interesting example to discuss the complexity of epigenetic regulation. First, in our study, *AHRR* exhibited both hyper-methylation (intron 1) and hypo-methylation (other introns) in response to smoking. Through the eQTM analyses, we found that both hyper- and hypo-methylation were related to increased expression of the gene in blood. This finding evidences that epigenetic regulation of transcription is gene context-specific (i.e., intron 1 behaves different from other introns) and that methylation-expression correlations are fundamental to understand final transcriptional consequences. Second, besides *AHRR* gene, methylation at CpGs of this locus was also associated with the expression of two other nearby genes: *PDCD6* and *EXOC3*. *PDCD6* (Programmed Cell Death 6) is a calcium sensor involved in endoplasmic reticulum (ER)-Golgi vesicular transport, endosomal biogenesis, or membrane repair, and *EXOC3* (Exocyst Complex Component 3) is a component of the exocyst complex involved in the docking of exocytic vesicles with fusion sites on the plasma membrane. Further research might clarify the potential role of these genes, if any, in relation to tobacco smoking.

Two metabolites (lactate and alanine), known to be increased with glycemic dysregulation [68], were found at higher levels in urine of children born from sustained smoker mothers compared with non-smokers. Although there are some studies reporting an association between maternal smoking and type 2 diabetes and metabolic syndrome, the evidences are still inconclusive according to a recent meta-analysis [69].

In contrast to in utero exposure, exposure to childhood SHS, assessed through either questionnaire or cotinine, was associated with child serum metabolites and plasma proteins. In particular, we found that SHS, defined as urinary cotinine above the LOD, increased plasma PAI1 (plasminogen activator inhibitor-1 *SERPINE1* gene) protein levels. PAI1 is the principal inhibitor of tissue plasminogen activator (tPA) and urokinase (uPA), enzymes that convert plasminogen into plasmin (fibrinolysis) (Additional file 3: Fig. S15). Therefore, higher levels of PAI1 are indicative of a thrombotic state, and they have been found in active smokers [70, 71]. Although the increase of plasma PAI1 levels in SHS-exposed children was substantially smaller than the increase detected in active smokers [70], our findings evidence that SHS was sufficient to produce a pro-thrombotic state in children. The long-term consequences of this pro-thrombotic state in children, if prolonged over time, are unknown, but in adults, it is linked to age-related subclinical (i.e., inflammation or insulin resistance) or clinical (i.e., myocardial infarction, obesity) conditions [72].

We also found several serum metabolites altered in SHS-exposed children. Reduced levels of diacyl (aa)- and acyl-alkyl (ae)-phosphatidylcholines and of sphingomyelin (OH) C22:2 were in agreement with findings in active adult smokers [19]. Is it worth noting that these diacyl (aa)- and acyl-alkyl (ae)-phosphatidylcholines overlap with those positively associated with adherence to Mediterranean diet and with protective risk factors for cardiovascular disease [73]. We considered the reported location of childhood SHS exposure (inside home, outside, and in both places) as a surrogate of intensity of exposure to smoking. While PAI1 plasma protein levels and carnitine C9 were higher in children exposed in both locations, no clear dose-response patterns were observed for other metabolites.

Given that SHS effects might be subtler than those of active smoking, we also examined whether molecular features (CpG methylation or gene/miRNA transcription) described in current smokers overlapped with our findings of postnatal SHS with a less stringent *p* value cutoff. We did not detect any enrichment, suggesting that if postnatal SHS has an effect on these molecular layers, our sample size is too limited to detect it. Conversely, as an indication of the long-term and strong effects of active smoking, we did find enrichment for child CpGs associated with maternal smoking in pregnancy among CpGs described for current smoking in other studies. Indeed, it has been described a remarkable overlap between the blood methylation signatures detected in adult smokers and in newborns of smoker mothers [74].

Globally, our findings together with previous literature suggest that offspring blood DNA methylation captures strong and permanent effects associated with active maternal smoking during pregnancy. In our study, the associations between maternal smoking during pregnancy and DNA methylation were not attenuated after adjustment for childhood SHS, likely due the weaker effects of passive compared to active smoking. In contrast, the potential biological effects of SHS were best captured by dynamic molecules with fast responses, such as metabolites and proteins. Time-course studies will be needed to dissect this acute response in more detail. Adjustment for maternal smoking during pregnancy attenuated the effects of childhood SHS on these markers, highlighting the importance of mutually adjusted models in order to identify period-specific effects.

Findings should be considered within the context of the study’s limitations. First, exposure assessment to tobacco smoking, through either questionnaire or cotinine, has some intrinsic limitations. Maternal smoking in pregnancy and child exposure to SHS were self- or parental-reported, and they can be subject to misreporting [75]. Urinary cotinine, although more objective, only provides information about the most recent exposure (half-life in urine ~ 20 h) [8]. In our study, cotinine detection correlated strongly with the childhood SHS classification though, giving us reasonable confidence in the questionnaire reports. Second, we aimed to dissect pregnancy from childhood exposure associations using mutually adjusted models. However, misclassification and weaker effects of SHS compared to effects of maternal smoking in pregnancy (i.e., PAI1 or *AHRR* [23]), as well as the high level of overlap between maternal and childhood smoking exposure, might have limited our ability to distinguish between these two time periods. Larger samples of children exposed to SHS, without in utero exposure, might be needed to investigate SHS, especially for DNA methylation. Third, although the statistical models were adjusted for an exhaustive list of confounders, including child zBMI, we cannot completely rule out residual confounding. For example, plasma PAI1, which can be released by fat cells [76], is related to BMI and percent body fat [77]. Fourth, although the study has been designed as a comprehensive screening using high-throughput omics platforms, these platforms do not have complete coverage of the molecular layers, and consequently, we might have missed some biological signals. Also, cell type-specific responses might have been diluted within the context of whole blood analyses. Fifth, some of the smoking sensitive CpGs detected in our study are known to be regulated by mQTLs. The role of genetic variation in modifying the effects of the exposure to smoking deserves future research. Finally, our study predominantly consisted of European ancestry children, and thus, additional studies involving diverse ethnic backgrounds are needed in order to improve the generalizability of the findings. Potential confounding by ancestry was controlled by adjusting the models for self-reported ethnic origin. For the top signals, we also performed a sensitivity analysis restricted to European ancestry children and results did not change substantially.

## Conclusions

Our study investigated the in utero and postnatal effects of exposure to tobacco smoke on 4 molecular layers, assessed through a harmonized protocol, in 1203 children across Europe. Our results confirmed previous findings of persistent associations between maternal smoking in pregnancy and childhood blood DNA methylation and showed dose-response trends at some CpG sites. These might be informative for the development of an epigenetic risk score of in utero exposure to tobacco smoke. The persistent methylation signature related to in utero exposure to smoking was not mirrored at the transcriptional level. The meaning of the gap between methylation and transcriptional signals requires further investigation, but might mean a methylation-based memory of the cell. Childhood SHS was not related to blood methylation, indicating much weaker effects of recent SHS with respect to active maternal smoking in pregnancy. In contrast, childhood SHS was related to higher plasma levels of PAI1, a protein that inhibits fibrinolysis, and to certain metabolites. The final clinical impact of sustained increased levels of PAI1 in children is unknown, but this finding highlights the importance of the analysis of molecular traits to capture subtler effects at earlier timepoints.

## Supplementary information

**Additional file 1: Table S1.** Years of enrollment in the cohort, years of HELIX vist and years when smoking was banned in each cohort (country)**. Table S2.** Proteins targeted in each of the three Magnetic Human Luminex kits from Life Technologies**. Table S3.** Association of maternal smoking in pregnancy and child blood DNA methylation levels adjusted for global-SHS, ordered by chromosome position**. Table S4.** eQTM analyses: Association between child blood DNA methylation levels at 41 CpG sites vs. child blood expression levels of genes within ±500 kb, ordered by *p*-value**. Table S5.** Association between maternal smoking in pregnancy and child blood expression levels of top genes, ordered by p-value of sustained maternal smoking in pregnancy**. Table S6.** Association between maternal smoking in pregnancy and child blood expression levels of genes within ±500 kb, ordered by *p*-value of sustained- maternal smoking in pregnancy**. Table S7.** Association of maternal smoking in pregnancy and child molecular phenotypes adjusted for global-SHS**. Table S8.** Association of childhood SHS and child molecular phenotpyes adjusted for sustained maternal smoking in pregnancy. **Table S9.** Comparison of the association of own smoking in adults and SHS in children with serum metabolites, ordered by metabolite name**. Table S10.** Comparison of the association of own smoking in adults and SHS in children with blood miRNA expression, ordered by miRNA name**. Table S11.** Comparison of the association of own smoking in adults and SHS in children with blood gene expression, ordered by p-value in children (sustained maternal smoking in pregnancy)**. Table S12.** Comparison of the association of own smoking in adults and SHS in children with blood DNA methylation, ordered by p-value in children (sustained maternal smoking in pregnancy)**. Table S13.** Association of maternal smoking in pregnancy and child DNA methylation levels adjusted for home-SHS**. Table S14.** Association of maternal smoking in pregnancy and child molecular phenotypes adjusted for home-SHS**. Table S15.** Association of maternal smoking in pregnancy and child DNA methylation levels unadjusted for global-SHS**. Table S16.** Association of maternal smoking in pregnancy and child molecular phenotypes unadjusted for global-SHS**. Table S17.** Association of childhood SHS and child molecular phenotypes unadjusted for sustained maternal smoking in pregnancy**. Table S18.** Association of maternal smoking in pregnancy and child DNA methylation levels in European ancestry children adjusted for global-SHS**. Table S19.** Association of maternal smoking in pregnancy and child molecular phenotypes in European ancestry children adjusted for global-SHS**. Table S20.** Association of childhood SHS and child molecular phenotypes in European ancestry children adjusted for sustained maternal smoking in pregnancy

**Additional file 2 Additional Methods**.

**Additional file 3: Additional Fig. S1-S5 and Fig. S7-S15. Fig. S1.** Directed acyclic graph (DAG) with causal assumptions from a priori knowledge between maternal smoking *during* pregnancy *(MSDP)* and child molecular features. In blue, the causal path assessed in the model. **Fig. S2.** Directed acyclic graph (DAG) with causal assumptions from a priori knowledge between child*hood* SHS and child molecular features. **Fig. S3.** Percentage of children exposed to tobacco smoking in the study population and by cohort: any (A) and sustained (B) maternal smoking during pregnancy (MSDP), childhood global-SHS (C), and child urinary cotinine measurements (D). **Fig. S4.** QQ-plot and Volcano-plot of the associations between child DNA methylation and any (A and B) and sustained (C and D) maternal smoking during pregnancy (MSDP), adjusted for global-SHS. **Fig. S5.** Comparison of effects on child blood DNA methylation between any and sustained maternal smoking during pregnancy (MSDP), adjusted for global-SHS. **Fig. S7**. Plot showing significance of methylation to expression relationships (−log10(p-value)) in relation to the distance between TC-TSS and CpG. **Fig. S8.** QQ-plot of the associations between child blood gene expression and global-SHS (A) and urinary cotinine (B), adjusted for sustained maternal smoking during pregnancy (MSDP), among 1270 genes identified in current smokers at 10% FDR (Huan et al. 2016). **Fig. S9.** QQ-plot of the associations between child blood DNA methylation and global-SHS (A) and urinary cotinine (B), adjusted for sustained maternal smoking during pregnancy (MSDP), among 18,763 CpGs identified in current smoking at 5% FDR (Joehanes et al. 2016). **Fig. S10.** QQ-plot of the associations between child blood DNA methylation and any (A) and sustained (B) maternal smoking during pregnancy (MSDP), adjusted for global-SHS, among 18,763 CpGs identified in current smoking at 5% FDR (Joehanes et al. 2016). **Fig. S11**. Interaction between any maternal smoking during pregnancy (MSDP) and global-SHS. The y-axis shows predicted methylation levels at cg01664727 (at *RUNX1* locus). **Fig. S12.** Comparison of effects of maternal smoking during pregnancy (MSDP) on child blood DNA methylation between models adjusted for global-SHS and for home-SHS. **Fig. S13.** Comparison of effects of maternal smoking during pregnancy (MSDP) on child blood DNA methylation between models adjusted for global-SHS and unadjusted model. **Fig. S14.** Comparison of effects of maternal smoking during pregnancy (MSDP) on child blood DNA methylation between datasets including all children and including only European ancestry children. **Fig. S15**. Schematic representation of PAI1 cascade. In red inhibition steps and in green activation steps.

**Additional file 4: Fig. S6. Fig. S6.** Box plots showing the change of child blood DNA methylation compared to unexposed mothers at 41 CpGs (y-axis) by categories of dose and/or duration of exposure to tobacco smoking in pregnancy (x-axis), adjusted for global-SHS. Horizontal line in the middle of the boxes shows the mean difference in DNA methylation with respect to the reference category of unexposed mothers. Boxes represents the DNA methylation change ± standard error (SE), and vertical lines indicate extreme changes defined as ±3xSE. Legend: Mat-SHS (mothers exposed to SHS), Non-sust (non-sustained smoker mothers), Sust (= < 9) (Sustained smoker mothers at low dose – less than or equal to 9 cigarettes per day), Sust (> 9) (Sustained smoker mothers at high dose – more than 9 cigarettes per day). Other categories are self-explanatory.

## Data Availability

Summarized results of each model (exposure, marker, effect, SE, *p* value) will be uploaded on the HELIX webpage (https://helixomics.isglobal.org/). Raw data can be obtained under request, after signature of a Data Transfer Agreement (DTA).

## Abbreviations

BMI: Body mass index
CV: Coefficient of variation
DAG: Directed acyclic graph
eQTM: Expression quantitative trait methylation
FC: Fold change
LOD: Limit of detection
LOQ: Limit of quantification
miRNA: MicroRNA
mQTL: Methylation quantitative trait locus
MSDP: Maternal smoking during pregnancy
QC: Quality control
SE: Standard error
SHS: Secondhand smoke
SVA: Surrogate variable analysis
TC: Transcript cluster
TSS: Transcription start site
