# Supplementary material for: In utero and childhood exposure to tobacco smoke and multi-layer molecular signatures in children
Source: BMC Med. 2020 Aug 19;18:243. doi: 10.1186/s12916-020-01686-8 (PMC7437049; doi:10.1186/s12916-020-01686-8)
Supplement: Supplementary file 2 — Additional file 2 Additional Methods. [file 12916_2020_1686_MOESM2_ESM.docx]

***In utero* and childhood exposure to tobacco smoke and multi-layer molecular signatures in children**

**Additional Methods**

***Blood DNA methylation***

DNA was extracted from children’s buffy coat using the Chemagen kit (Perkin Elmer, USA). Samples were not randomized during DNA extraction, but they were extracted by order of arrival (first one cohort, then another, etc.). DNA concentration was determined in a Nanodrop 1000 UV-Vis Spectrophotometer (Thermo Fisher Scientific, USA) and with the Quant-iT^TM^ PicoGreen^®^ dsDNA Assay Kit (Life Technologies, USA).

DNA methylation was assessed with the Infinium HumanMethylatio450 beadchip (Illumina, USA) at the University of Santiago de Compostela – Spanish National Genotyping Center (CeGen-USC, Spain). 700 ng of DNA were bisulfite-converted using the EZ 96-DNA kit (Zymo Research, USA). All samples of the study were randomized and balanced by sex and cohort within each batch. In addition, each plate contained a HapMap control sample and a total of 24 inter-plate duplicates were included.

Probes with a call rate <95% based on a detection p-value of 1E-16 (Lehne et al. 2015), and samples with a call rate <98% were removed. Then, samples with discordant sex (derived from epigenetic data vs. recorded), duplicates with discordant genotypes, and samples with discordant genotypes respect to existing genome-wide genotyping array data, were eliminated. Methylation data was normalized using the functional normalization method with prior background correction with Noob (Fortin et al. 2014). Some additional probes were filtered out: control probes, probes to detect single nucleotide polymorphisms (SNPs), probes to detect methylation in non-CpG sites, probes located in sexual chromosomes, cross hybridizing probes (Y. Chen et al. 2013), probes containing a SNP at any position of the sequence with a minor allele frequency (MAF) >5% and probes with a SNP at the CpG site or at the single base extension (SBE) at any MAF in the combined population from 1000 Genomes Project. CpGs were annotated with the IlluminaHumanMethylation450kanno.ilmn12.hg19 R package (Hansen. K. D. 2012). Finally, slide batch effect was controlled with the ComBat R package (Johnson, Li, and Rabinovic 2007).

***Blood gene expression***

RNA was extracted from whole blood collected in Tempus tubes using the MagMAX for Stabilized Blood Tubes RNA Isolation kit (Thermo Fisher Scientific, USA). Samples were not randomized during RNA extraction, but they were extracted by order of arrival (first one cohort, then another, etc.). The quality of RNA was evaluated with a 2100 Bioanalyzer (Agilent Technologies, USA) and the concentration with a NanoDrop 1000 UV-Vis Spectrophotometer. Samples classified as good RNA quality, which were subsequently analyzed, had a RNA Integrity Number (RIN) >5, a similar RNA pattern at visual inspection and a concentration >10 ng/ul. Mean values for the RIN, concentration (ng/ul), Nanodrop 260/280 and 260/230 ratios were: 6.99, 107.36, 2.15 and 0.61.

Gene expression was assessed using the GeneChip^®^ Human Transcriptome Array 2.0 (HTA 2.0) (Affymetrix, USA) at the University of Santiago de Compostela (USC, Spain), following Affymetrix recommendations, and starting from 200 ng of total RNA. All samples of the study were randomized and balanced by sex and cohort within each batch. Two different types of control RNA samples (HeLa and FirstChoice^®^ Human Brain Reference RNA (Thermo Fisher Scientific, USA)) were included in each batch, but they were hybridized only in the first batches.

Raw data were extracted with the Affymetrix AGCC software and normalized at the gene level with the GCCN (SST-RMA) algorithm. Gene expression values were log2 transformed. Annotation of transcript clusters (TCs - a group of one or more probes covering a region of the genome) to genes was done with the Affymetrix ExpressionConsole software using the HTA-2_0 Transcript Cluster Annotations Release na36 (hg19). Four samples with discordant sex (derived from transcriptomic data vs. recorded) were detected with the MassiR R package (Buckberry et al. 2014) and excluded. Control probes, and TCs in sexual chromosomes and without chromosome information were filtered out. In order to determine TC call rate, 10 constitutive or best probes based on probe scoring and cross-hybridation potential were selected per TC. Probe Detection Above Background (DABG) p-values were computed based on the rank order against the background probe set intensities. Probe level p-values were combined into a TC level p-value using the Fisher equation. TCs with a DABG p-value <0.05 were defined as detected. TCs with a Probe Detection Above Background (DABG) p-value <0.05 were defined as detected. Two samples with low call rate (<40%) as well as TCs with a call rate <1% were excluded from the dataset. Additional control of batch effects and blood cell type composition was done with two surrogate variable analyses (SVA) methods, isva (Teschendorff, Zhuang, and Widschwendter 2011) and SmartSVA (J. Chen et al. 2017) , implemented in omicRexposome R package (Hernandez-Ferrer et al. 2019).

***Blood miRNA expression***

miRNA expression was quantified using the SurePrint Human miRNA Microarray rel.21 (Agilent Technologies, USA) (Mestdagh et al. 2014), at the Genomics Core facility at the Centre for Genomic Regulation (CRG, Spain).

After sample randomization by sex and cohort, 24 samples were processed per batch and hybridized in 3 different slides, starting from 100 ng of total RNA. A commercial control sample, a total RNA mixture from 9 human tissues or cell lines (Agilent Technologies, USA), was included in 2/3 of the batches. Raw data was extracted with the Agilent Feature extraction software. Six samples were removed due to overall low quality.

miRNA expression levels were normalized with the least variant set (LVS) method (Suo et al. 2010) with background correction with the Normexp method implemented in the limma package (Ritchie et al. 2015). LVS normalization method builds upon the fact that the data-driven housekeeping miRNAs, the least variant across samples, might be a good reference set for normalization. For the identification of housekeeping miRNAs, a random set of 50 HELIX samples was used. For the identification of housekeeping miRNAs, a random set of 50 HELIX samples was used. Normalization using the selected miRNA reference set was done by applying the variance stabilization and calibration for microarray data (vsn) method. Normalized miRNA levels were log2 transformed and annotated using a combination of information from Agilent annotation (“Annotation_7056”) and miRbase v21 (GRCh38 and mapped back to hg19) released in January 2017. Then, control probes, miRNAs in sexual chromosomes, unannotated miRNA, and miRNAs with a call rate <1% were filtered out. A miRNA was considered as detected if its expression was different from the background or the standard error of its different probes was smaller than 3 times the expression signal. Additional control of batch effect and blood cell composition during the differential expression analyses was done with the SVA standard method (Leek et al. 2012), implemented in omicRexposome R package (Hernandez-Ferrer et al. 2019).

***Plasma proteins***

Plasma protein levels were assessed using the antibody-based multiplexed platform from Luminex at the Proteomics Unit (Centre for Genomic Regulation (CRG) / University Pompeu Fabra (UPF), Spain). Three kits targeting 43 unique candidate proteins were selected (Thermo Fisher Scientifics, USA): Cytokines 30-plex (Catalog Number (CN): LHC6003M), Apoliprotein 5-plex (CN: LHP0001M) and Adipokine 15-plex (CN: LHC0017M).

All samples were randomized and blocked by cohort. For quantification, an 8-point calibration curve per plate was performed with protein standards provided in the Luminex kit and following procedures described by the vendor. Commercial heat inactivated, sterile-filtered plasma from human male AB plasma (Sigma-Aldric, USA) was used as constant controls to control for intra- and inter-plate variability. Four control samples were added per plate. All samples, including controls, were diluted ½ for the 30-plex kit, ¼ for the 15-plex kit and 1/2500 for the 5-plex kit.

Raw intensities obtained with the xMAP and Luminex system for each plasma sample were converted to ng/ml (5-plex kit) and to pg/ml (15 and 30-plex kits) using the calculated standard curves of each plate and accounting for the dilutions made prior measurement. The percentages of coefficients of variation (CV%) for each protein by plate ranged from 3% to 36%. The limit of detection (LOD) and the lower and upper limit of quantification (LOQ1 and LOQ2, respectively) were estimated by plate, and then averaged. Only proteins with >30% of measurements in the linear range of quantification were kept in the database and the others were removed. Seven proteins were measured twice (in two different multiplex kits). We kept the measure with higher quality. The 36 proteins that passed the quality control criteria mentioned above were log2 transformed. Then, the plate batch effect was corrected by subtracting the plate specific average for each protein minus the overall average of all plates for that protein. After that, values below the lower limit of quantification (LOQ1) and above the upper limit of quantification (LOQ2) were imputed using a truncated normal distribution implemented in the truncdist R package (Nadarajah and Kotz 2006). Twenty samples were excluded due to having ten or more proteins out of the linear range of quantification.

***Serum metabolites***

Serum metabolites were quantified using the targeted metabolomics Absolute-*IDQ*^TM^ p180 Kit (Biocrates Life Sciences AG, USA), which allows the targeted analysis of 188 metabolites in the classes of amino acids, biogenic amines, acylcarnitines, glycerophospholipids, sphingolipids and sum of hexoses, covering a wide range of analytes and metabolic pathways in one targeted assay. The kit consists of a single sample processing procedure, although two separate mass spectrometry (MS) analytical runs are applied. Of the total 188 metabolites, 42 were analyzed quantitatively by liquid chromatography (LC)-Electrospray Ionization (ESI)-MS/MS with the use of external calibration standards at seven different concentrations and isotope labelled internal standards for most analytes. The other 146 metabolites were analyzed by flow-injection analysis (FIA)-ESI-MS/MS using a one-point internal standard calibration with representative internal standards.

Serum metabolic profiles were acquired on a Sciex QTrap 6500 equipped with an Agilent 1100 series HPLC (Agilent Technologies, USA), at Imperial College of London (ICL, UK), according to the manufacturer protocol (Lau et al. 2018). The metabolomics data of serum samples were acquired in 18 batches in 96-well plate format and samples were fully randomized. Every plate included 3 phosphate-buffered saline (PBS) blanks, 7 calibration standards and three sets of quality control samples (the human plasma-based quality controls provided by the manufacturer in three different concentrations, the NIST SRM 1950 refernce material (Sigma-Aldrich, USA) and a commercially available serum QC material (CQC, SeraLabs, S-123-M-2748)).

Metabolites were quantified (mM) following the manufacturer’s protocol using MetIDQTM Boron software (Biocrates Life Scineces AG, USA), and then log2 transformed. The median value of the PBS samples on a plate was calculated as approximation of the background noise per metabolite, and LOD was defined as 3 times this value (per metabolite and per batch). CV% was calculated from the 4 replicates of the NIST SRM 1950 control sample.

Metabolite exclusion was based on a metabolite variable meeting two conditions: (1) CV of over 30% and (2) over 30% of the data are below LOD. Eleven out of the 188 serum metabolites detected were excluded as a result, leaving 177 serum metabolites to be used for further statistical analysis. The mean coefficient of variation across the 177 LC-MS/MS detected serum metabolites was 16%. Analytical performance was in line with expectations from and inter-laboratory ring trial of this platform (Siskos et al. 2017).

***Urinary metabolites***

Urinary metabolic profiles were analyzed on a 14.1 Tesla (600 MHz ^1^H) NMR spectrometer (Bruker, USA) at ICL (UK) (Lau et al. 2018). Urine samples (pool, morning or bedtime) were fully randomized to prevent potential bias. In addition, 60 identical study quality control urine samples were included at regular intervals during the runs. Whilst data acquisitions were untargeted, data processing workflow followed a targeted strategy to identify and quantify the 44 most abundant metabolites in urine.

1D presat-NOESY spectra were imported into MATLAB (MathWorks, USA) and aligned to the pooled quality control sample using recursive segment-wise peak alignment (Lau et al. 2018)(Veselkov et al. 2009). Poor peak alignment may result from multiple peak features occupying the same spectral region thus causing a lost in feature selectivity. Thus, it was also assessed the peak alignment for individual sample metabolite signals and computed Pearson’s correlation coefficients (R2) between the aligned peak segment and the reference peak segment. Finally, metabolite peak signals were estimated using trapezoidal numerical integration.

CVs were assessed based on repeated analysis of the 60 identical quality control urine samples. The vast majority of metabolites achieved CV <10%.

Sample concentration of a given metabolite was estimated from the signal of the internal standard trimethylsilylpropanoic (TSP). Data was normalized using the median fold change normalization method which takes into account the distribution of relative levels from all 44 metabolites compared to the reference sample in determining the most probable dilution factor. Twenty-six metabolites were absolutely quantified, and 18 semi-quantified. Concentration levels were expressed as log2, and before that we used an off-set of ½ the minimal value for each metabolite.
