# Supplementary material for: In utero and childhood exposure to tobacco smoke and multi-layer molecular signatures in children
Source: BMC Med. 2020 Aug 19;18:243. doi: 10.1186/s12916-020-01686-8 (PMC7437049; doi:10.1186/s12916-020-01686-8)
Supplement: Supplementary file 3 — Additional file 3: Additional Fig. S1-S5 and Fig. S7-S15. Fig. S1. Directed acyclic graph (DAG) with causal assumptions from a priori knowledge between maternal smoking during pregnancy (MSDP) and child molecular features. In blue, the causal path assessed in the model. Fig. S2. Directed acyclic graph (DAG) with causal assumptions from a priori knowledge between childhood SHS and child molecular features. Fig. S3. Percentage of children exposed to tobacco smoking in the study population and by cohort: any (A) and sustained (B) maternal smoking during pregnancy (MSDP), childhood global-SHS (C), and child urinary cotinine measurements (D). Fig. S4. QQ-plot and Volcano-plot of the associations between child DNA methylation and any (A and B) and sustained (C and D) maternal smoking during pregnancy (MSDP), adjusted for global-SHS. Fig. S5. Comparison of effects on child blood DNA methylation between any and sustained maternal smoking during pregnancy (MSDP), adjusted for global-SHS. Fig. S7. Plot showing significance of methylation to expression relationships (−log10(p-value)) in relation to the distance between TC-TSS and CpG. Fig. S8. QQ-plot of the associations between child blood gene expression and global-SHS (A) and urinary cotinine (B), adjusted for sustained maternal smoking during pregnancy (MSDP), among 1270 genes identified in current smokers at 10% FDR (Huan et al. 2016). Fig. S9. QQ-plot of the associations between child blood DNA methylation and global-SHS (A) and urinary cotinine (B), adjusted for sustained maternal smoking during pregnancy (MSDP), among 18,763 CpGs identified in current smoking at 5% FDR (Joehanes et al. 2016). Fig. S10. QQ-plot of the associations between child blood DNA methylation and any (A) and sustained (B) maternal smoking during pregnancy (MSDP), adjusted for global-SHS, among 18,763 CpGs identified in current smoking at 5% FDR (Joehanes et al. 2016). Fig. S11. Interaction between any maternal smoking during pregnancy (MSDP) and [file 12916_2020_1686_MOESM3_ESM.docx]

***In utero* and childhood exposure to tobacco smoke and multi-layer molecular signatures in children**

**Additional Figures**

**
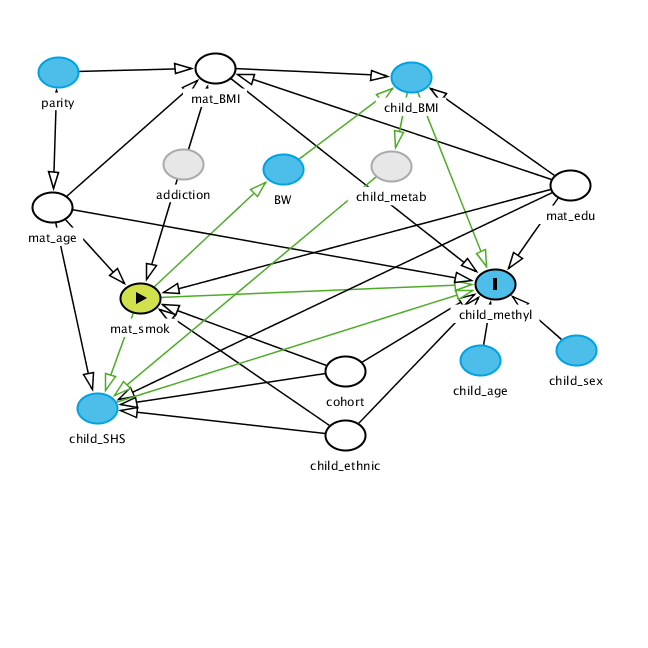
**

A


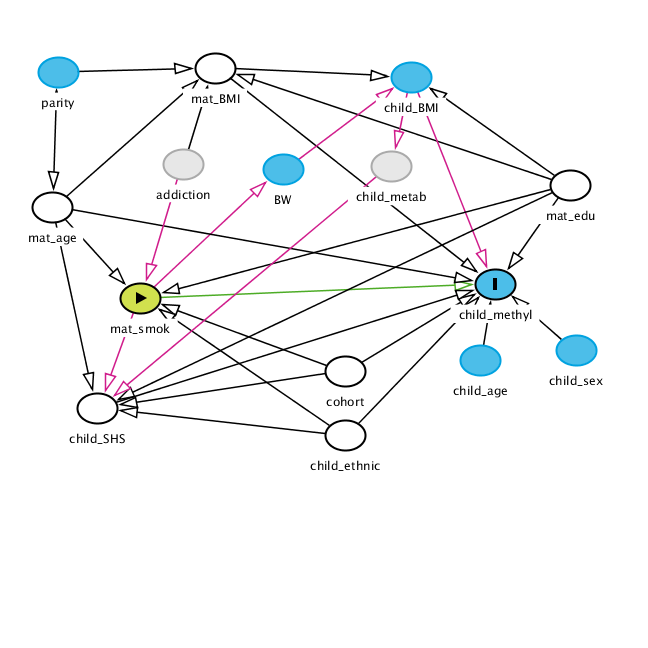


B

**Figure S1.** Directed acyclic graph (DAG) with causal assumptions from a priori knowledge between maternal smoking during pregnancy and child molecular features. A) Total effect of maternal smoking during pregnancy on child molecular features. In green, the causal paths assessed in the model. In white, covariates proposed as confounders by the DAGitty tool: cohort, child ethnic origin, maternal BMI, maternal age and maternal education. In blue, covariates not proposed as confounders by DAGitty, but included in the models. Sex and child age were selected as they are main predictors of the outcome that can increase precision in the association with the exposure. In grey, unmeasured covariates. B) Effect of maternal smoking during pregnancy on child molecular features, independent of childhood SHS. In addition to the variables above, we additionally adjusted the models for childhood SHS. Still some indirect paths from maternal smoking during pregnancy on child molecular features through child BMI are open (indicated in pink).


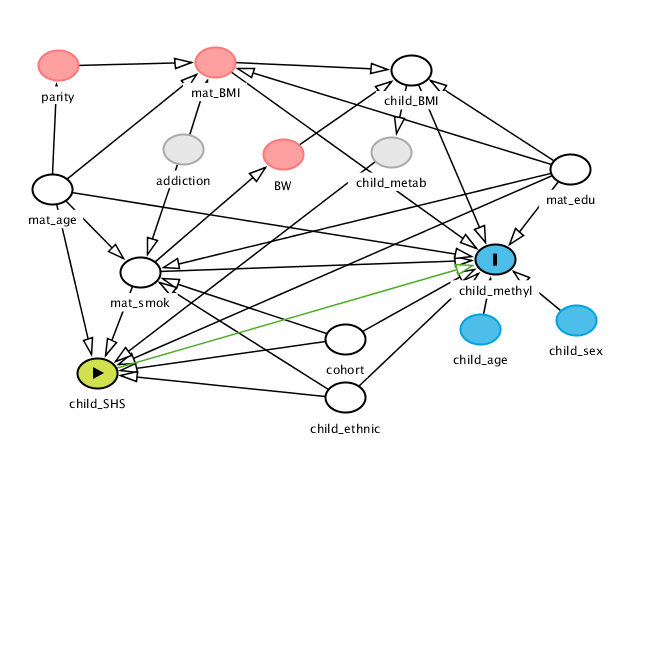


**Figure S2.** Directed acyclic graph (DAG) with causal assumptions from a priori knowledge between childhood SHS and child molecular features. In green, the causal path assessed in the model. In white, covariates proposed as confounders by the DAGitty tool: cohort, child ethnic origin, child BMI, maternal age, maternal education and maternal smoking during pregnancy. Maternal smoking in pregnancy was included in order to assess the total effects of childhood SHS on child methylation, independent of pregnancy effects. In blue, covariates not proposed by DAGitty but included in the models: sex and child age. Sex and child age were selected as they are main predictors of the outcome that can increase precision in the association with the exposure. In red, covariates not proposed by DAGitty tool. In grey, unmeasured covariates.


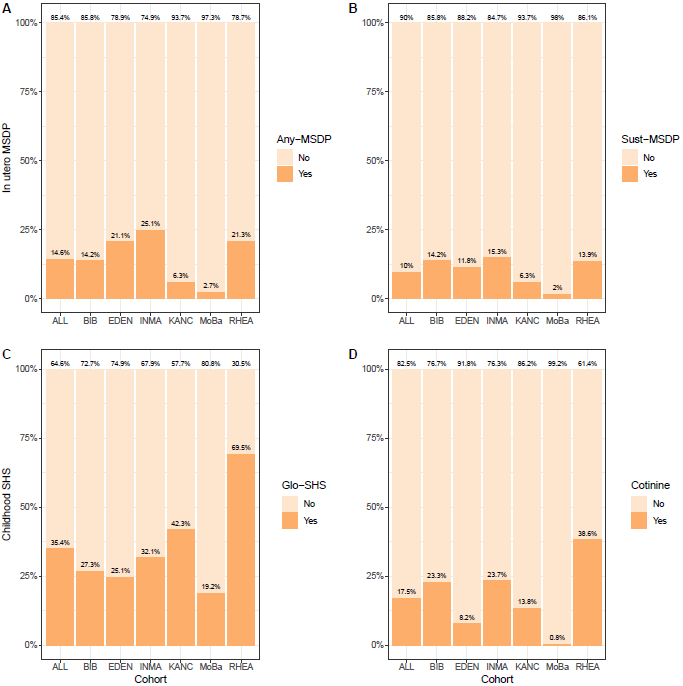


**Figure S3.** Percentage of children exposed to tobacco smoking in the study population and by cohort: any (A) and sustained (B) maternal smoking during pregnancy (MSDP), childhood global-SHS (C), and child urinary cotinine measurements (D).

**Figure S4.** QQ-plot and Volcano-plot of the associations between child DNA methylation and any (A and B) and sustained (C and D) maternal smoking during pregnancy (MSDP), adjusted for global-SHS. In the QQ-plot, the x-axis represents the expected –log10(p-value) and the y-axis the observed –log10(p-value). Lambda inflation factors were 1.003 (A) and 0.951 (C). In the volcano plots, the x-axis shows the change in DNA methylation (effect size) and the y-axis the statistical significance (-log10(p-value)). 5% FDR-significant CpGs are shown in red. Among them, 29.6 % (B) and 31.6 % (D) were hypo-methylated.

**Figure S5.** Comparison of effects on child blood DNA methylation between any and sustained maternal smoking during pregnancy (MSDP), adjusted for global-SHS. Dots represent effect sizes on child DNA methylation of any maternal smoking in pregnancy (x-axis) and sustained maternal smoking in pregnancy (y-axis). The mean absolute difference between any and sustained maternal smoking in pregnancy in the 41 FDR-significant CpGs was 32.8%. Effect sizes are expressed as the change in DNA methylation of exposed children versus unexposed children.

**Figure S6.** Box plots showing the change of child blood DNA methylation with respect to unexposed mothers at 41 CpGs (y-axis) by categories of dose and/or duration of exposure to tobacco smoking in pregnancy (x-axis), adjusted for global-SHS. Horizontal line in the middle of the boxes shows the mean difference in DNA methylation with respect to the reference category of unexposed mothers. Boxes represents the DNA methylation change ± standard error (SE), and vertical lines indicate extreme changes defined as ± 3xSE. Legend: Mat-SHS (mothers exposed to SHS), Non-sust (non-sustained smoker mothers), Sust (=<9) (Sustained smoker mothers at low dose – less than or equal to 9 cigarettes per day), Sust (>9) (Sustained smoker mothers at high dose – more than 9 cigarettes per day). Other categories are self-explanatory.

**FOLDER: HELIX_smk_omics_SupFig6_draft3_20191212**


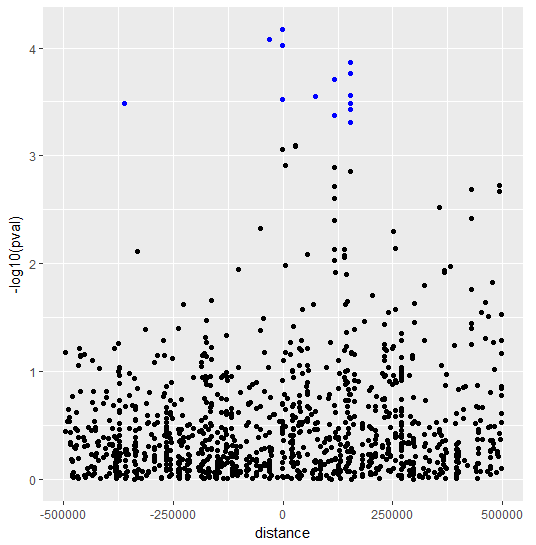


**Figure S7**. Plot showing significance of methylation to expression relationships (-log10(p-value)) in relation to the distance between TC-TSS and CpG. In blue, associations at 5% FDR.

**Figure S8.** QQ-plot of the associations between child blood gene expression and any (A) and sustained (B) maternal smoking during pregnancy (MSDP), adjusted for global-SHS, among 1270 genes identified in current smokers at 10% FDR (Huan et al. 2016). The x-axis represents the expected –log10(p-value) and the y-axis the observed –log10(p-value). Lambda inflation factors were 0.91 (A) and 0.96 (B). Statistical significance for enrichment (one-sided Kolmogórov-Smirnov test) were 0.903 (A) and 0.842 (B). The number of significant CpGs at p-value <0.05 and the % of them showing a consistent direction of the effect were: 56 (53.6%) (A) and 61 (49.1%) (B).

**Figure S9.** QQ-plot of the associations between child blood gene expression and global-SHS (A) and urinary cotinine (B), adjusted for sustained maternal smoking during pregnancy (MSDP), among 1270 genes identified in current smokers at 10% FDR (Huan et al. 2016). The x-axis represents the expected –log10(p-value) and the y-axis the observed –log10(p-value). Lambda inflation factors were 0.92 (A) and 0.99 (B). Statistical significance for enrichment (one-sided Kolmogórov-Smirnov test) were 0.579 (A) and 0.746 (B). The number of significant CpGs at p-value <0.05 and the % of them showing a consistent direction of the effect were: 60 (53.3%) (A) and 62 (51.2%) (B).

**Figure S10.** QQ-plot of the associations between child blood DNA methylation and global-SHS (A) and urinary cotinine (B), adjusted for sustained maternal smoking during pregnancy (MSDP), among 18763 CpGs identified in current smoking at 5% FDR (Joehanes et al. 2016). The x-axis represents the expected –log10(p-value) and the y-axis the observed –log10(p-value). Lambda inflation factors were 1.01 (A) and 0.89 (B). Statistical significance for enrichment (one-sided Kolmogórov-Smirnov test) were 0.034 (A) and 0.998 (B). The number of significant CpGs at p-value <0.05 and the % of them showing a consistent direction of the effect were: 755 (60.8%) (A) and 563 (41.0%) (B).

**Figure S11.** QQ-plot of the associations between child blood DNA methylation and any (A) and sustained (B) maternal smoking during pregnancy (MSDP), adjusted for global-SHS, among 18763 CpGs identified in current smoking at 5% FDR (Joehanes et al. 2016). The x-axis represents the expected –log10(p-value) and the y-axis the observed –log10(p-value). Lambda inflation factors were 1.08 (A) and 1.16 (B). Statistical significance for enrichment (one-sided Kolmogórov-Smirnov test) were 2.834E-07 (A) and <2.2E-16 (B). The number of significant CpGs at p-value <0.05 and the % of them showing a consistent direction of the effect were: 979 (71.2%) (A) and 1279 (73.3%) (B). The number of significant CpGs at 5% FDR and the % of them showing a consistent direction of the effect were: 21 (66.7%) (A) and 24 (66.7%) (B).

**Figure S12.** Comparison of effects of maternal smoking during pregnancy (MSDP) on child blood DNA methylation between models adjusted for global-SHS and for home-SHS. Dots represent effect sizes on child DNA methylation of maternal smoking in pregnancy adjusted for global-SHS (x-axis) and adjusted for home-SHS (y-axis), for any (A) and sustained (B) maternal smoking in pregnancy. Effect sizes are expressed as the change in DNA methylation of exposed children versus unexposed children.

**Figure S13.** Comparison of effects of maternal smoking during pregnancy (MSDP) on child blood DNA methylation between model adjusted for global-SHS and model unadjusted for postnatal SHS. Dots represent effect sizes on child DNA methylation of maternal smoking in pregnancy adjusted for global-SHS (x-axis) and unadjusted for postnatal SHS (y-axis), for any (A) and sustained (B) maternal smoking in pregnancy. Effect sizes are expressed as the change in DNA methylation of exposed children versus unexposed children.

**Figure S14.** Comparison of effects of maternal smoking during pregnancy (MSDP) on child blood DNA methylation between datasets including all children and including only European ancestry children. Dots represent effect sizes on child DNA methylation of maternal smoking in pregnancy adjusted for global-SHS in all children (x-axis) and in European ancestry children (y-axis), for any (A) and sustained (B) maternal smoking in pregnancy. Effect sizes are expressed as the change in DNA methylation of exposed children versus unexposed children.


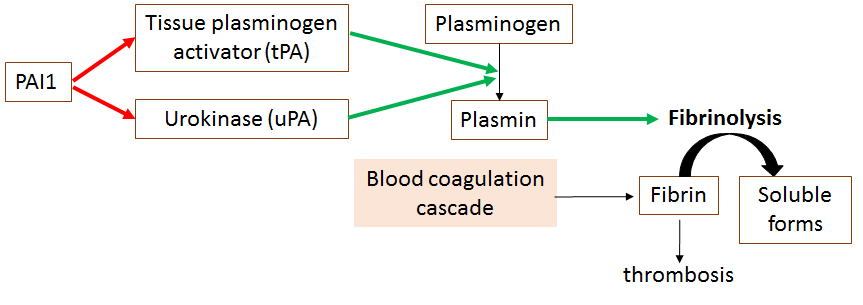


**Figure S15**. Schematic representation of PAI1 cascade. In red inhibition steps and in green activation steps.
