# Supplementary material for: In utero and childhood exposure to tobacco smoke and multi-layer molecular signatures in children
Source: BMC Med. 2020 Aug 19;18:243. doi: 10.1186/s12916-020-01686-8 (PMC7437049; doi:10.1186/s12916-020-01686-8)
Supplement: Supplementary file 4 — Additional file 4: Fig. S6. Fig. S6. Box plots showing the change of child blood DNA methylation compared to unexposed mothers at 41 CpGs (y-axis) by categories of dose and/or duration of exposure to tobacco smoking in pregnancy (x-axis), adjusted for global-SHS. Horizontal line in the middle of the boxes shows the mean difference in DNA methylation with respect to the reference category of unexposed mothers. Boxes represents the DNA methylation change ± standard error (SE), and vertical lines indicate extreme changes defined as ±3xSE. Legend: Mat-SHS (mothers exposed to SHS), Non-sust (non-sustained smoker mothers), Sust (= < 9) (Sustained smoker mothers at low dose – less than or equal to 9 cigarettes per day), Sust (> 9) (Sustained smoker mothers at high dose – more than 9 cigarettes per day). Other categories are self-explanatory. [file 12916_2020_1686_MOESM4_ESM.pdf]

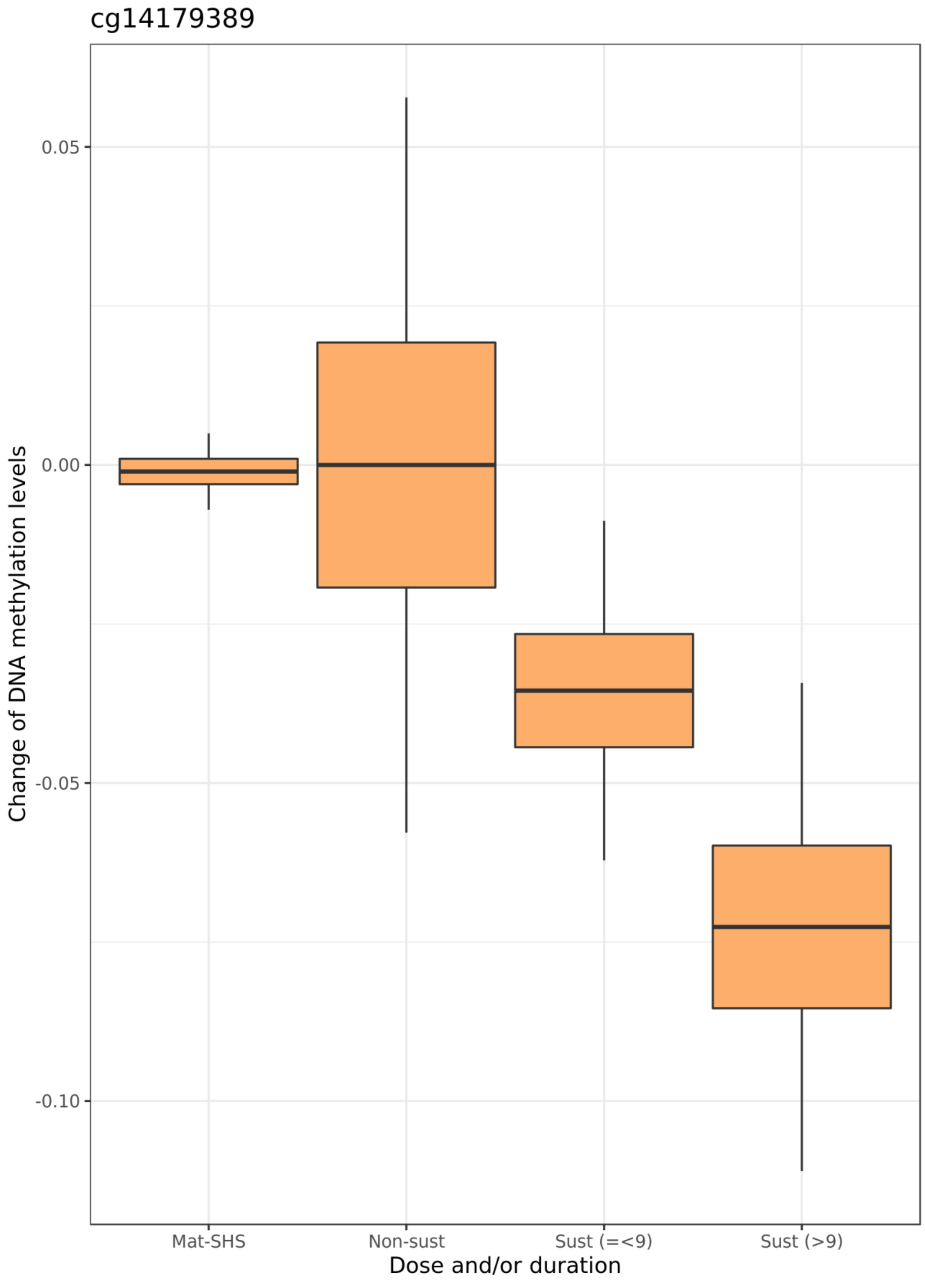
cg14179389
0.05
0.00
-0.05
-0.10
Change of DNA methylation levels
Mat-SHS
Non-sust
Sust (=<9)
Sust (>9)
Dose and/or duration

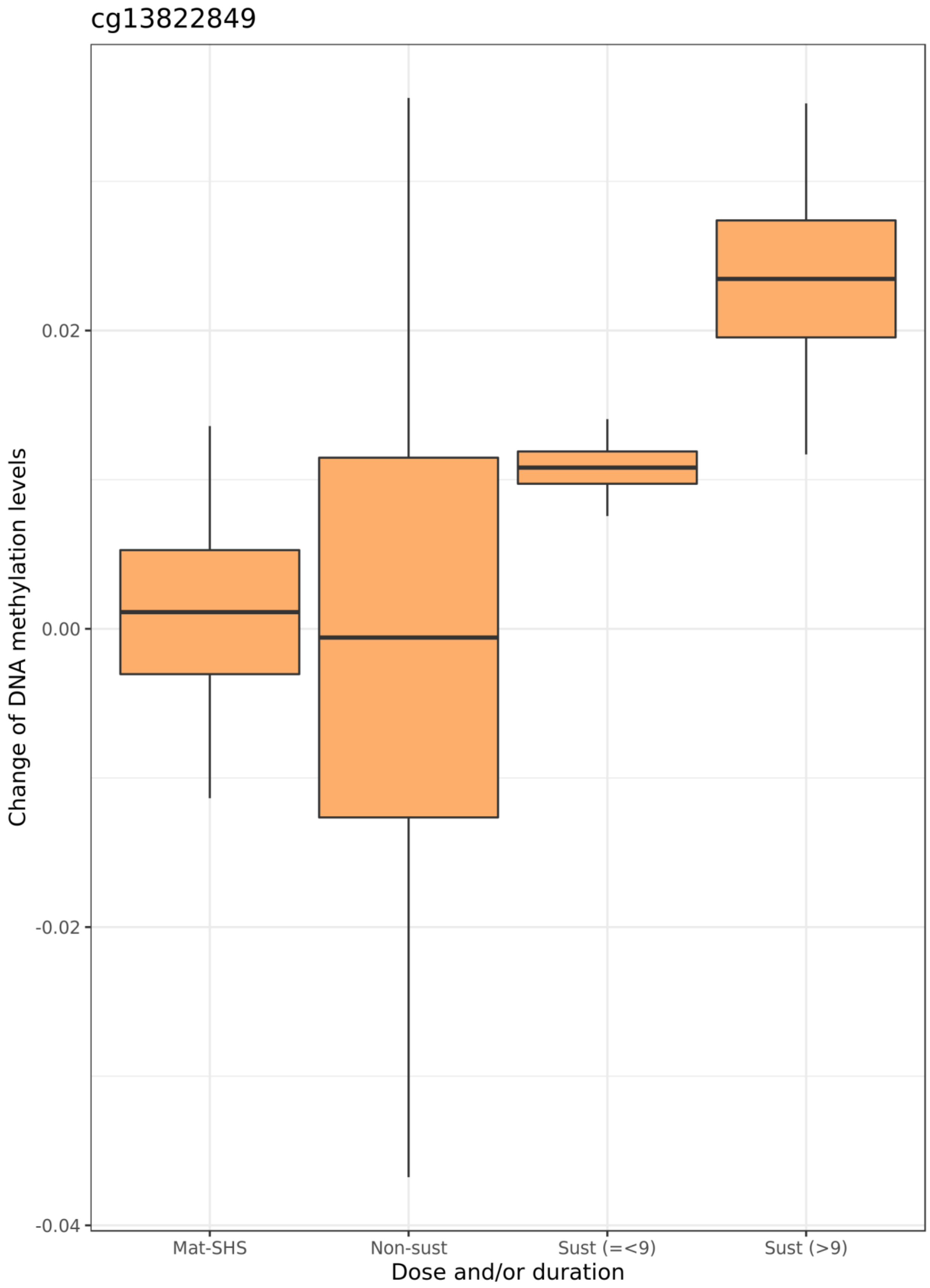
cg13822849
Change of DNA methylation levels
0.02
0.00
-0.02
-0.04
Mat-SHS
Non-sust
Sust (=<9)
Sust (>9)
Dose and/or duration

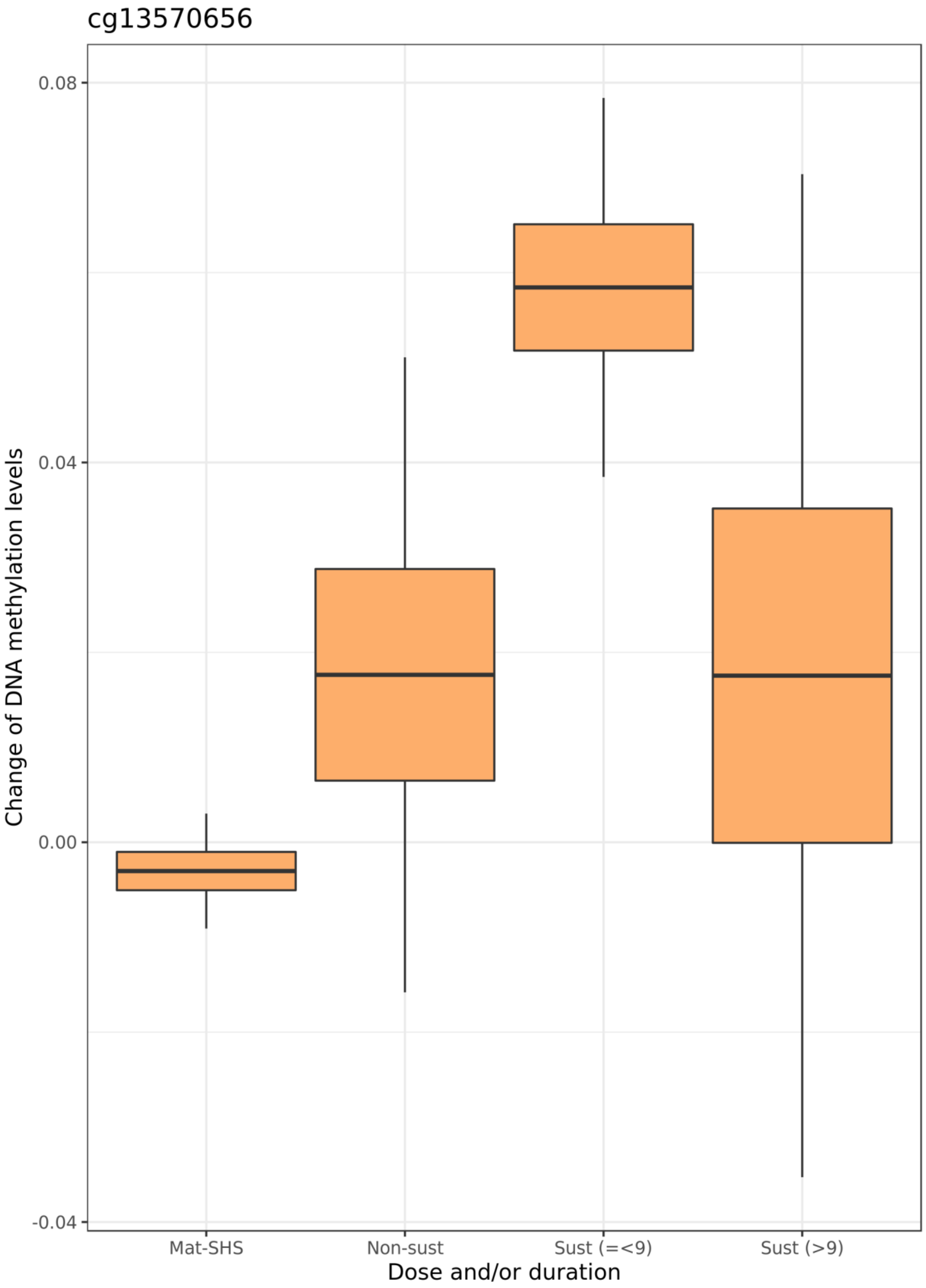
cg13570656
0.08
0.04
0.00
-0.04
Change of DNA methylation levels
Mat-SHS
Non-sust
Sust (=<9)
Sust (>9)
Dose and/or duration

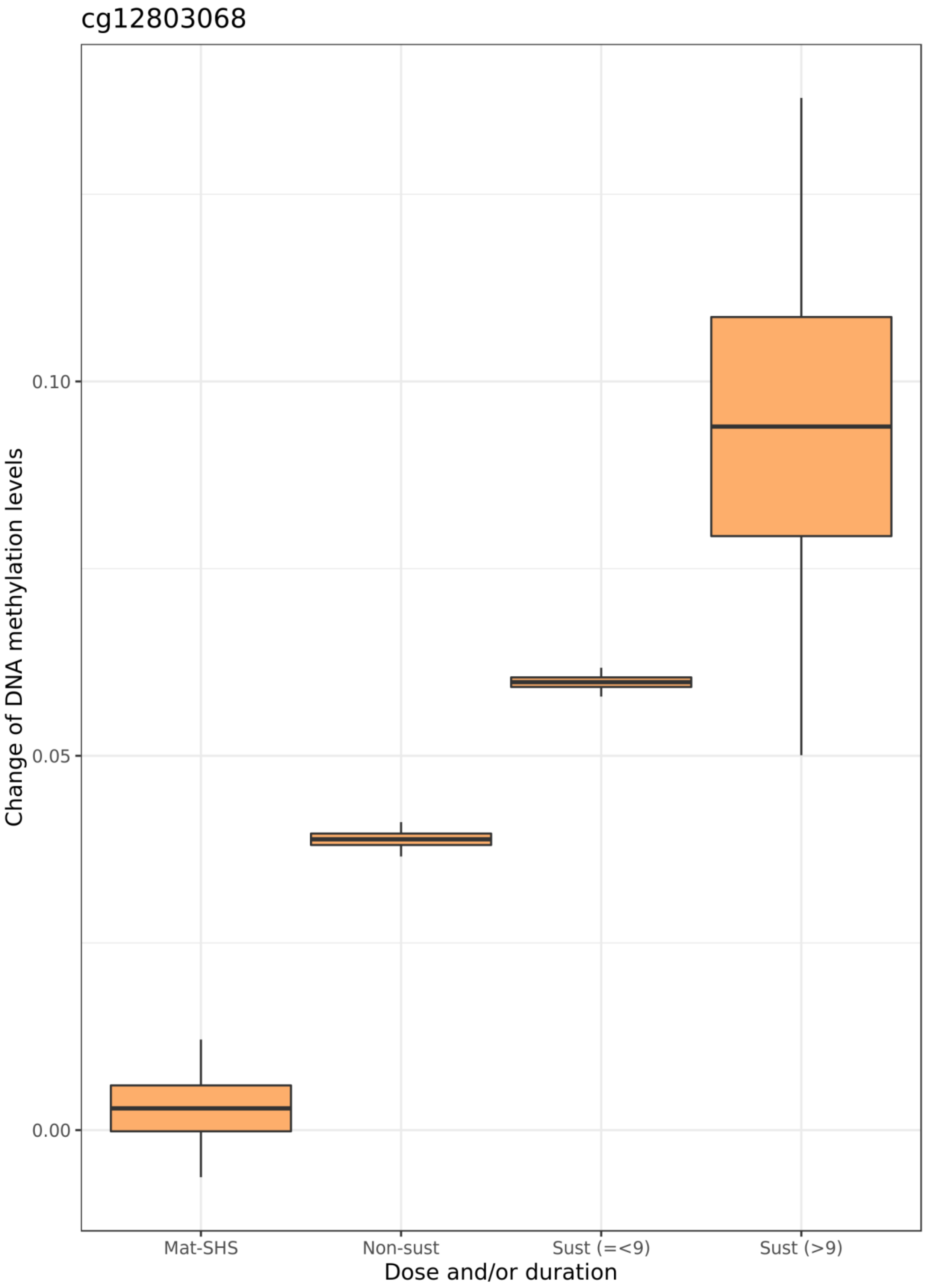
cg12803068
Change of DNA methylation levels
0.10
0.05
0.00
Mat-SHS
Non-sust
Sust (=<9)
Sust (>9)
Dose and/or duration

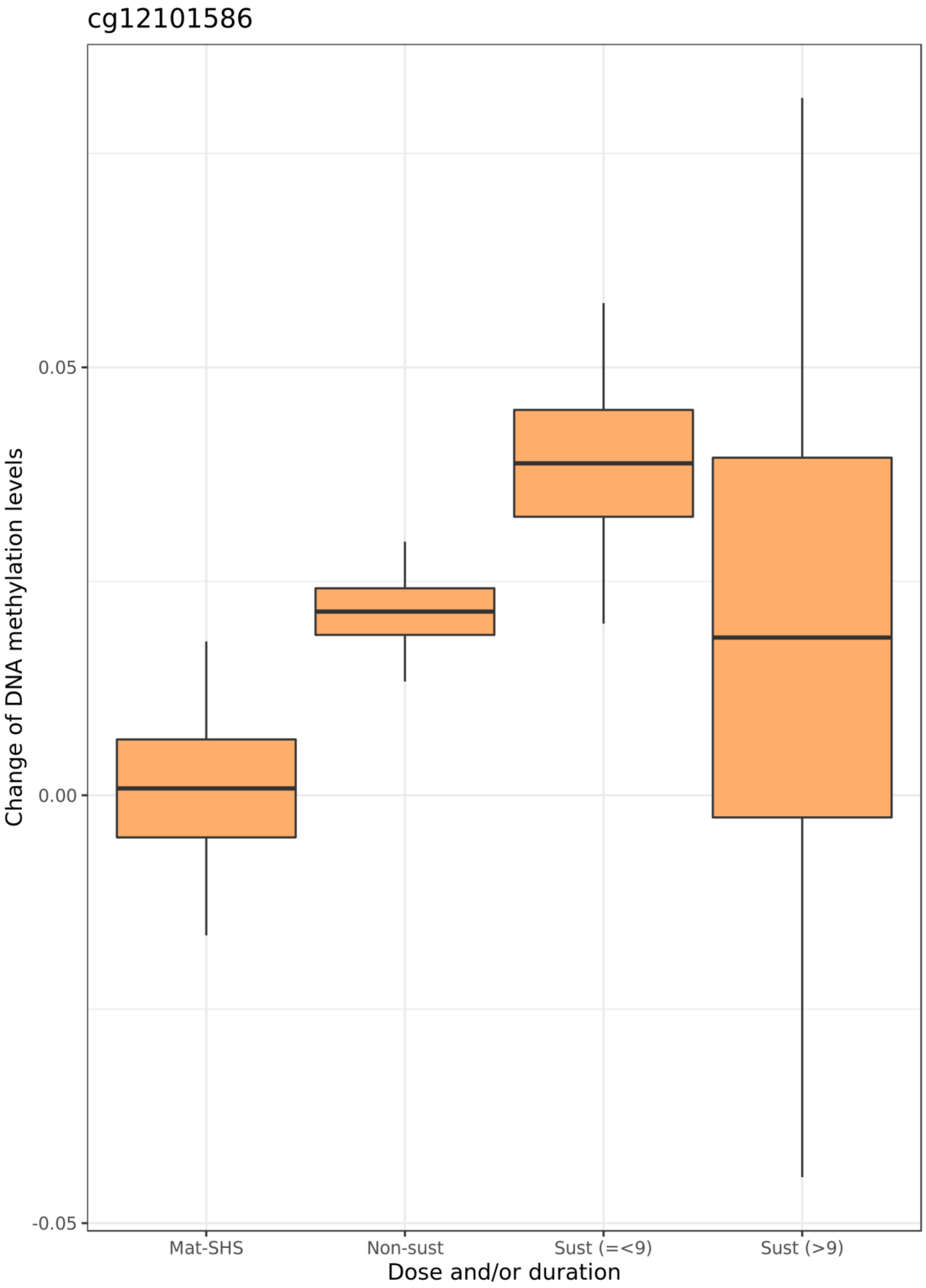
cg12101586
Change of DNA methylation levels
0.05
0.00
-0.05
Mat-SHS
Non-sust
Sust (=<9)
Sust (>9)
Dose and/or duration

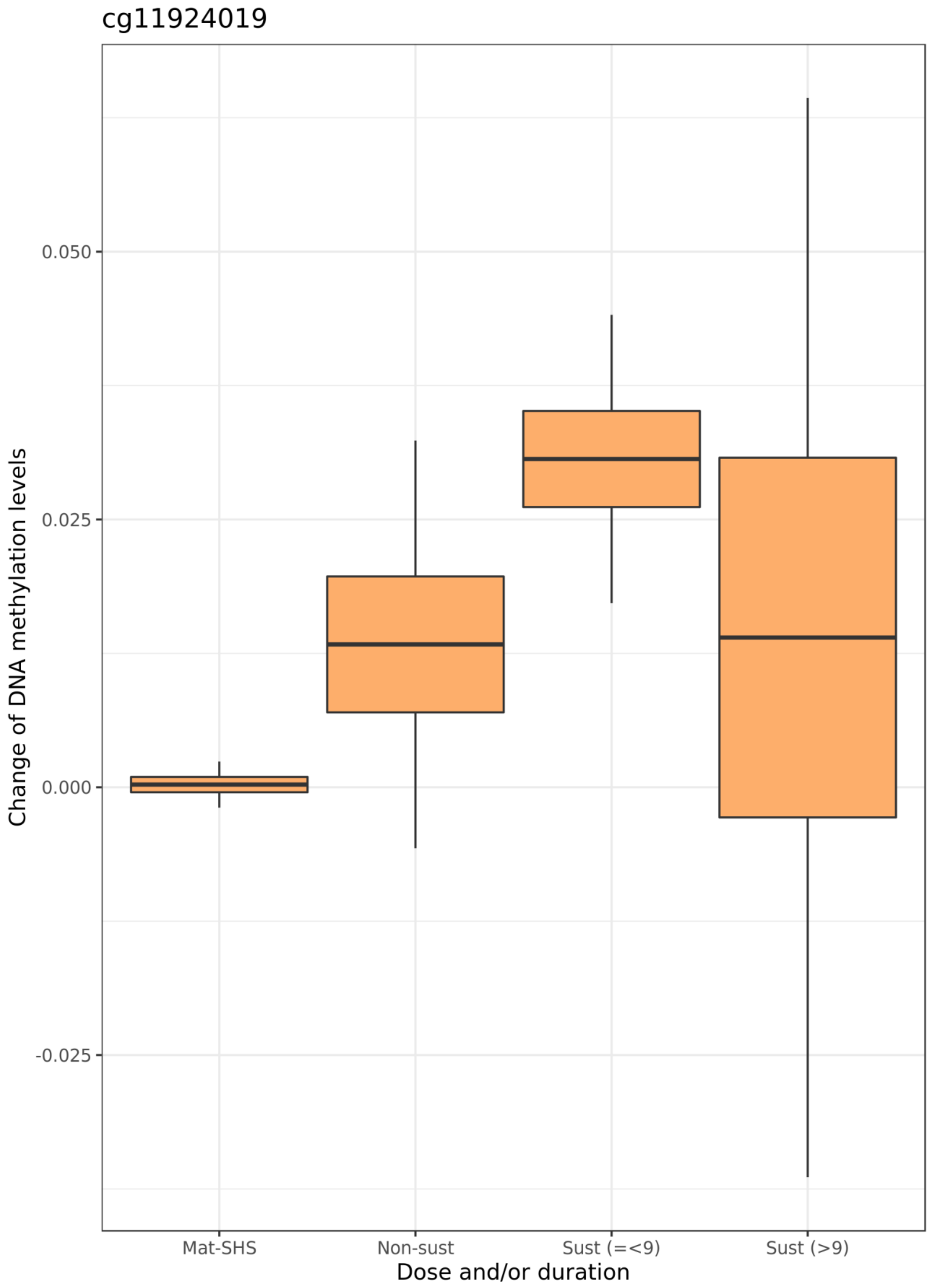
cg11924019
0.050
0.025
0.000
-0.025
Change of DNA methylation levels
Mat-SHS
Non-sust
Sust (=<9)
Sust (>9)
Dose and/or duration

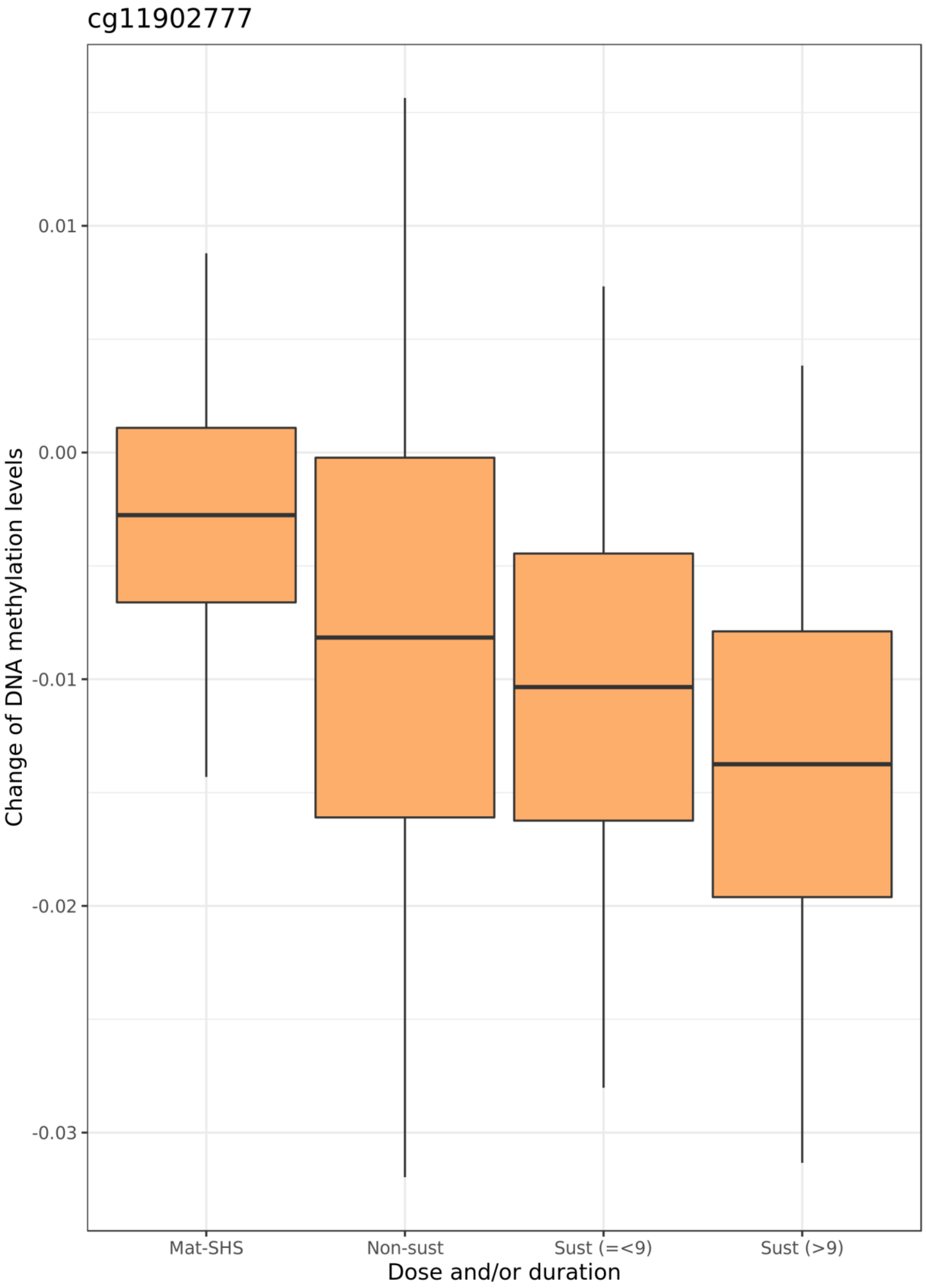
cg11902777
Change of DNA methylation levels
0.01
0.00
-0.01
-0.02
-0.03
Mat-SHS
Non-sust
Sust (=<9)
Sust (>9)
Dose and/or duration

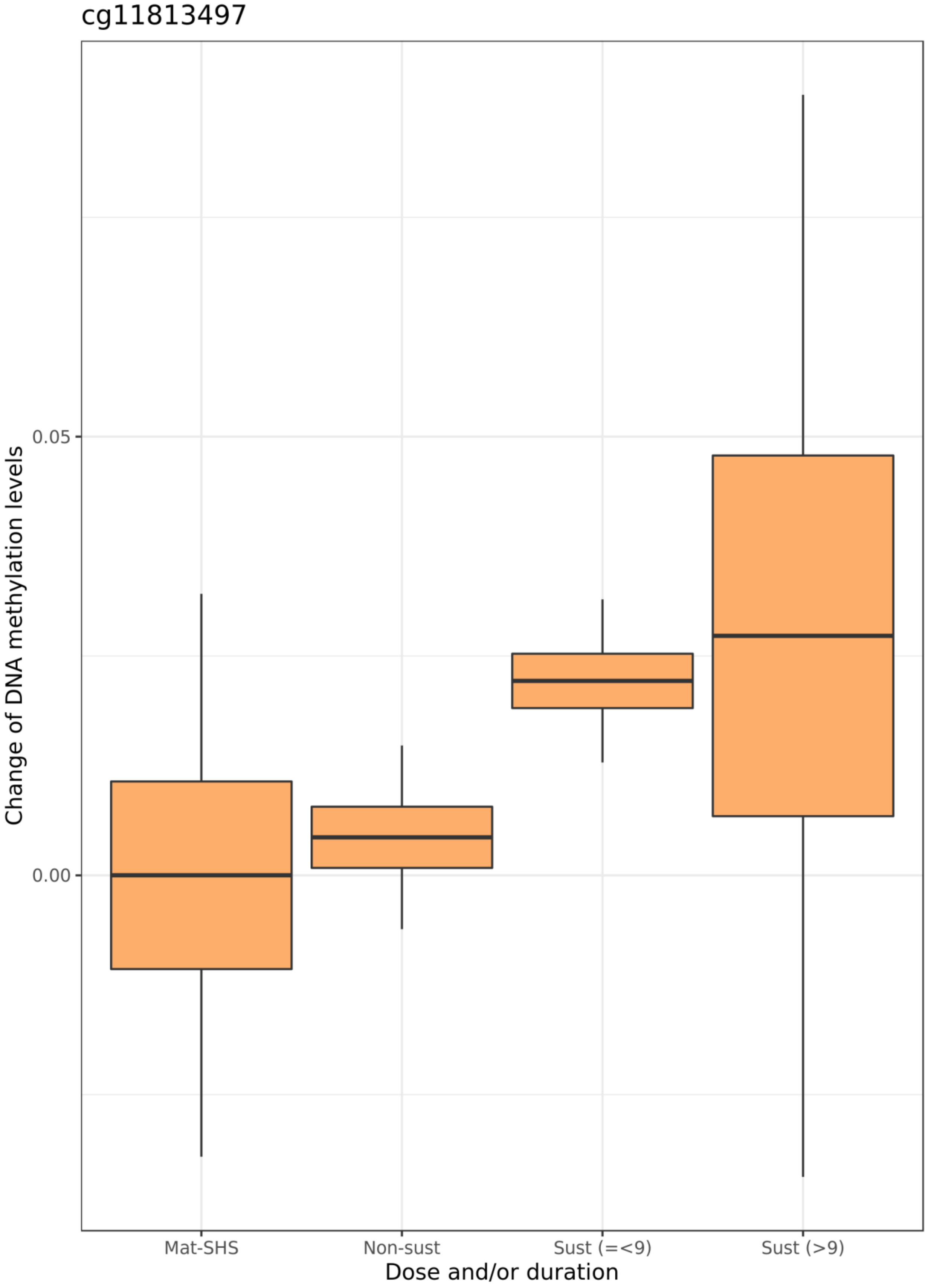

cg11813497
Change of DNA methylation levels
0.05
0.00
Mat-SHS
Non-sust
Sust (=<9)
Sust (>9)
Dose and/or duration

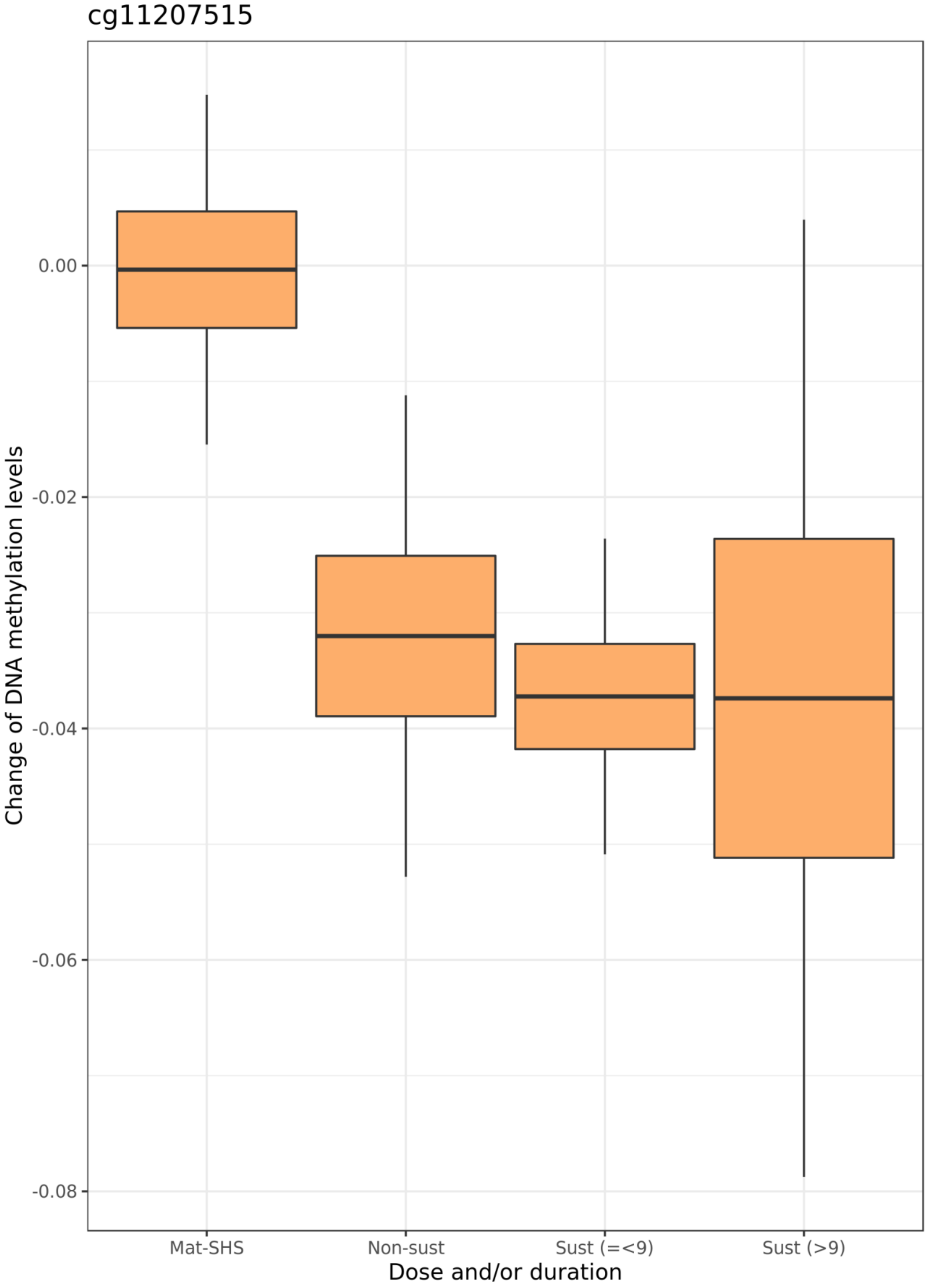
cg11207515
Change of DNA methylation levels
0.00
-0.02
-0.04
-0.06
-0.08
Mat-SHS
Non-sust
Sust (=<9)
Sust (>9)
Dose and/or duration

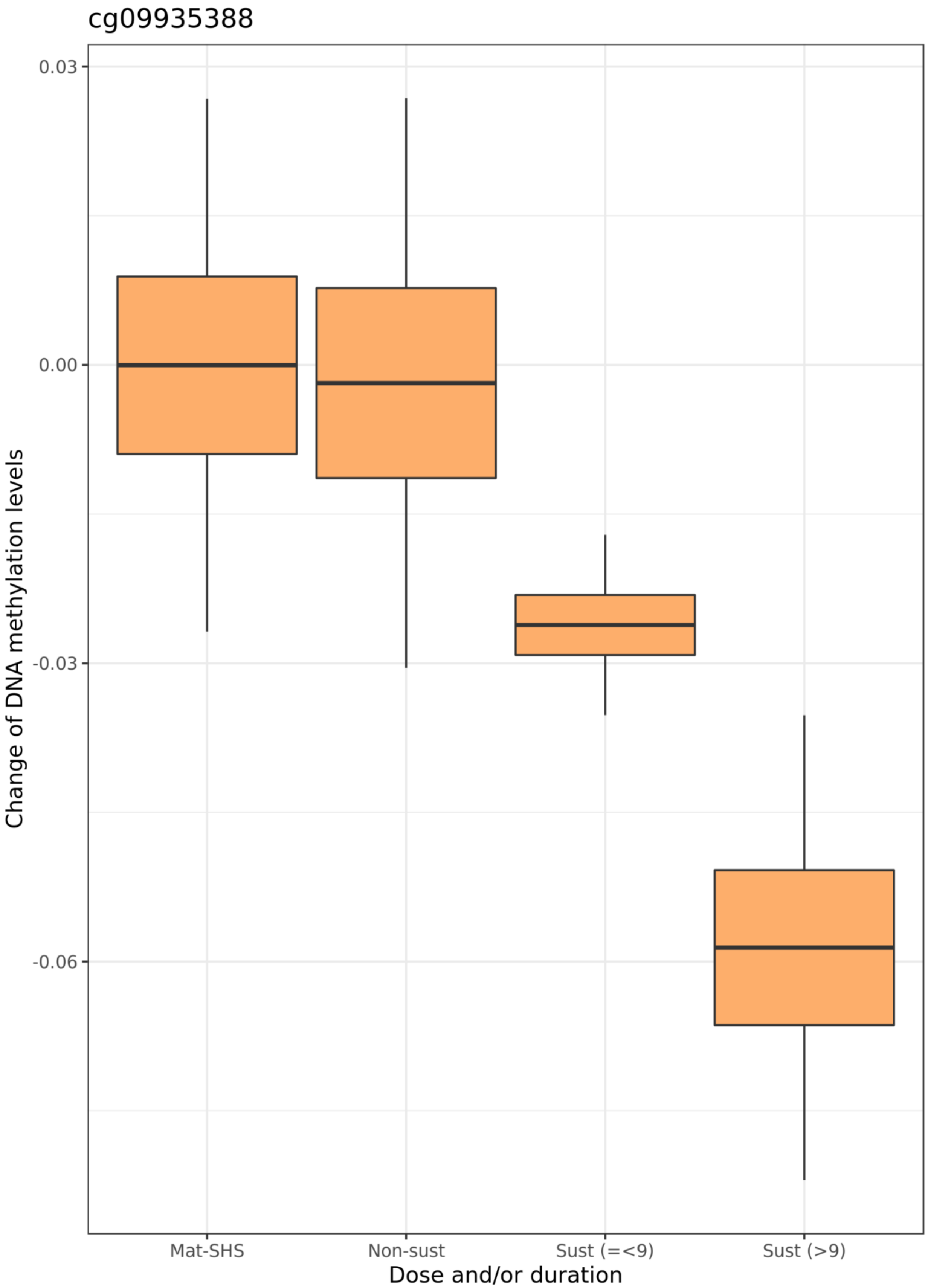

cg09935388
0.03
0.00
-0.03
-0.06
Change of DNA methylation levels
Mat-SHS
Non-sust
Sust (=<9)
Sust (>9)
Dose and/or duration

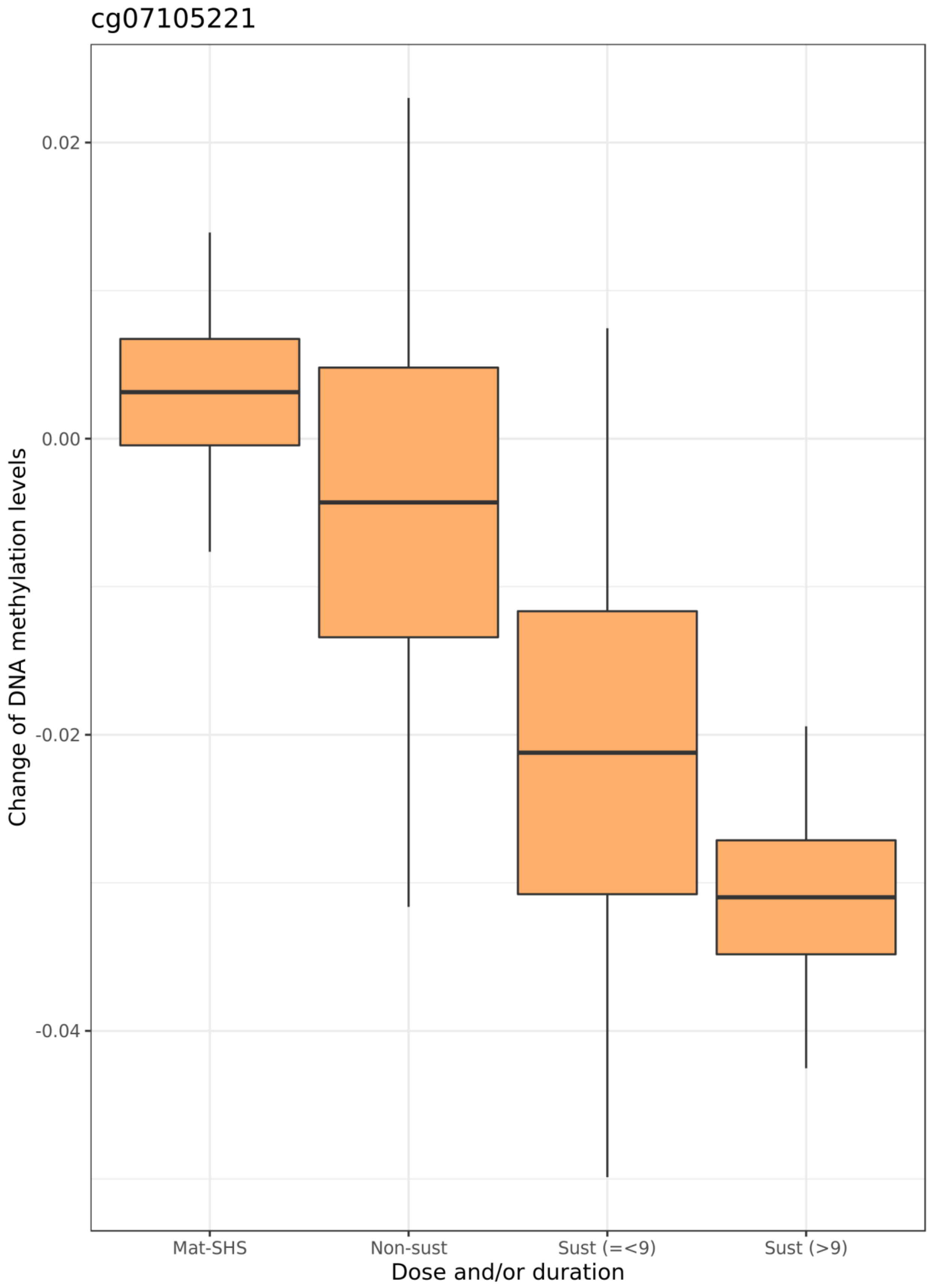
cg07105221
0.02
0.00
-0.02
-0.04
Change of DNA methylation levels
Mat-SHS
Non-sust
Sust (=<9)
Sust (>9)
Dose and/or duration

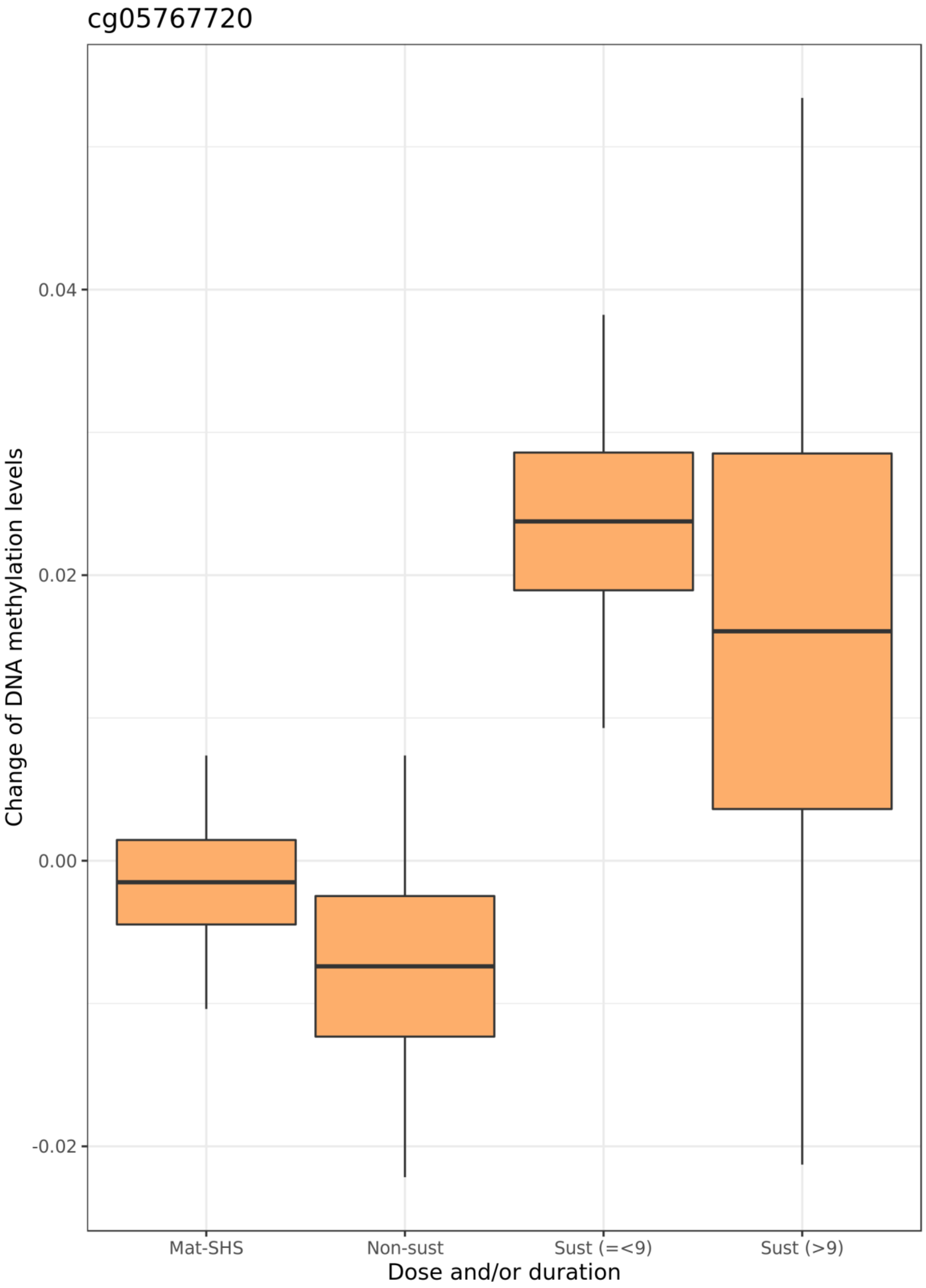

cg05767720
Change of DNA methylation levels
0.04
0.02
0.00
-0.02
Mat-SHS
Non-sust
Sust (=<9)
Sust (>9)
Dose and/or duration

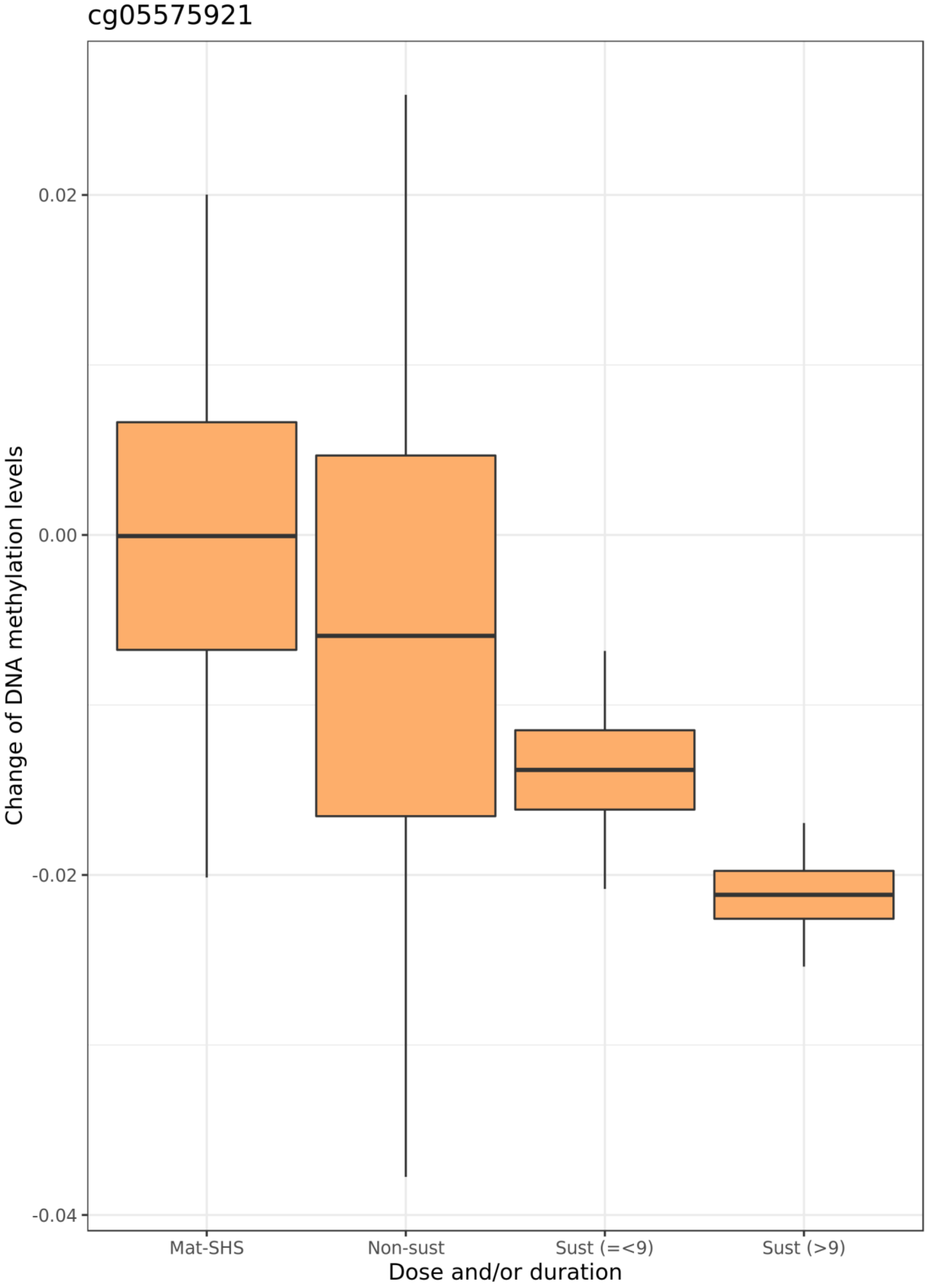
cg05575921
Change of DNA methylation levels
0.02
0.00
-0.02
-0.04
Mat-SHS
Non-sust
Sust (=<9)
Sust (>9)
Dose and/or duration

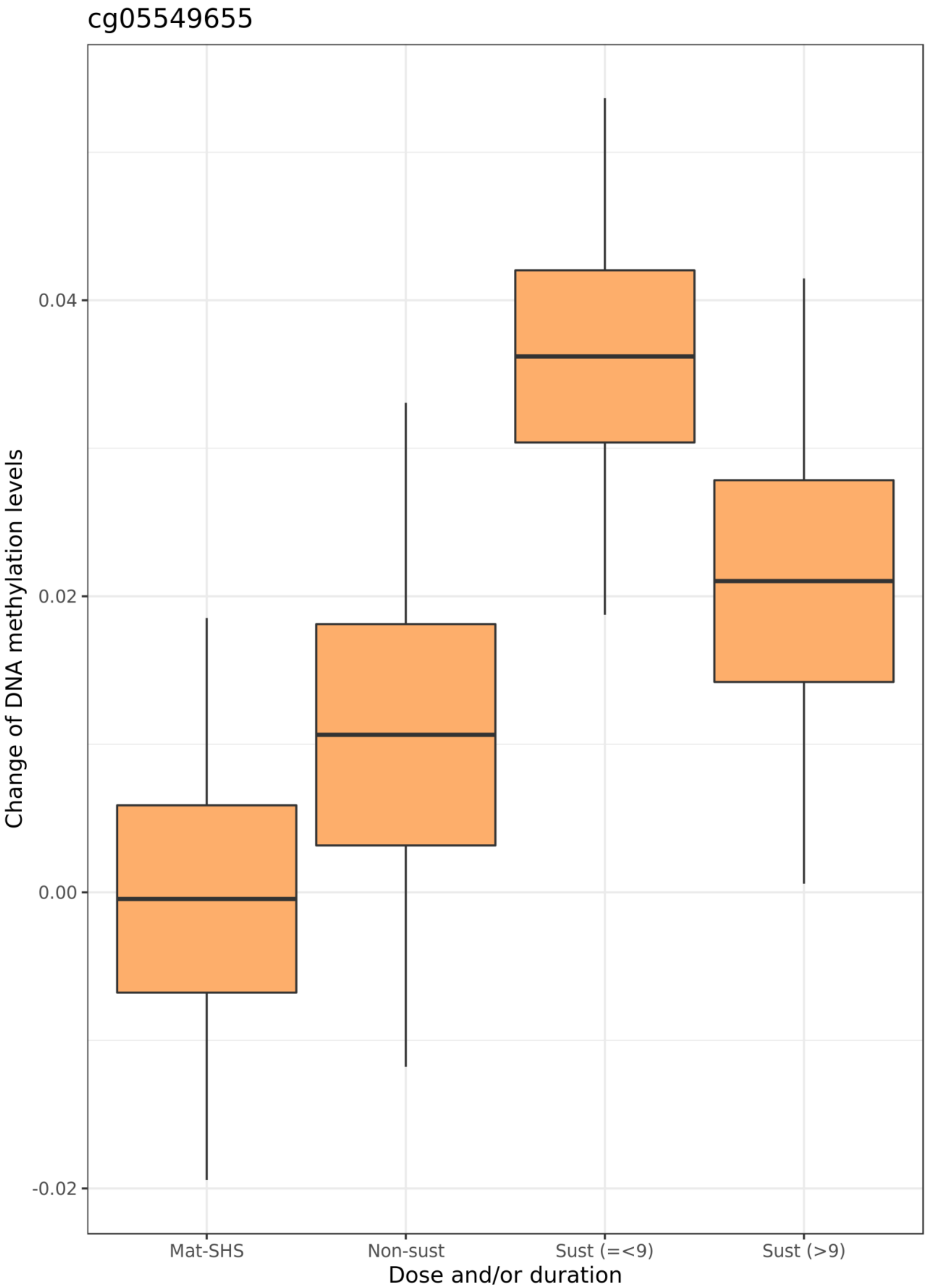

cg05549655
Change of DNA methylation levels
0.04
0.02
0.00
-0.02
Mat-SHS
Non-sust
Sust (=<9)
Sust (>9)
Dose and/or duration

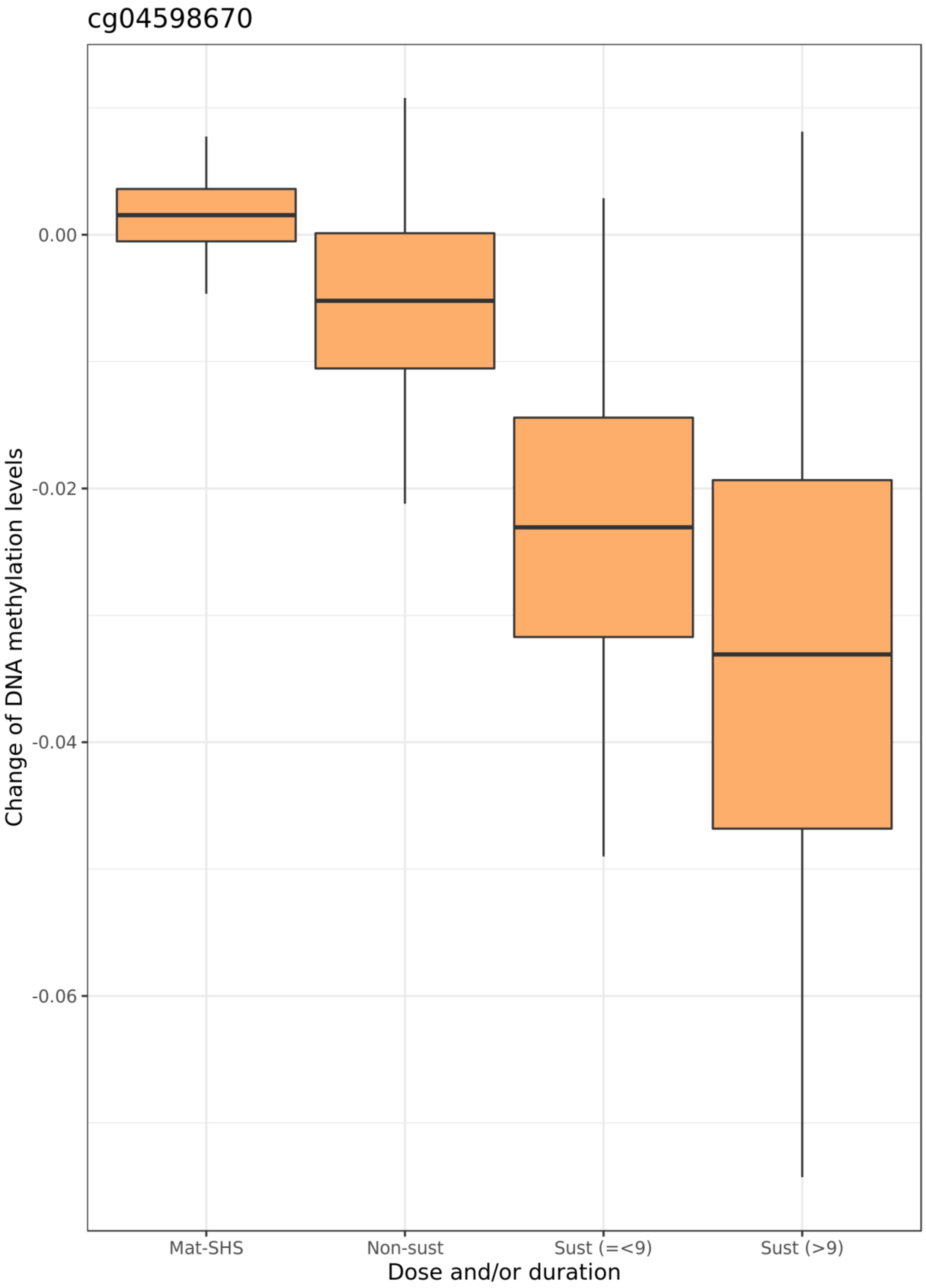
cg04598670
Change of DNA methylation levels
0.00
-0.02
-0.04
-0.06
Mat-SHS
Non-sust
Sust (=<9)
Sust (>9)
Dose and/or duration

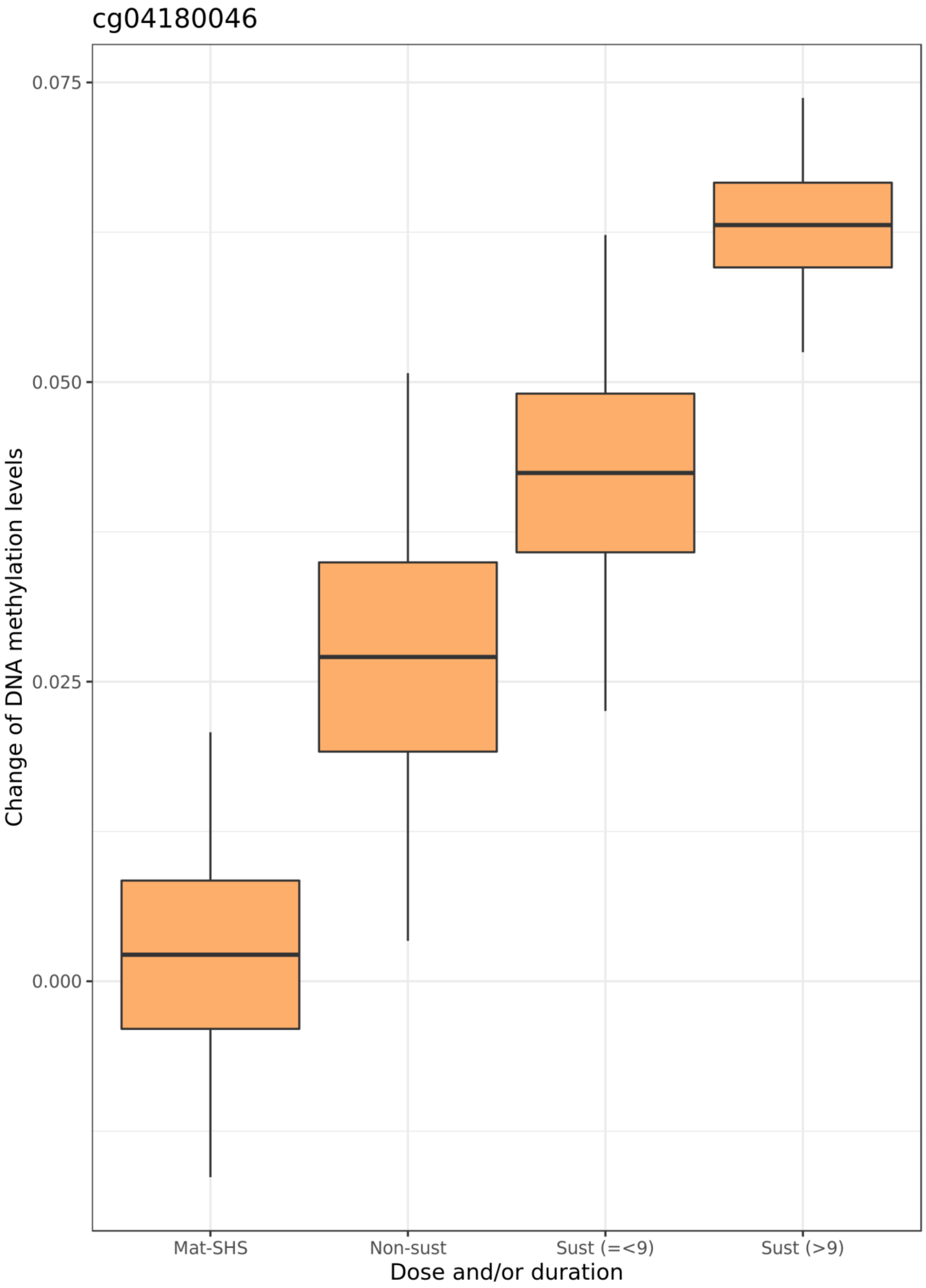
cg04180046
Change of DNA methylation levels
0.075
0.050
0.025
0.000
Mat-SHS
Non-sust
Sust (=<9)
Sust (>9)
Dose and/or duration

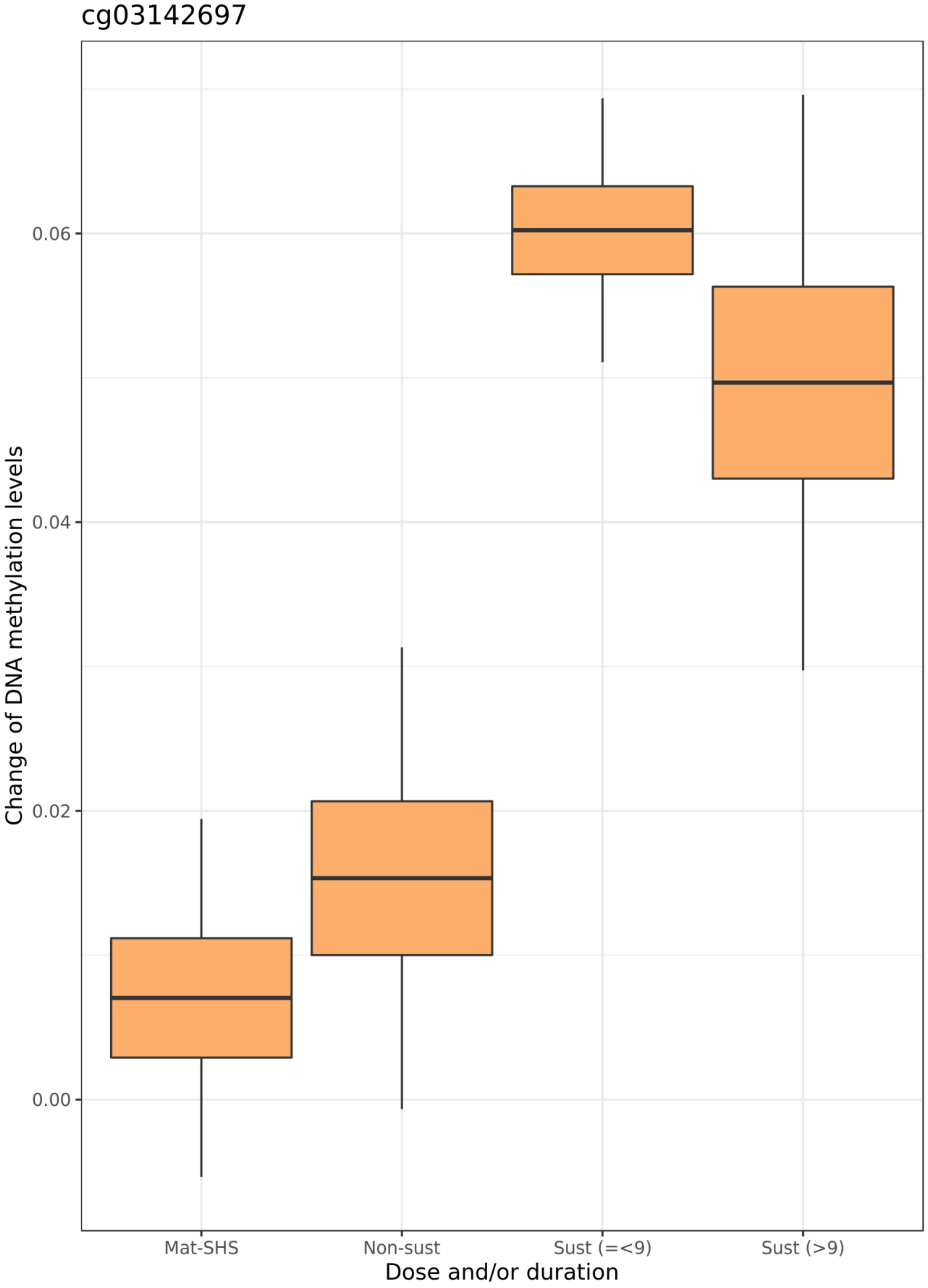
cg03142697
0.06
0.04
0.02
0.00
Change of DNA methylation levels
Mat-SHS
Non-sust
Sust (=<9)
Sust (>9)
Dose and/or duration

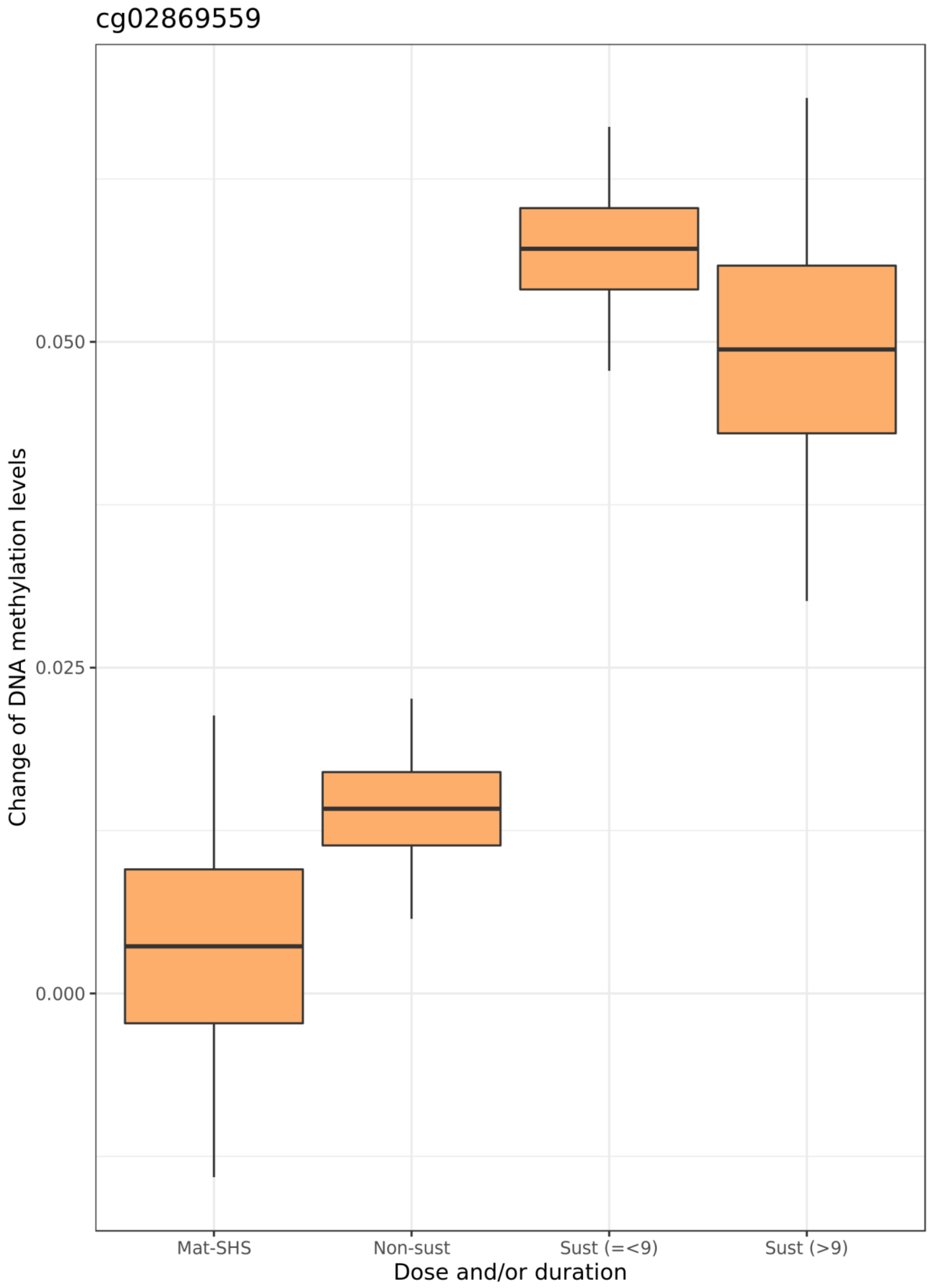
cg02869559
Change of DNA methylation levels
0.050
0.025
0.000
Mat-SHS
Non-sust
Sust (=<9)
Sust (>9)
Dose and/or duration

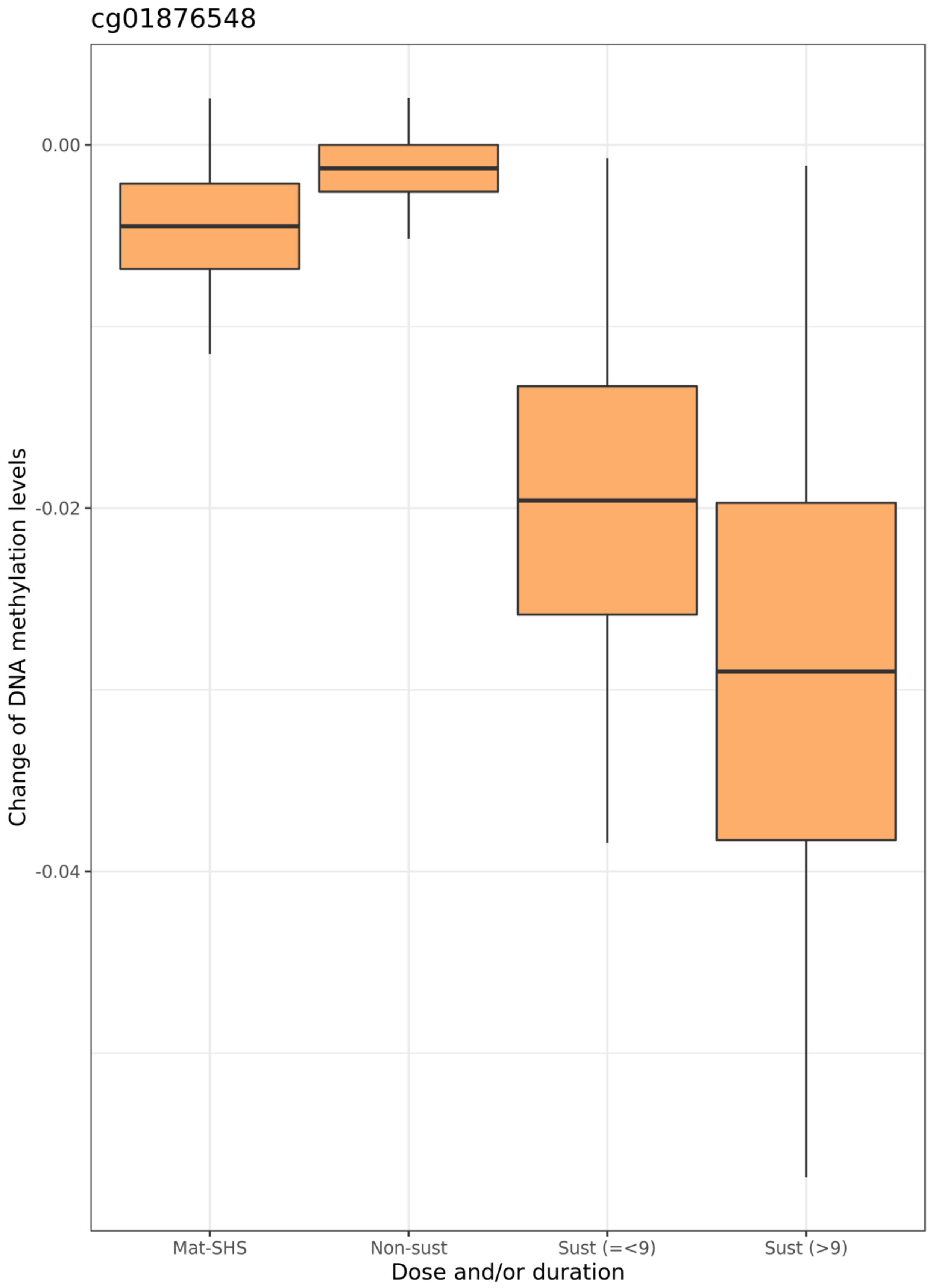
cg01876548
0.00
-0.02
-0.04
Change of DNA methylation levels
Mat-SHS
Non-sust
Sust (=<9)
Sust (>9)
Dose and/or duration

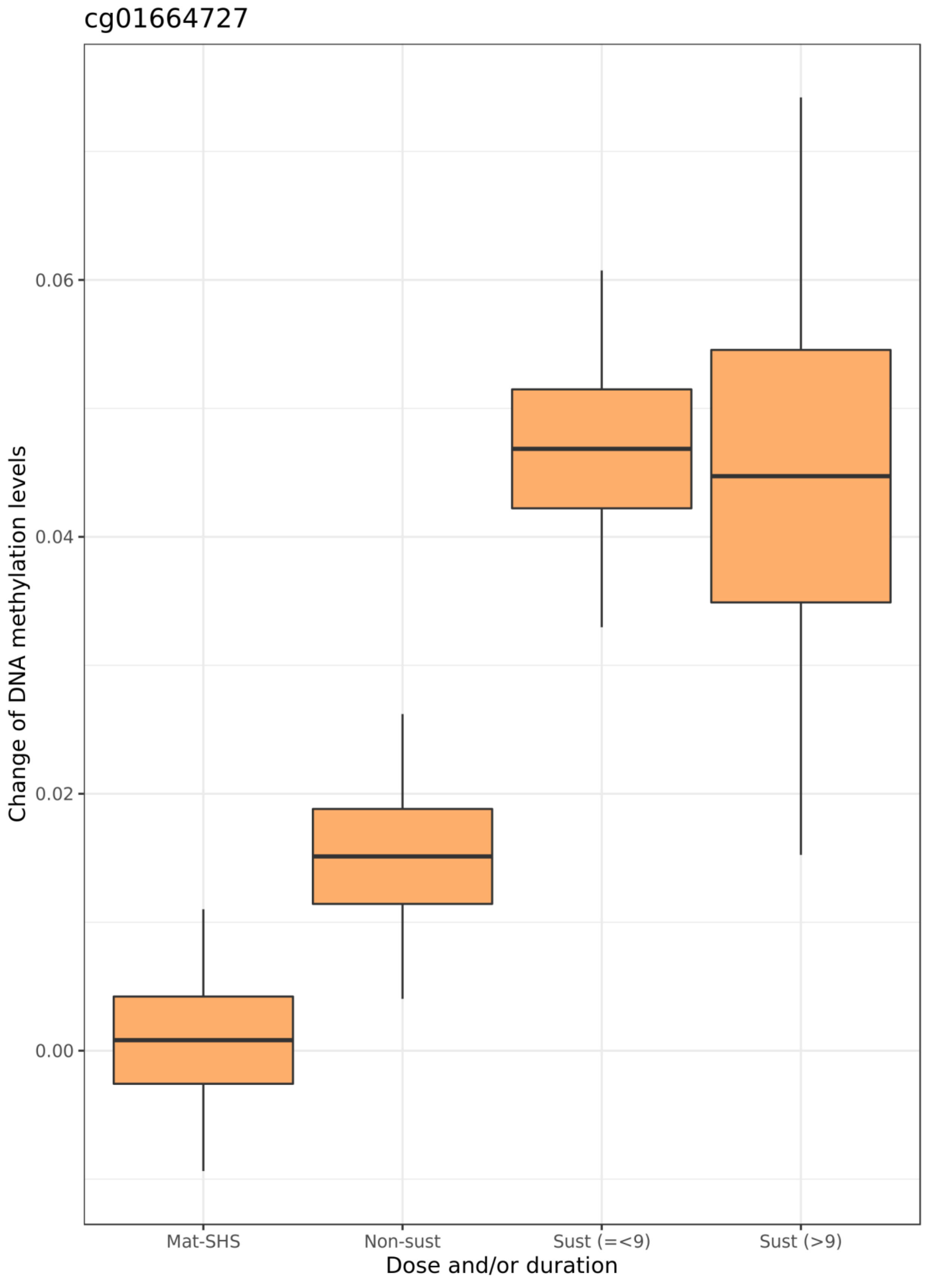
cg01664727
Change of DNA methylation levels
0.06
0.04
0.02
0.00
Mat-SHS
Non-sust
Sust (=<9)
Sust (>9)
Dose and/or duration

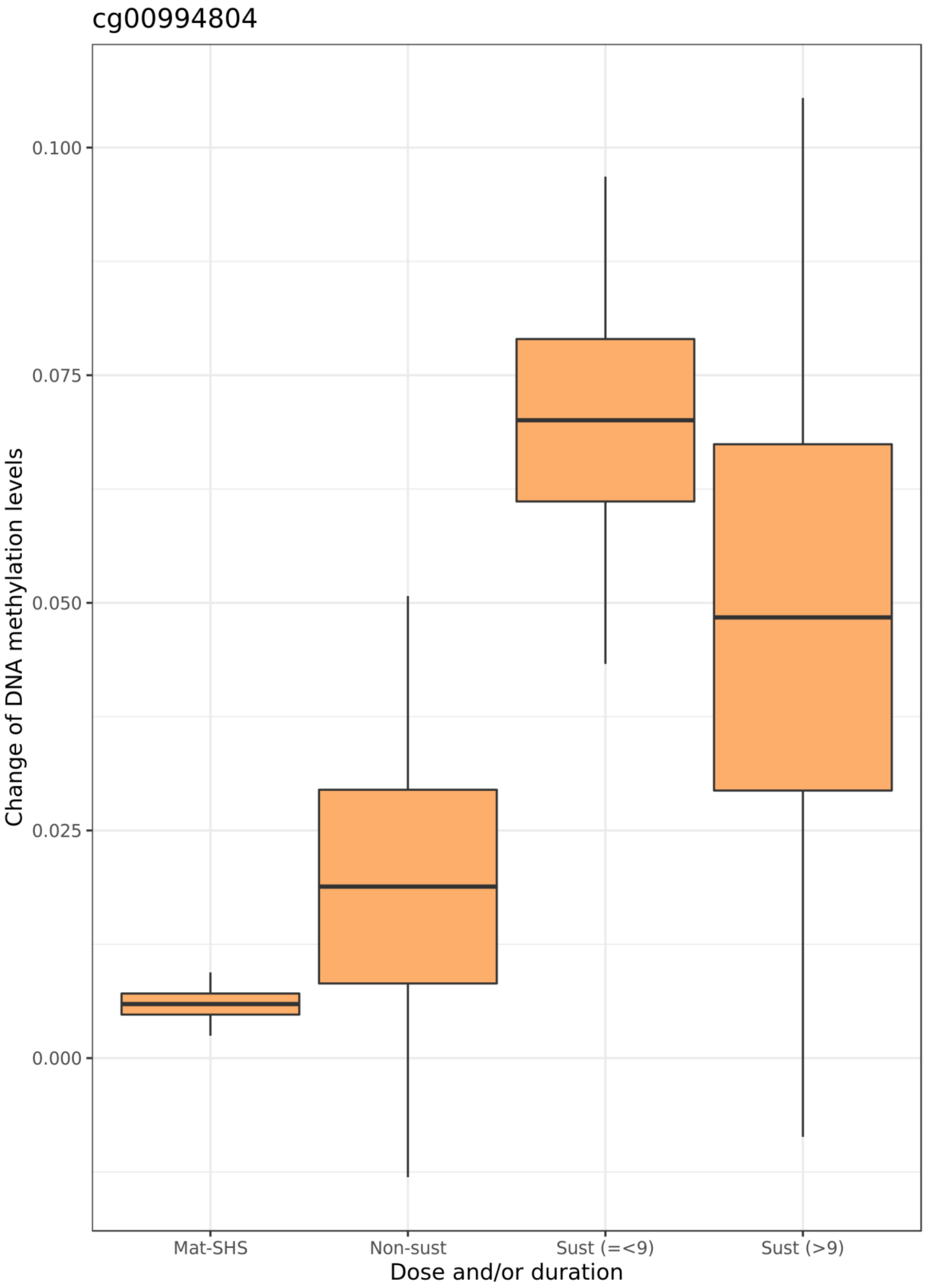
cg00994804
Change of DNA methylation levels
0.100
0.075
0.050
0.025
0.000
Mat-SHS
Non-sust
Sust (=<9)
Sust (>9)
Dose and/or duration

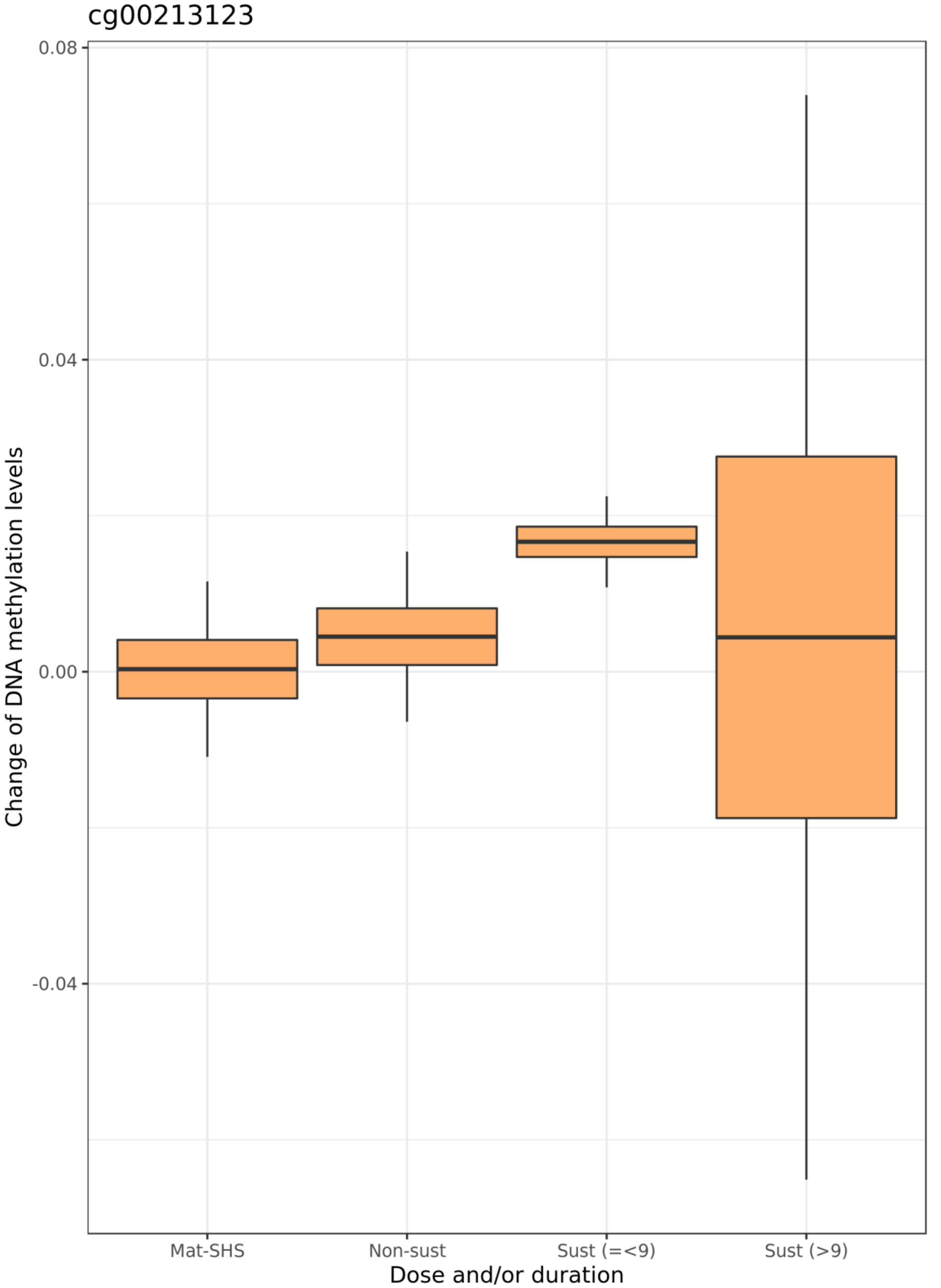
cg00213123
0.08
0.04
0.00
-0.04
Change of DNA methylation levels
Mat-SHS
Non-sust
Sust (=<9)
Sust (>9)
Dose and/or duration

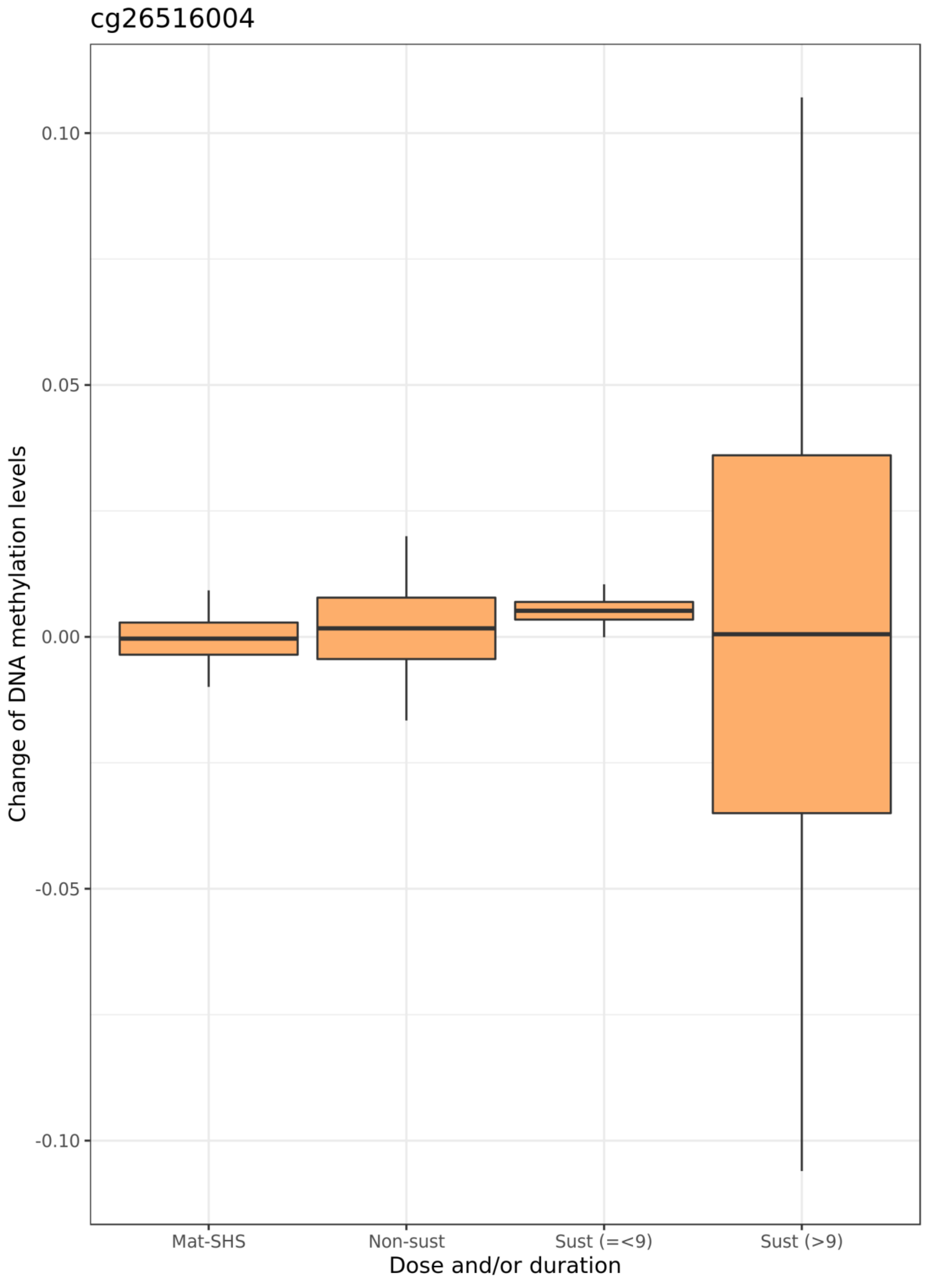
cg26516004
Change of DNA methylation levels
0.10
0.05
0.00
-0.05
-0.10
Mat-SHS
Non-sust
Sust (=<9)
Sust (>9)
Dose and/or duration

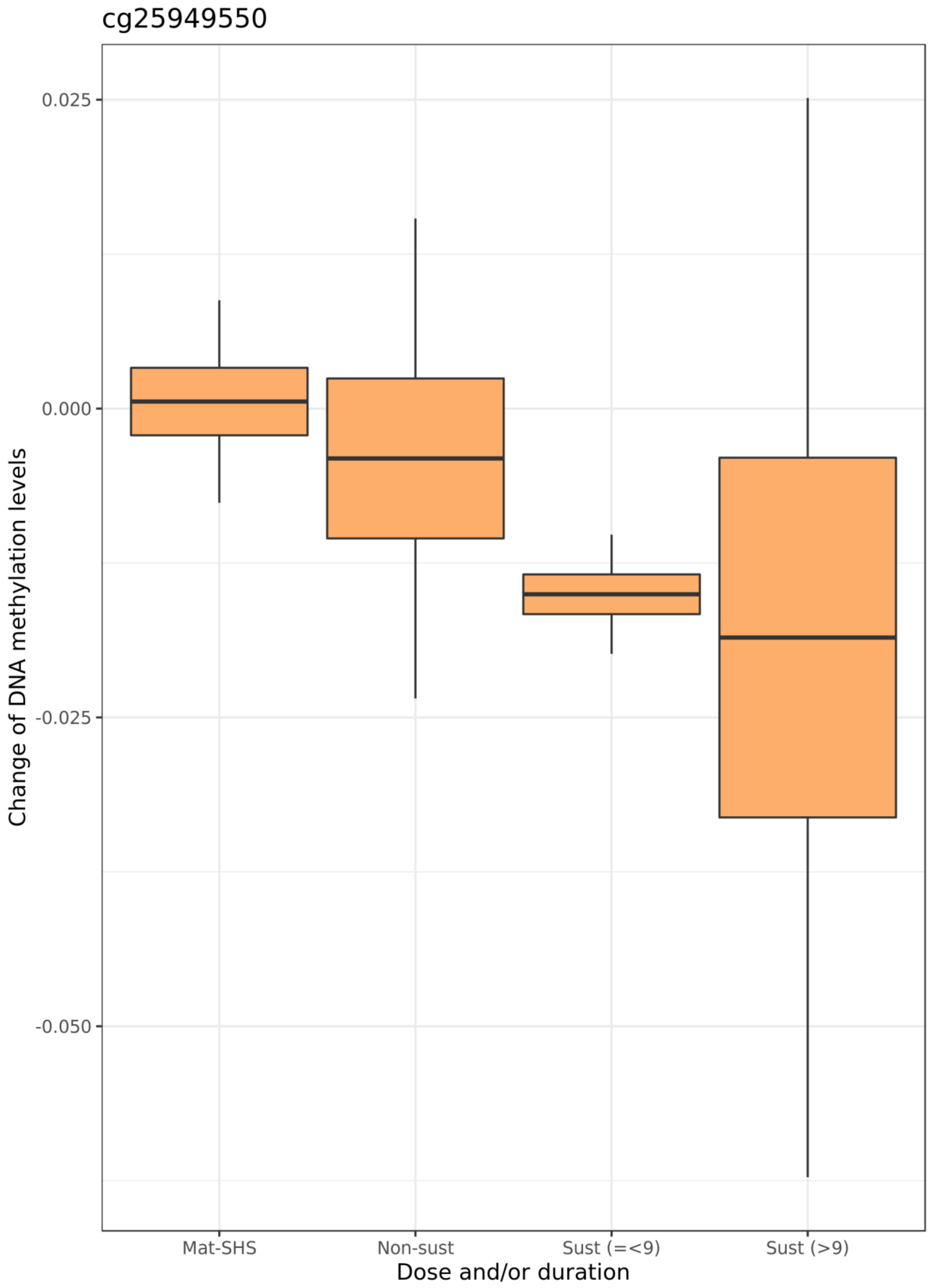
cg25949550
0.025
0.000
-0.025
-0.050
Change of DNA methylation levels
Mat-SHS
Non-sust
Sust (=<9)
Sust (>9)
Dose and/or duration

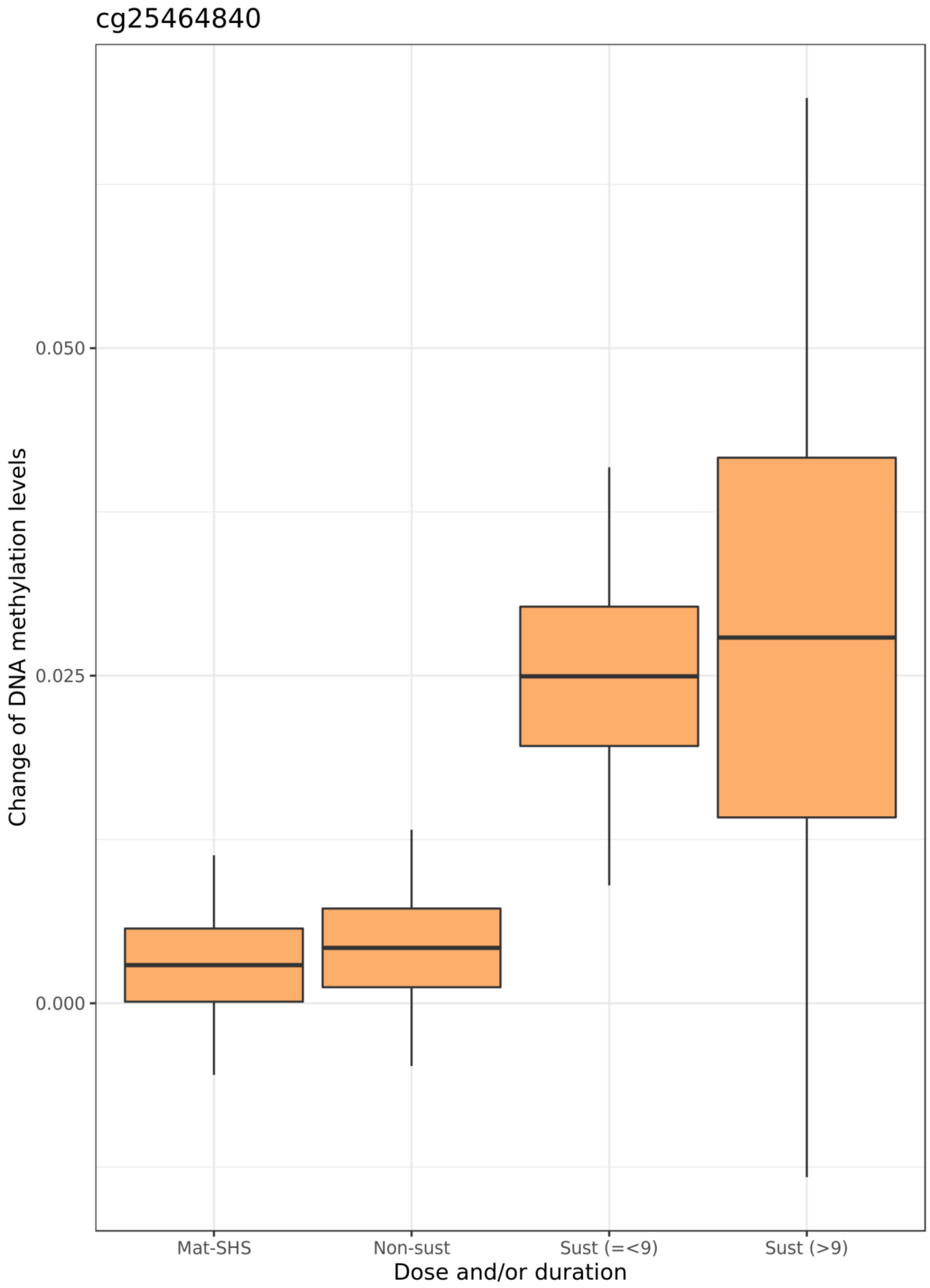

cg25464840
Change of DNA methylation levels
0.050
0.025
0.000
Mat-SHS
Non-sust
Sust (=<9)
Sust (>9)
Dose and/or duration

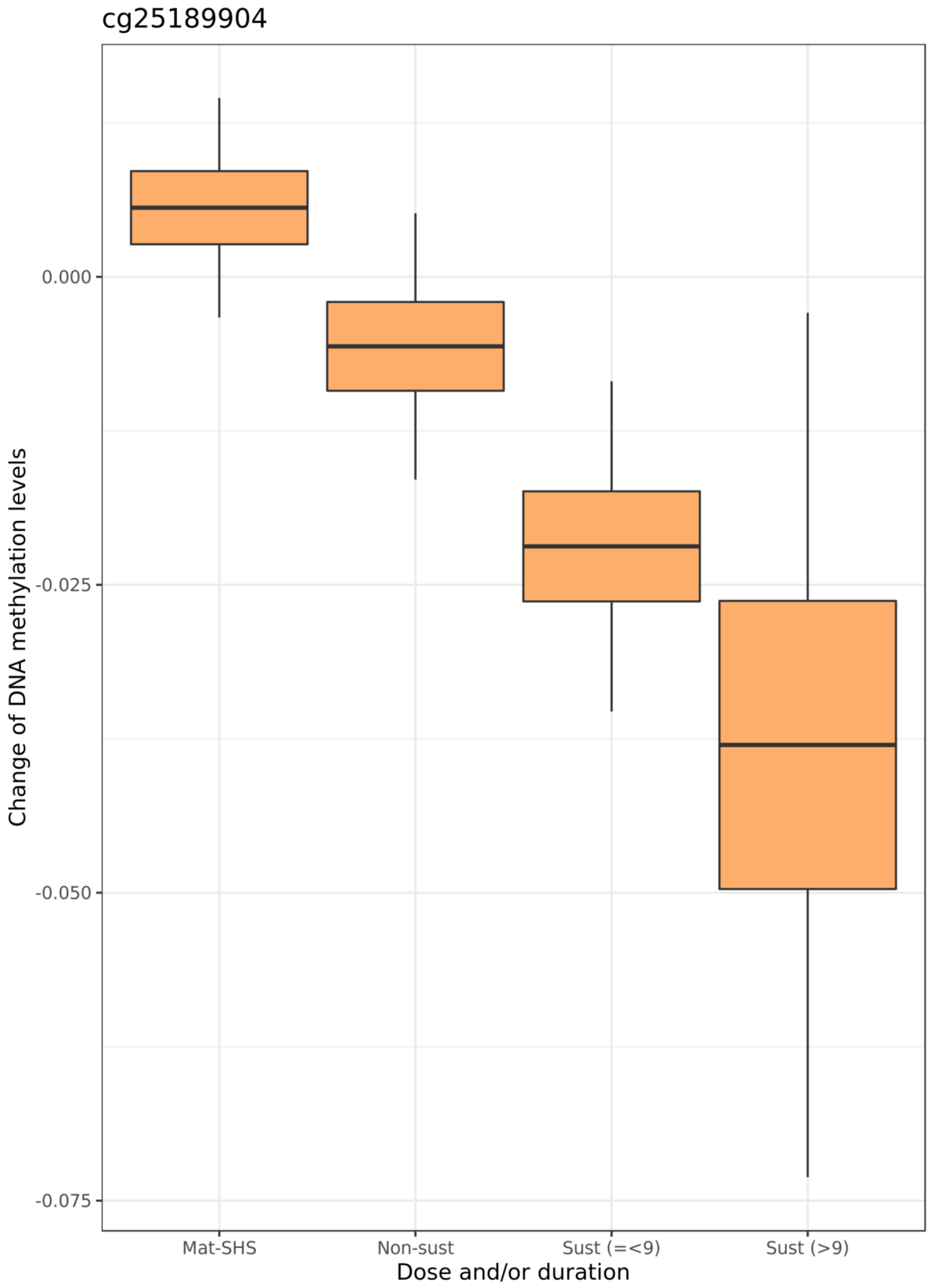
cg25189904
Change of DNA methylation levels
0.000
-0.025
-0.050
-0.075
Mat-SHS
Non-sust
Sust (=<9)
Sust (>9)
Dose and/or duration

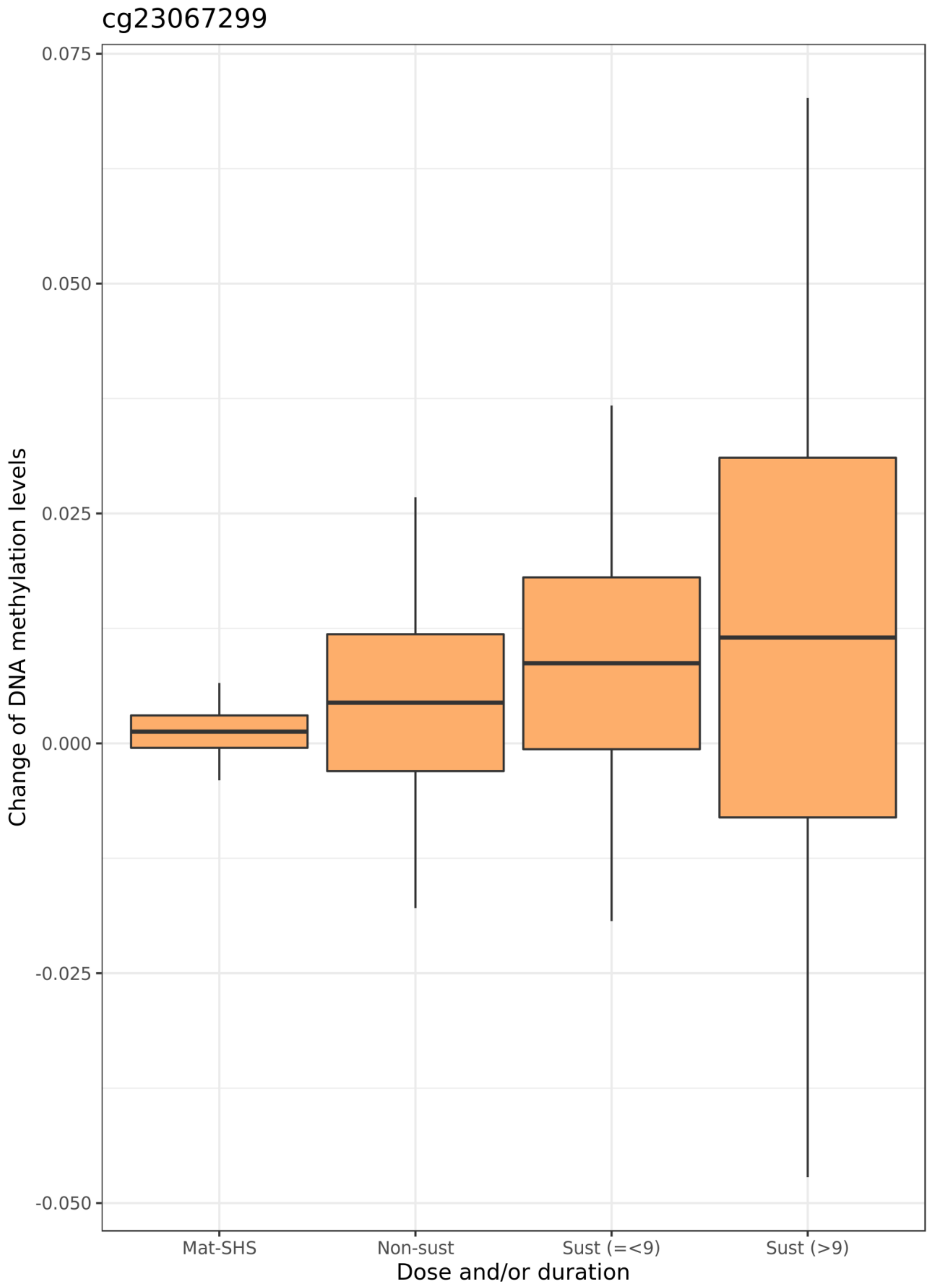
cg23067299
0.075
0.050
0.025
0.000
-0.025
-0.050
Change of DNA methylation levels
Mat-SHS
Non-sust
Sust (=<9)
Sust (>9)
Dose and/or duration

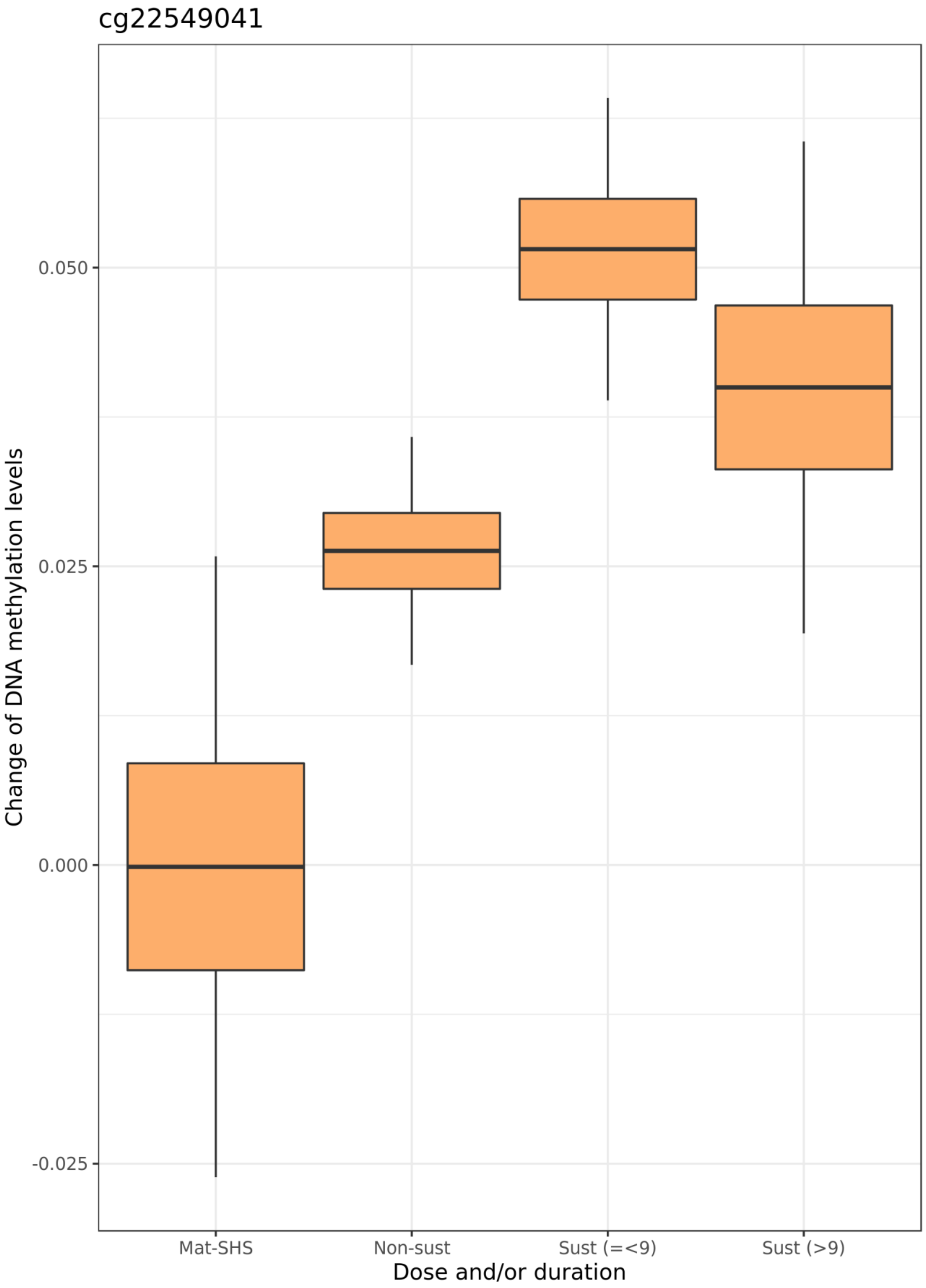
cg22549041
Change of DNA methylation levels
0.050
0.025
0.000
-0.025
Mat-SHS
Non-sust
Sust (=<9)
Sust (>9)
Dose and/or duration

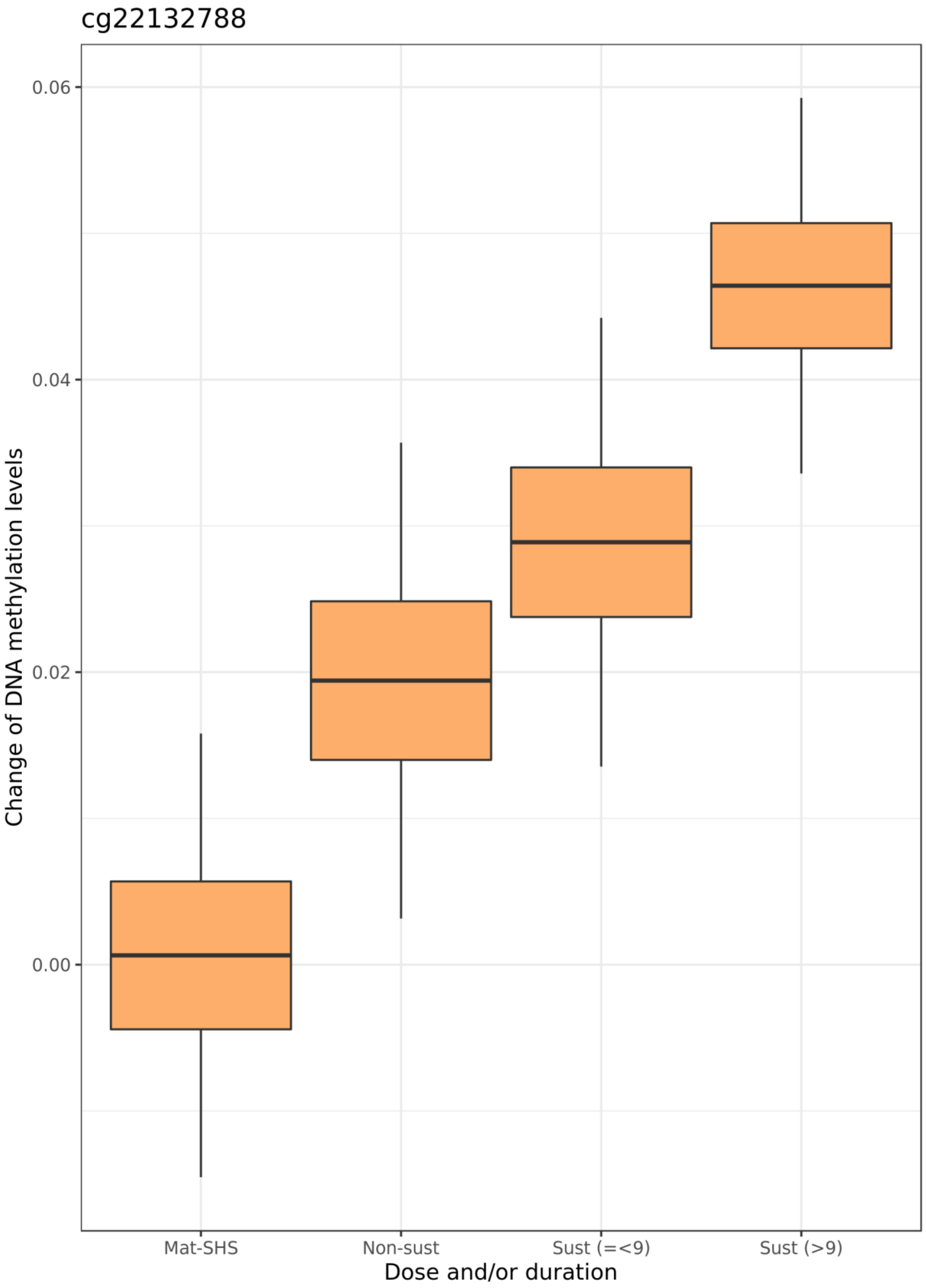

cg22132788
0.06
0.04
0.02
0.00
Change of DNA methylation levels
Mat-SHS
Non-sust
Sust (=<9)
Sust (>9)
Dose and/or duration

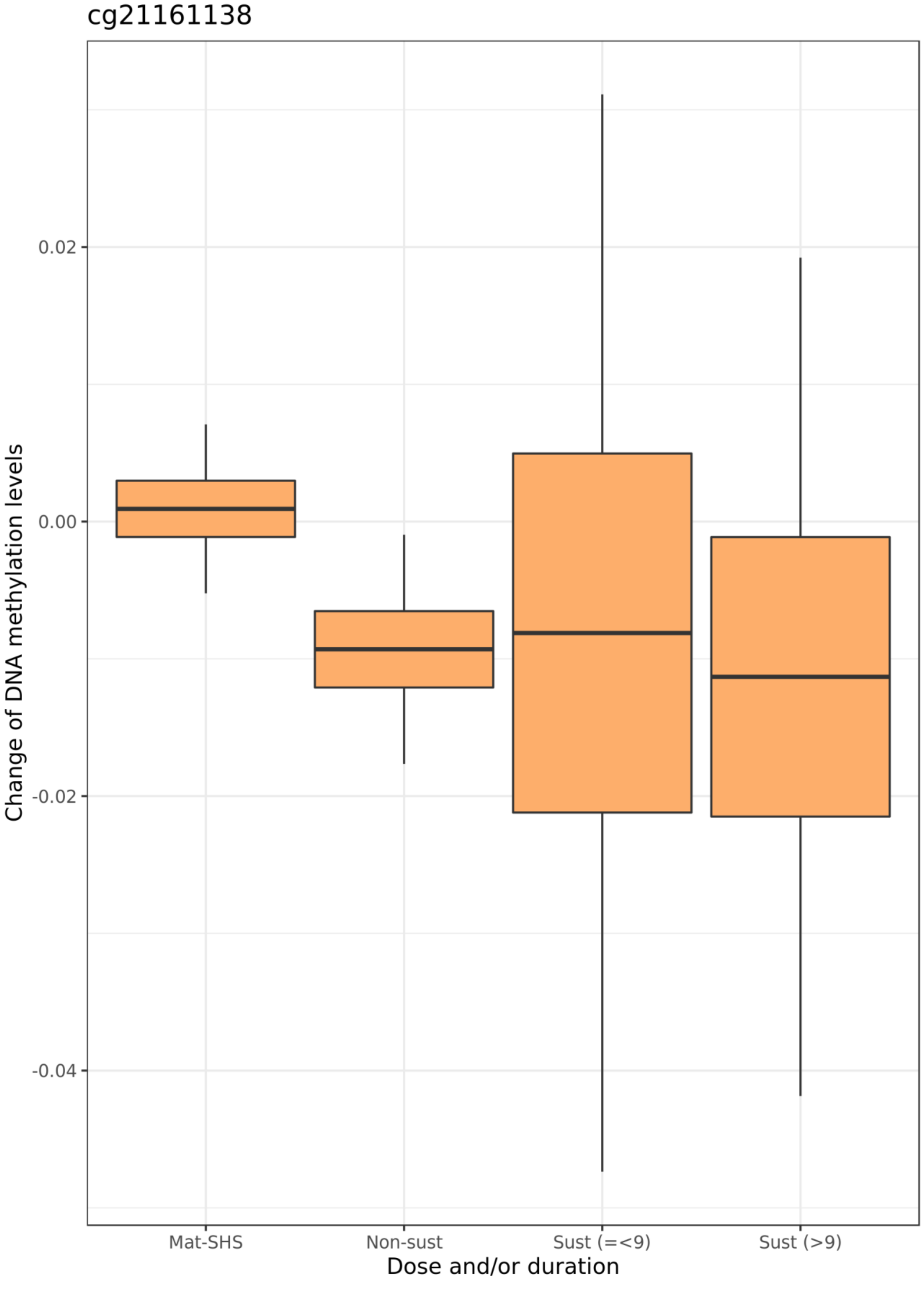
cg21161138
Change of DNA methylation levels
0.02
0.00
-0.02
-0.04
Mat-SHS
Non-sust
Sust (=<9)
Sust (>9)
Dose and/or duration

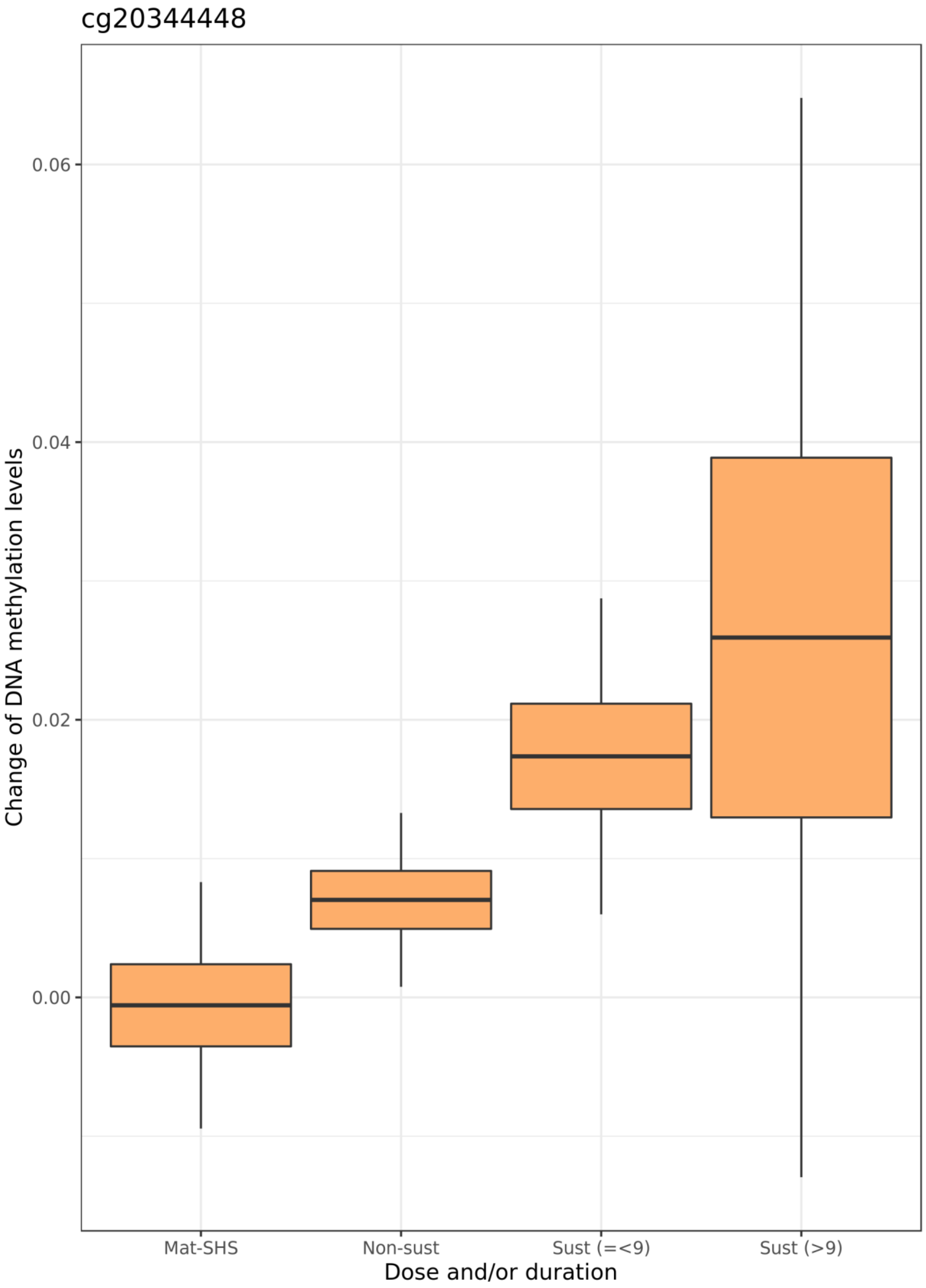
cg20344448
Change of DNA methylation levels
0.06
0.04
0.02
0.00
Mat-SHS
Non-sust
Sust (=<9)
Sust (>9)
Dose and/or duration

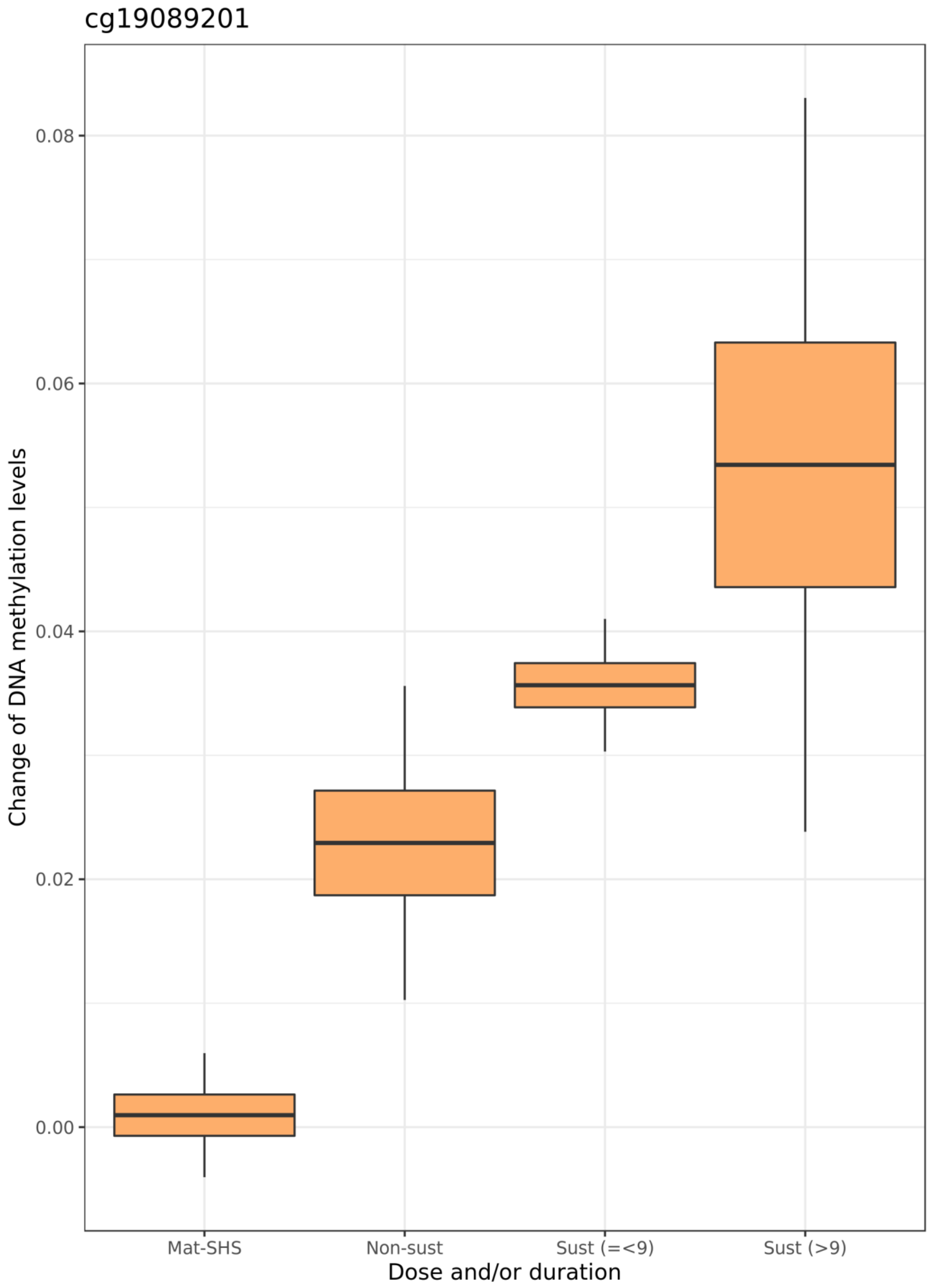
cg19089201
Change of DNA methylation levels
0.08
0.06
0.04
0.02
0.00
Mat-SHS
Non-sust
Sust (=<9)
Sust (>9)
Dose and/or duration

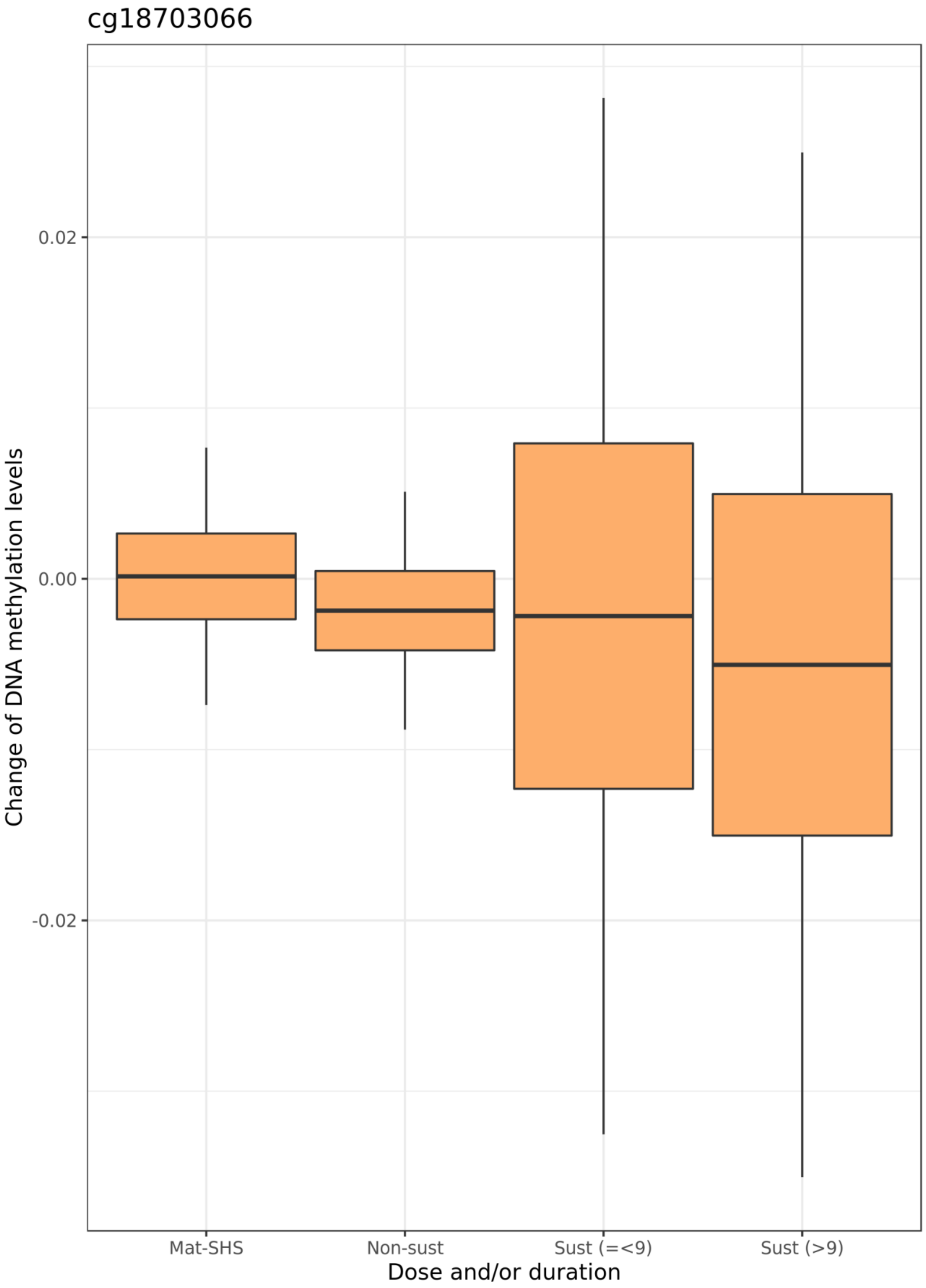
cg18703066
0.02
0.00
-0.02
Change of DNA methylation levels
Mat-SHS
Non-sust
Sust (=<9)
Sust (>9)
Dose and/or duration

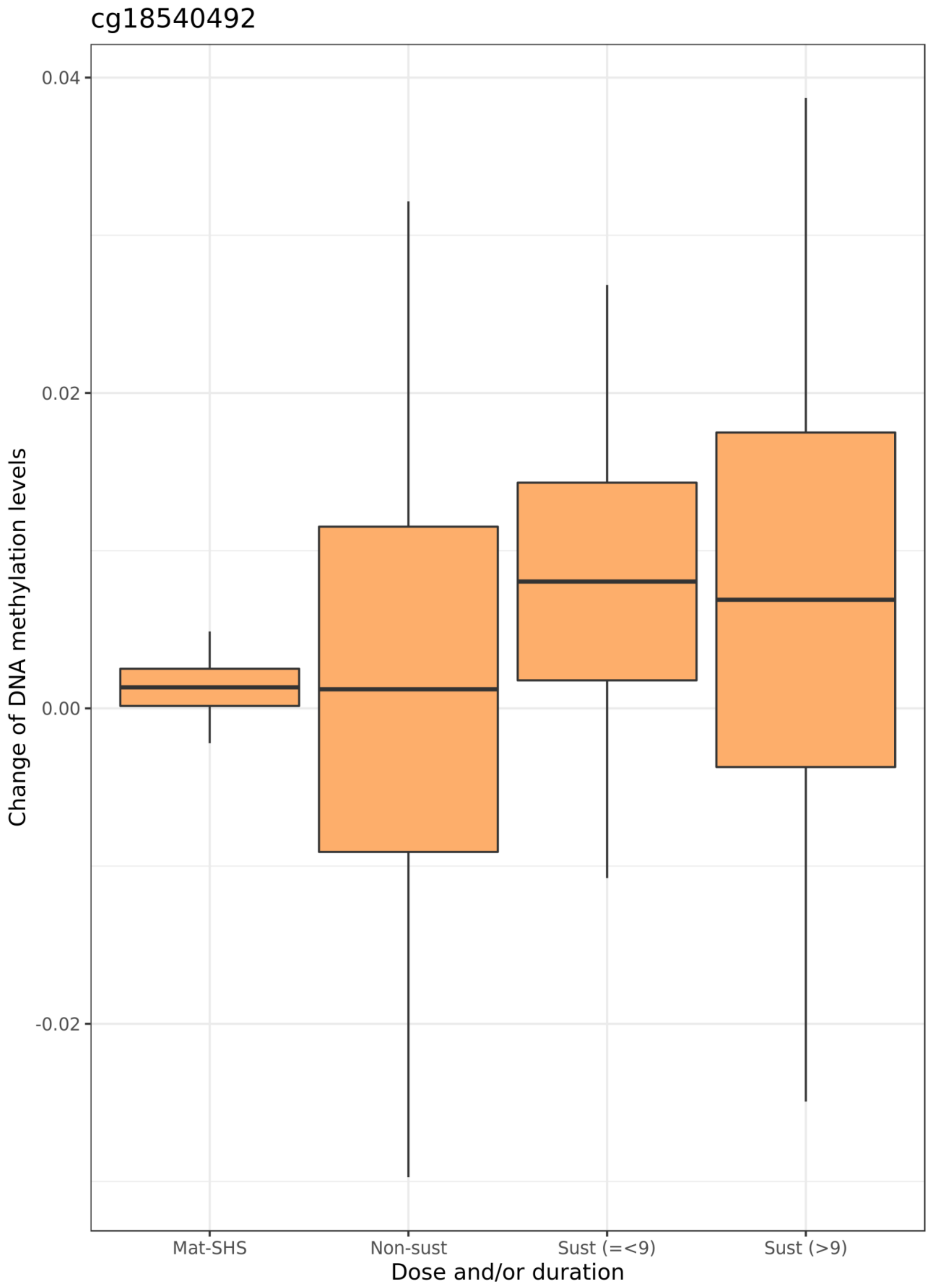

cg18540492
0.04
0.02
0.00
-0.02
Change of DNA methylation levels
Mat-SHS
Non-sust
Sust (=<9)
Sust (>9)
Dose and/or duration

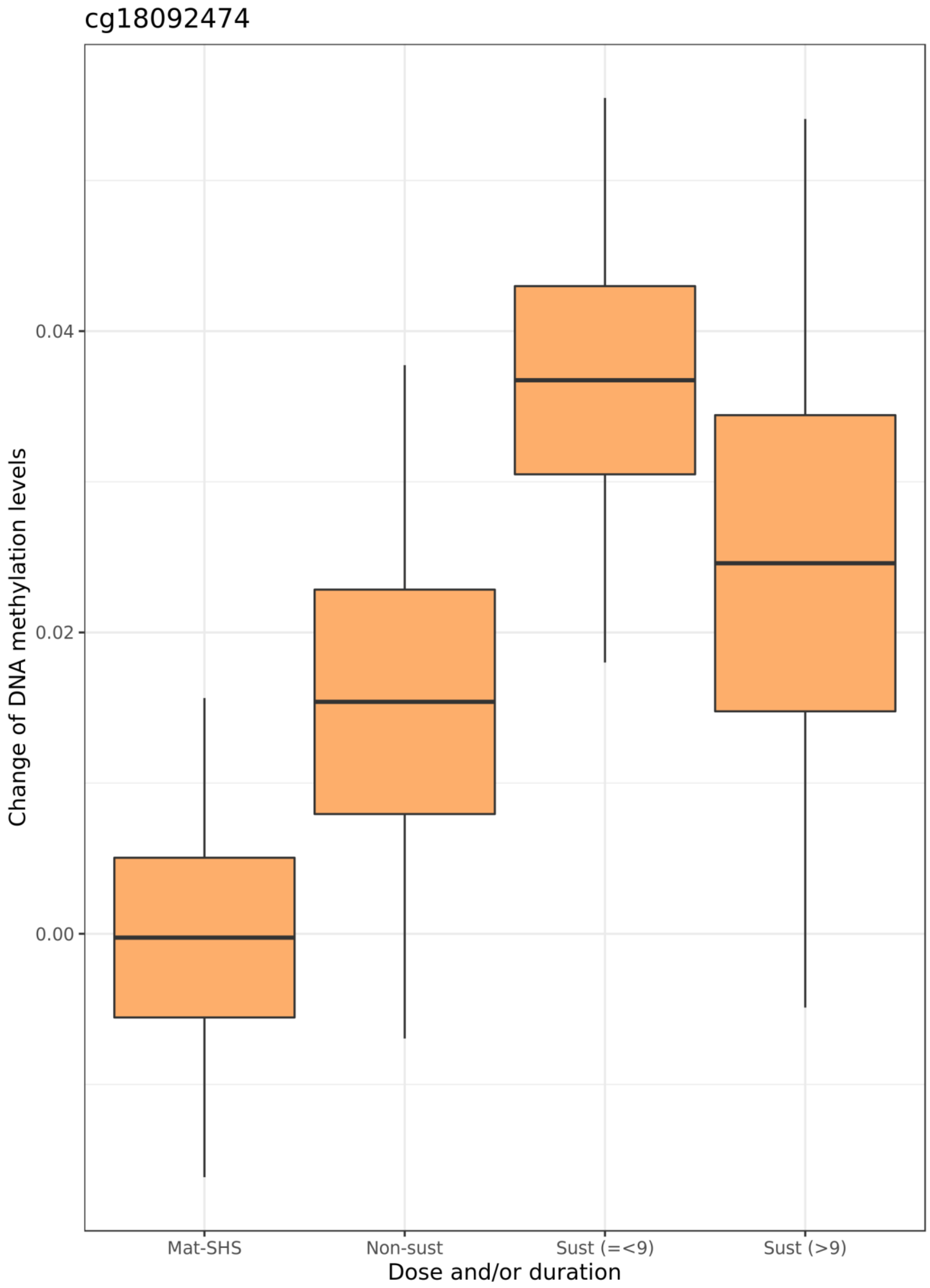
cg18092474
Change of DNA methylation levels
0.04
0.02
0.00
Mat-SHS
Non-sust
Sust (=<9)
Sust (>9)
Dose and/or duration

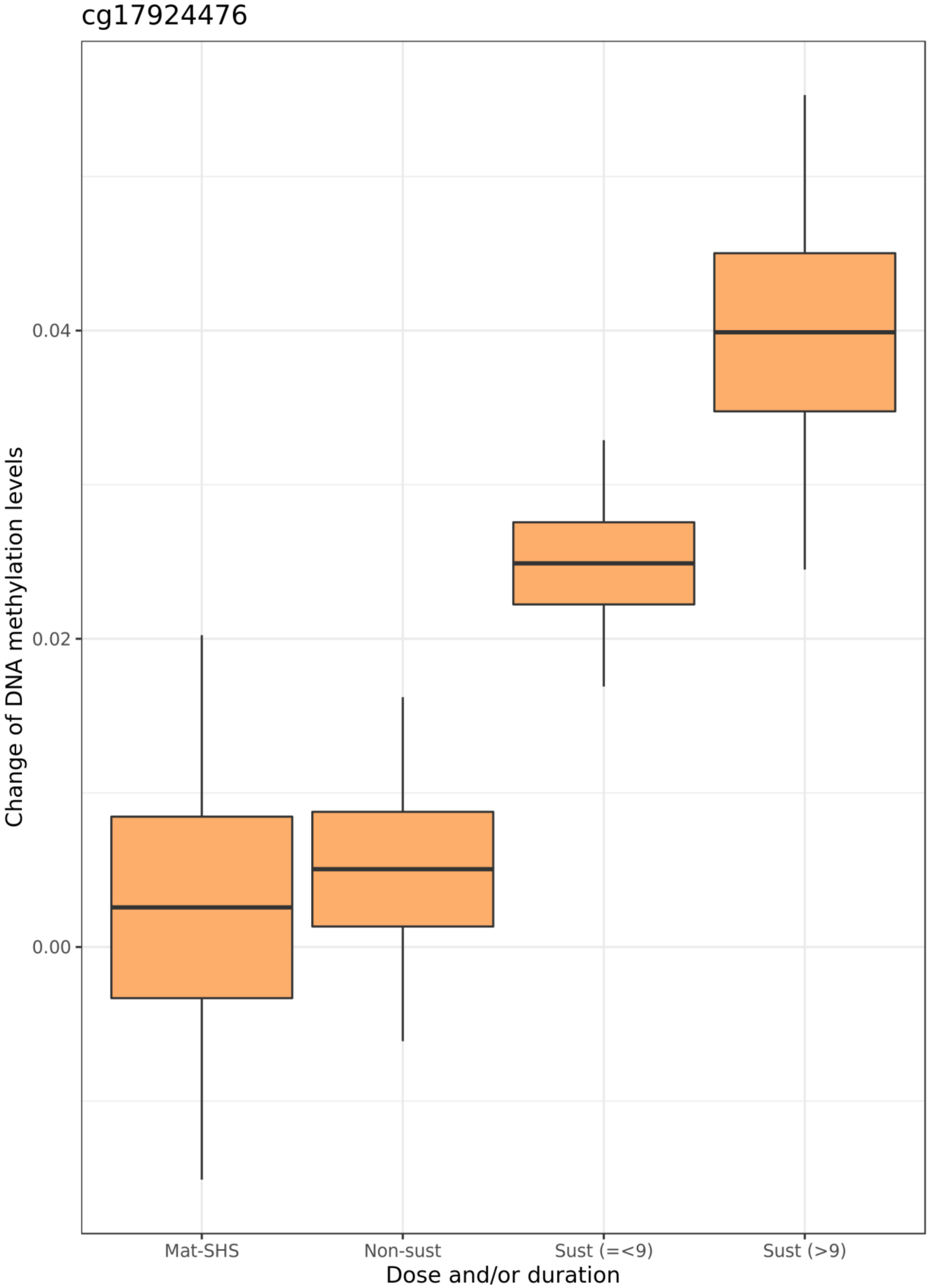

cg17924476
Change of DNA methylation levels
0.04
0.02
0.00
Mat-SHS
Non-sust
Sust (=<9)
Sust (>9)
Dose and/or duration

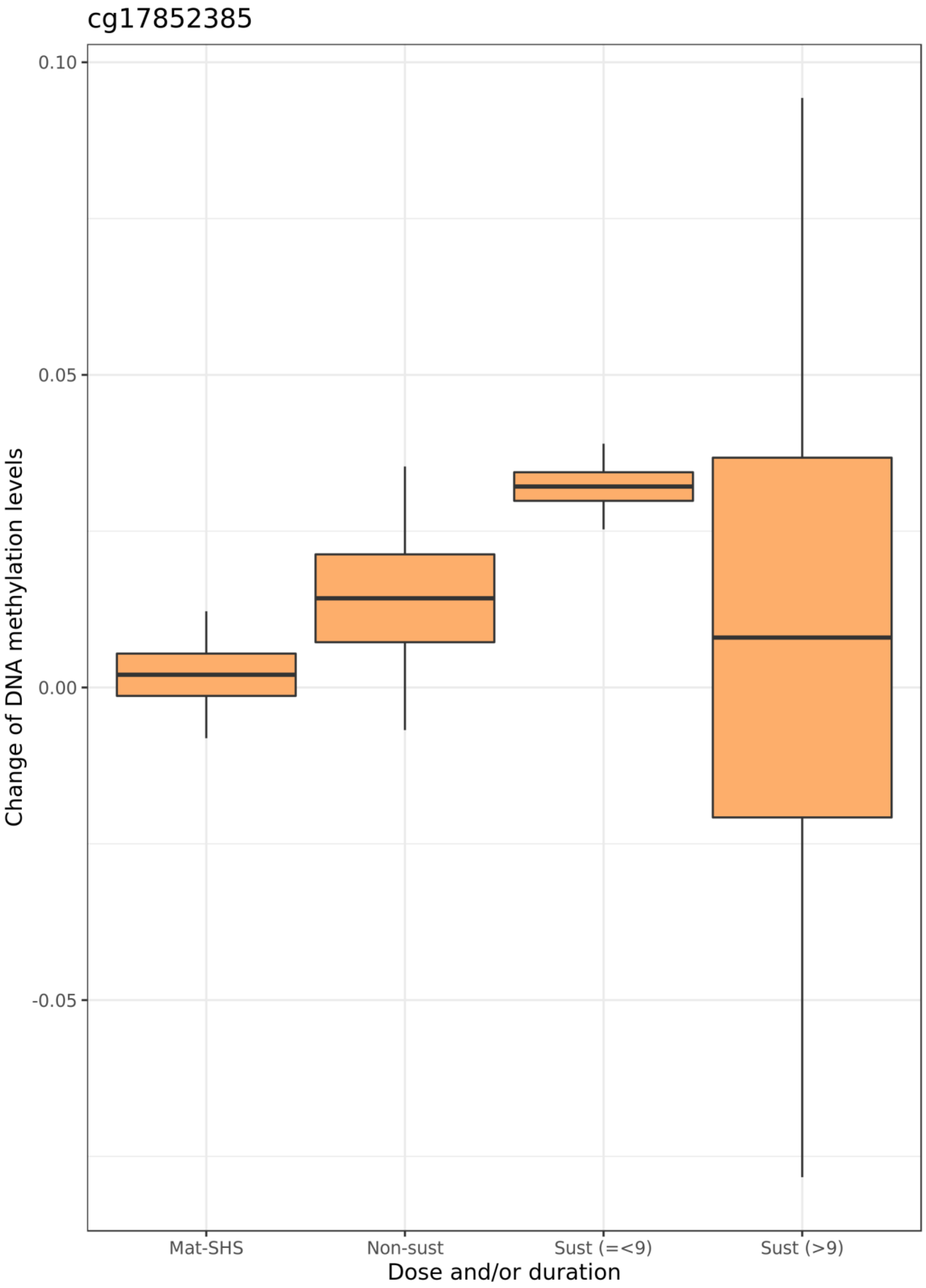
cg17852385
Change of DNA methylation levels
0.10
0.05
0.00
-0.05
Mat-SHS
Non-sust
Sust (=<9)
Sust (>9)
Dose and/or duration

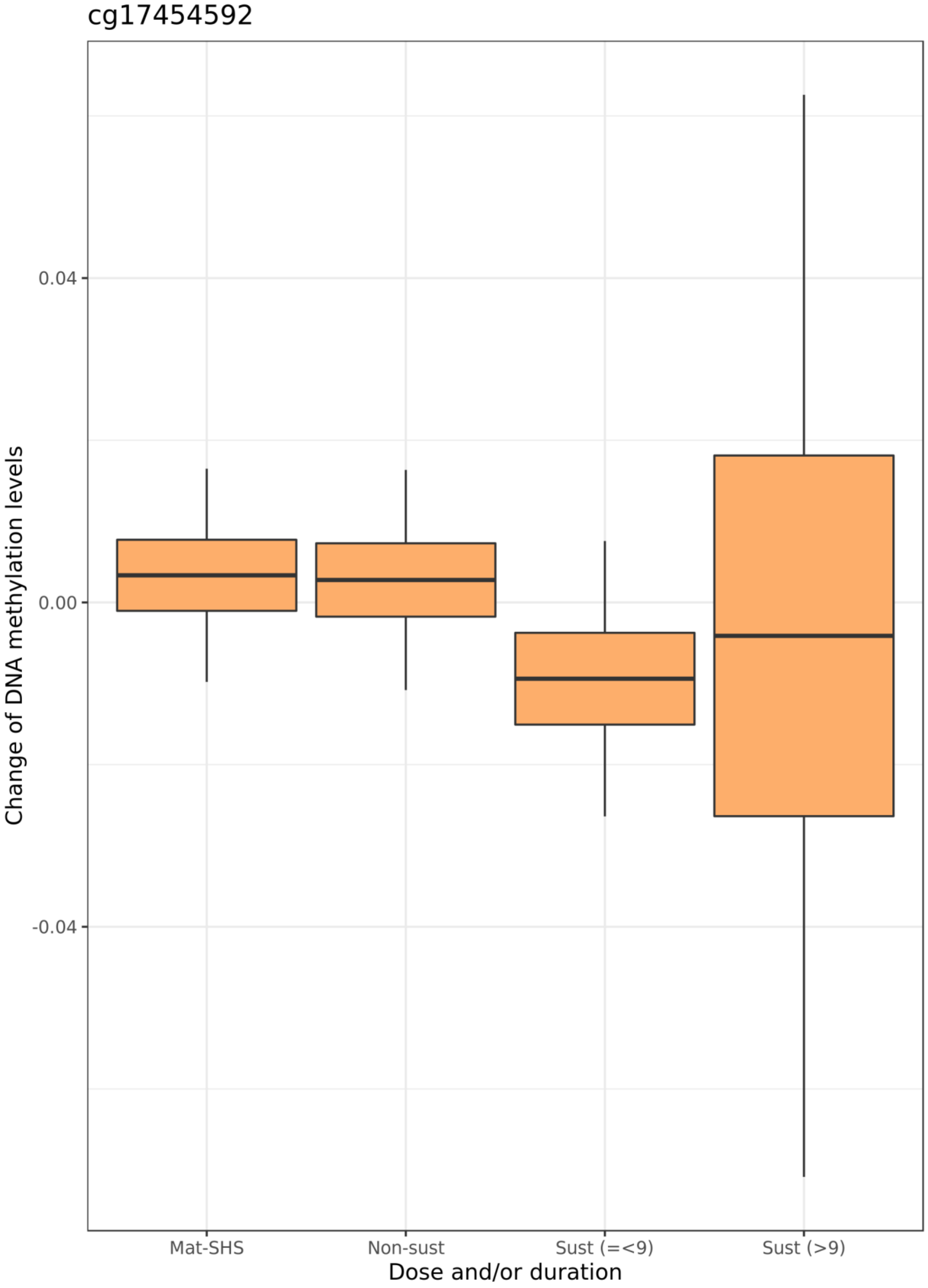

cg17454592
0.04
0.00
-0.04
Change of DNA methylation levels
Mat-SHS
Non-sust
Sust (=<9)
Sust (>9)
Dose and/or duration

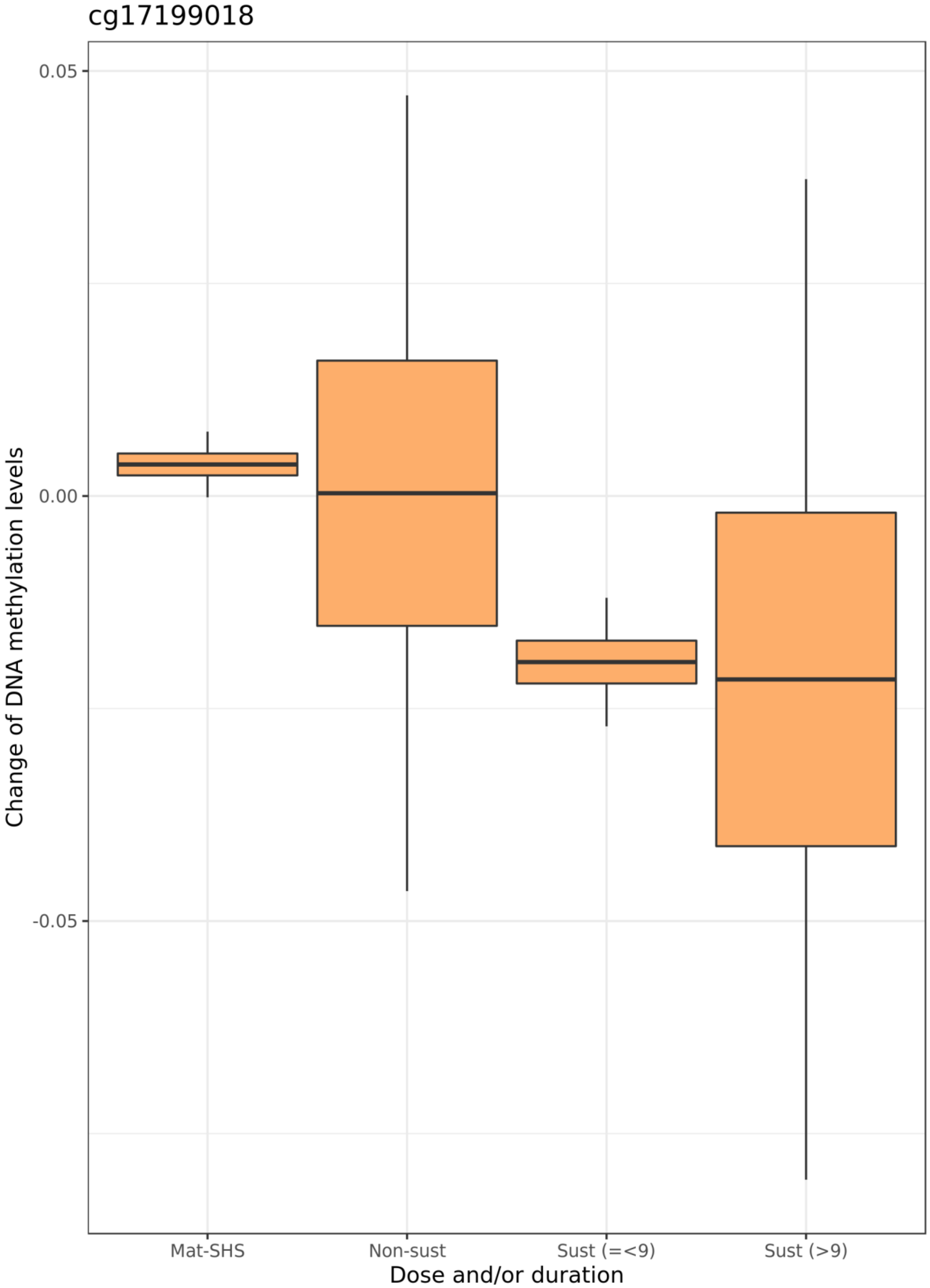

cg17199018
0.05
0.00
-0.05
Change of DNA methylation levels
Mat-SHS
Non-sust
Sust (=<9)
Sust (>9)
Dose and/or duration

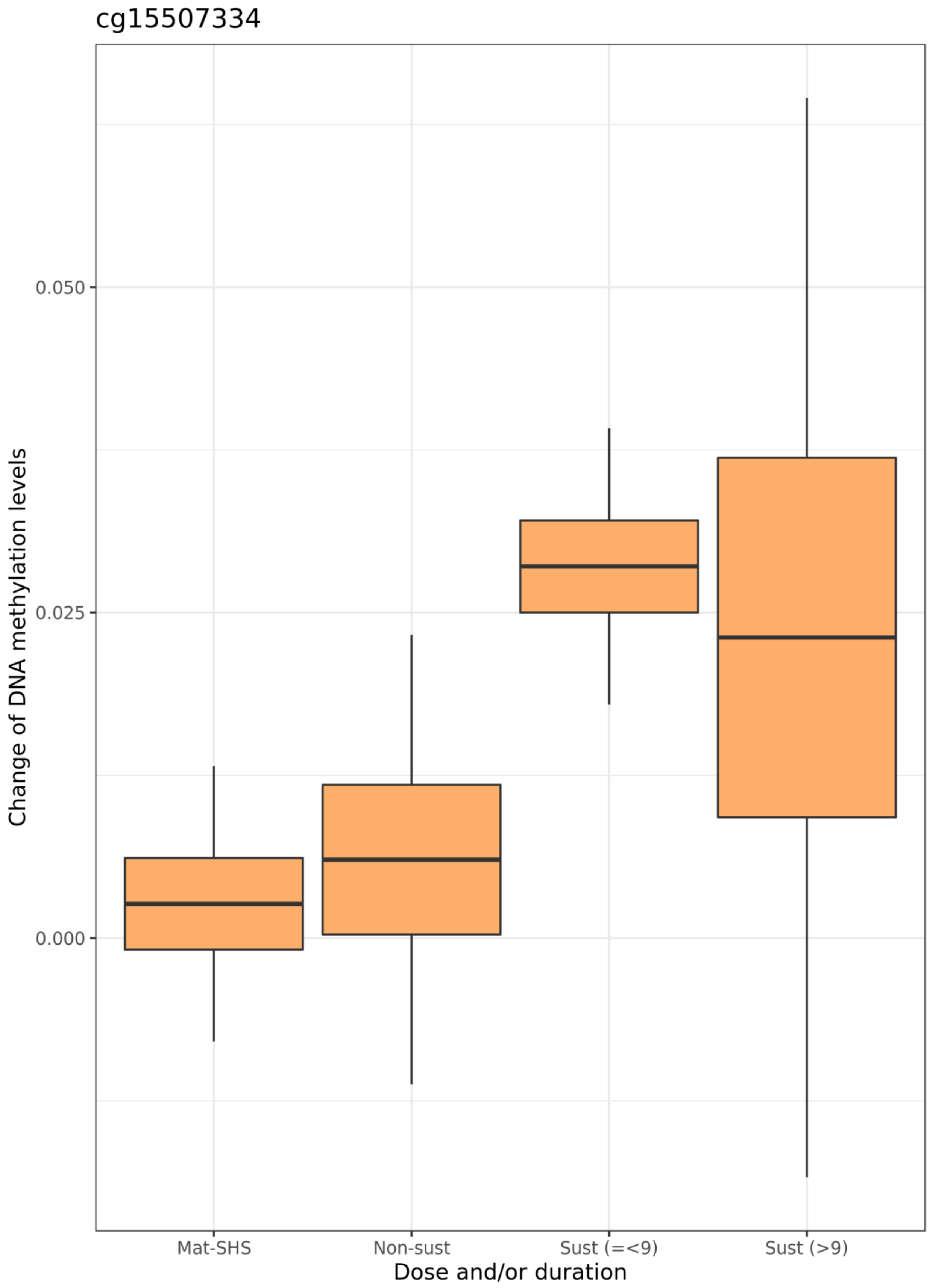

cg15507334
Change of DNA methylation levels
0.050
0.025
0.000
Mat-SHS
Non-sust
Sust (=<9)
Sust (>9)
Dose and/or duration

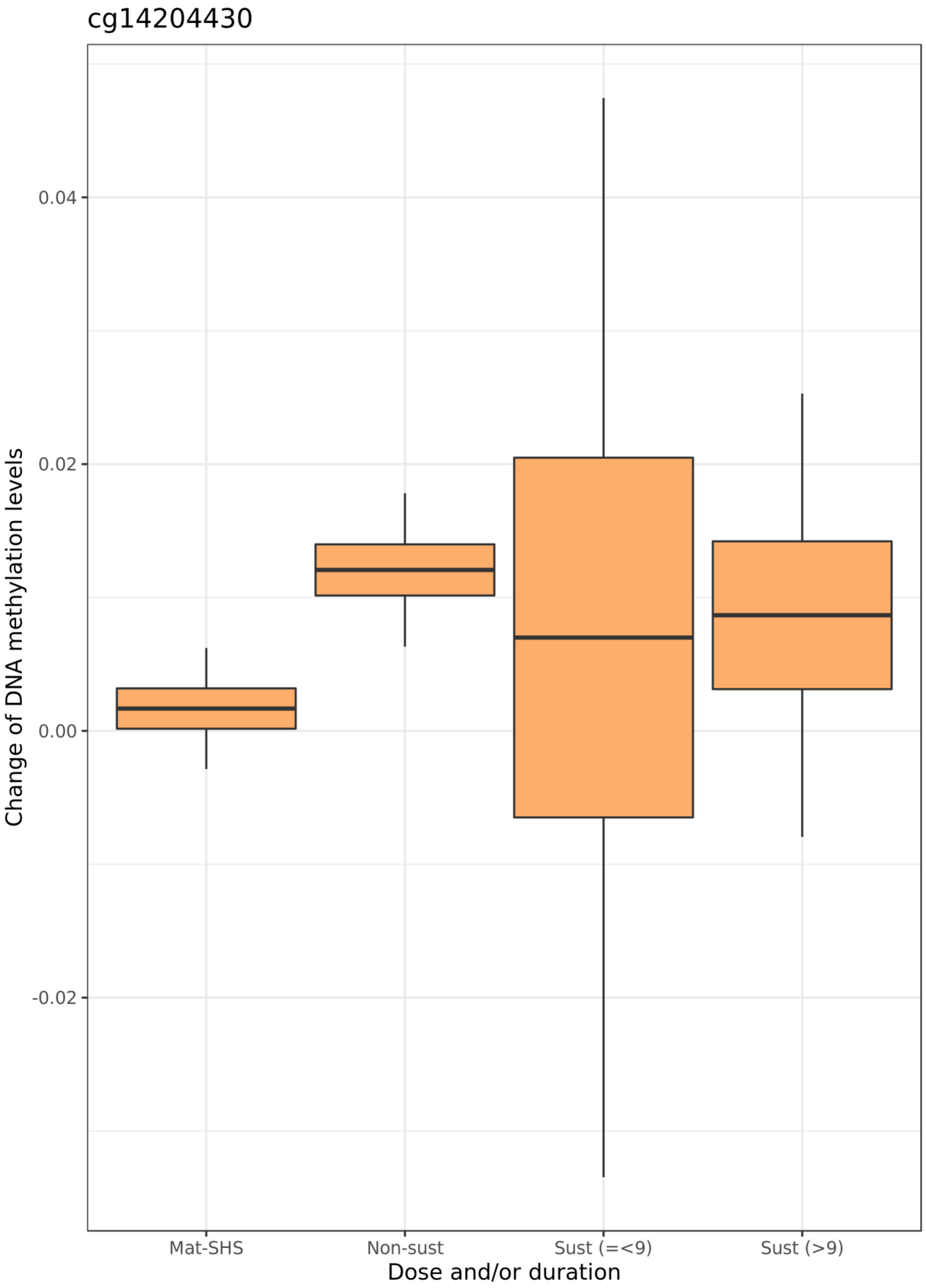
cg14204430
Change of DNA methylation levels
0.04
0.02
0.00
-0.02
Mat-SHS
Non-sust
Sust (=<9)
Sust (>9)
Dose and/or duration
